# Safety profile of fentanyl with different routes of administration: a disproportionality analysis using the EudraVigilance database

**DOI:** 10.1007/s00210-026-05145-8

**Published:** 2026-02-27

**Authors:** Raffaella Di Napoli, Annamaria Mascolo, Nunzia Balzano, Alessia Zinzi, Maria Maddalena Nicoletti, Annalisa Capuano, Francesca Gargano

**Affiliations:** 1 Department of Experimental Medicine, – Section of Pharmacology “L. Donatelli”, University of Campania “Luigi Vanvitelli”, Costantinopoli 16, 80138 Naples, Italy; 2 Campania Regional Centre for Pharmacovigilance and Pharmacoepidemiology, 80138 Naples, Italy; 3 Department of Life Science, Health, and Health Professions, Link Campus University, Rome, Italy; 4 Department of Precision Medicine, University of Campania “Luigi Vanvitelli”, 80138 Naples, Italy; 5 Unit of Anesthesia and Intensive Care, Fondazione Policlinico Universitario Campus Bio-Medico, 00128 Rome, Italy

**Keywords:** Fentanyl, Opioid, Pain, Safety, Pharmacovigilance, Adverse events, Disproportionality analysis

## Abstract

Fentanyl is associated with significant respiratory, cardiovascular, and neurological adverse events (AEs). This study aimed to describe the safety of fentanyl administered through different routes of administration, with a focus on brain lesions. Safety data related to fentanyl were extracted from the EudraVigilance database up to March 7, 2024. Descriptive and disproportionality analyses were performed. A total of 6,441 cases were classifiable for the administration route. The transdermal route (80.9%) was the most reported route. Most cases were from adults and females. Overall, 92.8% of AEs were serious. The intravenous route had a higher disproportionate reporting of “nervous system disorders” than transdermal (ROR 1.31; [95% CI, 1.12–1.41]) and oral transmucosal (1.30; [1.12–1.49]). Among central nervous system disorders, 50 brain lesions were identified, with no statistically significant difference observed. Nasal administration had a higher reporting frequency of “central nervous system vascular disorders” compared to transdermal (2.45; [1.14–5.24]), intravenous (2.53; [1.09–5.87]), and oral transmucosal routes (4.89; [1.54–15.51]). On the other hand, the intravenous had a higher disproportionate reporting of “seizures” compared to nasal (3.19; [1.17–8.69]), oral transmucosal (2.09; [1.31–3.32]), and transdermal routes (2.41; [1.96–2.96]). The study provides a comprehensive safety profile of fentanyl across different administration routes, highlighting a higher disproportionate reporting of central neurologic and cardiovascular AEs with nasal and intravenous routes.

**Supplementary Information:**

The online version contains supplementary material available at 10.1007/s00210-026-05145-8.

## Introduction

Fentanyl is a potent synthetic opioid derived from a synthetic phenylpiperidine with a binding structure that makes it at least 100 times more potent than morphine (Peng and Sandler 1999). Due to its proven efficacy in the treatment of pain, the pharmaceutical industry has increasingly focused on the development of non-parenteral formulations (Schug and Ting 2017; Cuomo 2023). These formulations were effective in treating various types of pain, including acute pain (such as post-operative discomfort), chronic pain (particularly in cancer patients), and breakthrough pain, which is a transitory flare of pain despite ongoing therapy for chronic pain (Schug and Ting 2017). Once fentanyl binds to μ-opioid receptors in the central and peripheral nervous systems, it causes analgesia but also sedation, euphoria, and respiratory depression. Its high lipophilicity allows it to cross the blood–brain barrier rapidly, resulting in a rapid onset of action. Fentanyl is available in different formulations, including injectable, oral transmucosal fentanyl citrate, buccal tablets, sublingual tablets, intranasal sprays, and fentanyl transdermal patches (FTPs) (Kuczyńska et al. 2018). Among all formulations, the intranasal administration has been ascribed as a non-invasive route of administration widely used to deliver drugs into the systemic circulation due to the high vascularity and permeability of the nasal mucosa that allow a rapid absorption (Mercadante et al. 2014). A recent research showed a greater preference for intranasal administration of fentanyl over the other routes of administration, probably due to its ease of administration and rapid onset of analgesia (Watts et al. 2013). One review also concluded that the intranasal administration route had the fastest onset of analgesia compared to the buccal route (Davis 2011). 

Despite its widespread use in clinical practice, fentanyl is associated with a spectrum of adverse events (AEs) similar to other opioids (Kornick et al. 2003; Schug and Ting 2017). To date, the study of Maosha Dai et al. has highlighted respiratory, cardiovascular, and neurological AEs following the use of fentanyl. Specifically, the authors identified a high disproportionate reporting of cardiorespiratory arrest (Reporting Odds Ratio, ROR 7.86; 95%CI: 7.28–8.5), opisthotonos (ROR 77.74; 95%CI 63.92–94.55), and apnoea (ROR 11.33; 95%CI: 9.81–13.08) following fentanyl use (Dai et al. 2023). An open-label study comparing oral transmucosal fentanyl citrate and nasal spray showed that the overall incidence of AEs was 35% and 46%, respectively. The most common AEs for both routes of administration were nausea, vomiting, and constipation (Mercadante et al. 2009). Among potential risks associated with fentanyl formulations, the risk of brain lesions is under evaluation in the Products’ Risk Management Plan of fentanyl. Brain lesions were firstly observed following the intravenous administration of high doses of fentanyl in rats (Kofke et al. 1996), and then as neuronal damage of rat spinal cord following the intrathecal administration of fentanyl (Abut et al. 2015). However, brain lesions were not observed in six-month rat and nine-month dog studies evaluating the intranasal fentanyl formulation (CHMP n.d.). The relevance of these findings on humans is currently unknown. Therefore, studies are needed to investigate the possible occurrence of this AE in humans to ensure a positive risk–benefit balance of fentanyl formulations. This pharmacovigilance study aimed to describe the safety profile of fentanyl administered through different routes of administration, with a focus on the disproportionate reporting of brain lesions by using data from the European pharmacovigilance database, EudraVigilance (EV).

## Methods

### Study design

The present study is an observational, retrospective, pharmacovigilance study designed to evaluate the safety profile of fentanyl administered through different routes of administration, with a focus on the disproportionate reporting of brain lesions. This study followed the Reporting of a Disproportionality Analysis for Drug Safety Signal Detection Using Individual Case Safety Reports in PharmacoVigilance (READUS‑PV) guideline (Fusaroli et al. 2024a, b).

### Data source

We retrieved all individual case safety reports (ICSRs) reporting fentanyl as a suspect drug from EV, a database accessible online via the European Medicines Agency (EMA) website at www.adrreports.eu (level of access dedicated to the stakeholder group II: healthcare professionals and the public). This is a registry managed by the EMA collecting ICSRs about medicines or vaccines submitted by healthcare professionals (HCP) or patients/citizens to a marketing authorization holder or a national competent authority. Serious and non serious ICSRs in EV can come from the European Economic Area (EEA), while only serious ICSRs come from the non-EEA.

### ICSRs selection

ICSRs related to fentanyl were retrieved up to March 7, 2024. Data extraction was performed using the standard EV query function, with fentanyl selected as the suspected drug. Prior to analysis, the data underwent a pre-processing phase to separate each information. 

ICSRs were classified based on the route of administration reported for fentanyl according with the summary of product characteristics (SmPCs) of each formulation. ICSRs reporting routes of administration different from those reported in SmPCs were excluded, as well as ICSRs not reporting a route of administration. Therefore, the transdermal, intravenous, intramuscular, transmucosal, sublingual, buccal, and intranasal routes were considered.

We also grouped ICSRs based on the route of administration as shown below:

- Buccal, transmucosal, and sublingual routes as *oral transmucosal*.
- Inhalation, nasal and respiratory routes as *nasal*.
- Cutaneous and transdermal routes as *transdermal*.

Despite the inhalation/respiratory and cutaneous routes do not exist among fentanyl formulations, they were still grouped into the nasal or transdermal group, respectively, because considered codification errors of the spontaneous reporting process.

A further distinction was made for ICSRs that indicated more than one route of administration:

- Reports with one correct and one incorrect route of administration were included, but only the correct route was considered valid.
- Reports with two or more correct routes of administration were grouped together and considered as *mixed*.

### Descriptive analysis

All ICSRs were described in terms of patient characteristics (sex and age group), type of reporter (HCP or non-HCP), and country for regulatory purposes (EEA or non-EEA). The number of other suspected drugs for fentanyl and the number of other concomitant drugs were reported. The concomitant drugs were also classified according to the Anatomical Therapeutic Chemical (ATC) system, which groups drugs by therapeutic use, chemical properties, and target organ/system. Our analysis categorised them up to the second ATC level, identifying their pharmacological or therapeutic subgroup.

The Medical Dictionary for Regulatory Activities (MedDRA) was used to classify AEs. The MedDRA is the international medical terminology developed by the International Council for Harmonisation of Technical Requirements for Pharmaceuticals for Human Use (ICH) that is used in pharmacovigilance data sources, such as EV. MedDRA consists of a hierarchical structure divided into five categories, from the most specific to the most general. This structure starts with the Lowest Level Term (LLT), followed by the Preferred Term (PT), the High-Level Group Term (HLGT), and the System Organ Class (SOC) (Ruggiero et al. 2023, 2024). The four most reported AEs for each SOC were tabled.

Considering that AEs are reported as PTs in EV, to identify ICSRs with events of “brain lesions”, the PTs “brain injury” and “central nervous system lesion” were used. Both PTs indeed referred to the most specific LLT “brain lesions”. Moreover, for a broader evaluation of “brain lesions”, the following MedDRA groups were included: the HLGT “central nervous system vascular disorder” (as events that can be potential causes of brain lesions), the HLGT “seizures” (as events that can be potential symptoms or consequences of brain lesions), and the SOC “nervous system disorders” (as events that can be potential symptoms of brain lesions).

Moreover, AEs were described in terms of their seriousness and outcome. An AE was classified as *serious* if it was “life-threatening”, “caused/prolonged hospitalisation”, “disabling”, “congenital anomaly”, “other medically important condition”, or “resulted in death”. This classification is based on the International Council on Harmonisation's E2D guidelines. The outcome of an AE was favourable if it was “recovered/resolved” or “recovering/resolving”, while it was considered unfavourable if it was “not recovered/not resolved”, “recovered/resolved with sequelae”, or “fatal”.

### Disproportionality analysis

A disproportionality analysis by using the Reporting Odds Ratio (ROR) and its 95% confidence interval (95% CI) was performed to identify aa positive signal for an AE. The ROR was calculated using the formula (a/c)/(b/d), where 'a' is the number of events reported with a route, 'c' is the number of events reported with another route, 'b' is the number of other events reported with a route, and 'd' is the number of other events reported with another route. Specifically, the RORs of the three most reported SOCs, the LLT *“brain lesions”,* the HLGT “*central nervous system vascular disorder”*, the HLGT “*seizures”*, the SOC *“nervous system disorders”* were computed by comparing all routes of fentanyl administration.

For the evaluation of brain lesions, a stratified disproportionality analysis by sex was performed by comparing male vs. female for each route of fentanyl administration.

Disproportionality analyses were performed only if at least 3 AEs were reported for each route of administration. Positive signals were identified using a 95% CI lower limit for ROR > 1. A p-value ≤ 0.05 was considered statistically significant. Data management and analyses were performed using Excel 365 (Microsoft Office) and R (version 4.3.2, R Development Core Team).

## Results

### Descriptive analysis of cases

During study period, we retrieved a total of 10,160 ICSRs reporting fentanyl as the suspected drug from the EV database, of which 6,441 cases were classified for the route of administration (N = 5,211, 80.9% transdermal; N = 777, 12.1% intravenous; N = 266, 4.1% oral transmucosal; N = 96, 1.5% mixed; N = 87, 1.3% nasal; and N = 4, 0.06% intramuscular; Supplementary Fig. 1). The first ICSR was observed on March 15, 2004. Overall, most ICSRs were related to adult and females (N = 3,137, 48.7% and N = 3,699, 57.4%, respectively). ICSRs were mainly reported by HCP (overall N = 4,651; 72.2%). Regarding the primary source country for regulatory purposes, the non-EEA was the most representative for the following routes of administration: transdermal, intravenous, oral transmucosal, and mixed (N = 3,564, 68.4%; N = 599, 77.1%; N = 188, 70.7%; N = 63, 65.6%, respectively). On the contrary, the EEA was the most representative for nasal route of administration (N = 60; 69.0%). In most cases, fentanyl was the only suspected drug (N = 2,539, 48.7% transdermal; N = 140, 52.6% oral transmucosal; N = 46, 52.9% nasal and N = 4, 100% intramuscular). For the mixed group, 39 (40.6%) ICSRs had more than 5 suspected drugs reported. The majority of ICSRs had no concomitant drug (overall N = 2,888; 44.8%). The main demographic and clinical characteristics of all ICSRs were presented in Table 1.

**Table 1:** Descriptive analysis of age, sex, source, country, number of suspects, and concomitants of individual case safety reports (ICSRs) with different routes of fentanyl administration, retrieved from the EudraVigilance spontaneous reporting system up to 7 March 2024. Data are expressed as N (%)

	Oral transmucosal (N = 266)	Intramuscular (N = 4)	Intravenous (N = 777)	Nasal (N = 87)	Mixed (N = 96)	Transdermal (N = 5211)	Overall (N = 6441)
Age							
0–1 Month	1 (0.4)	-	15 (1.9)	1 (1.1)	-	2 (0.0)	19 (0.3)
2 Months—2 Years	13 (4.9)	-	24 (3.1)	-	-	27 (0.5)	64 (1.0)
3–11 Years	1 (0.4)	-	40 (5.1)	-	-	20 (0.4)	61 (0.9)
12–17 Years	4 (1.5)	1 (25.0)	32 (4.1)	2 (2.3)	-	22 (0.4)	61 (0.9)
18–64 Years	136 (51.1)	2 (50.0)	442 (56.9)	58 (66.7)	57 (59.4)	2442 (46.9)	3137 (48.7)
More than 65 Years	76 (28.6)	1 (25.0)	162 (20.8)	17 (19.5)	37 (38.5)	2090 (40.1)	2383 (37.0)
Not Specified	35 (13.2)	-	62 (8.0)	9 (10.3)	2 (2.1)	608 (11.7)	716 (11.1)
Sex							
Female	145 (54.5)	2 (50.0)	401 (51.6)	43 (49.4)	45 (46.9)	3063 (58.8)	3699 (57.4)
Male	111 (41.7)	2 (50.0)	352 (45.3)	42 (48.3)	50 (52.1)	2034 (39.0)	2591 (40.2)
Not Specified	10 (3.8)	-	24 (3.1)	2 (2.3)	1 (1.0)	114 (2.2)	151 (2.3)
Source							
Healthcare Professional	219 (82.3)	4 (100)	736 (94.7)	78 (89.7)	88 (91.7)	3526 (67.7)	4651 (72.2)
Non Healthcare Professional	45 (16.9)	-	34 (4.4)	9 (10.3)	8 (8.3)	1667 (32.0)	1763 (27.4)
Not Specified	2 (0.8)	-	7 (0.9)	-	-	18 (0.3)	27 (0.4)
Country							
European Economic Area	78 (29.3)	2 (50.0)	178 (22.9)	60 (69.0)	33 (34.4)	1647 (31.6)	1998 (31.0)
Non European Economic Area	188 (70.7)	2 (50.0)	599 (77.1)	27 (31.0)	63 (65.6)	3564 (68.4)	4443 (69.0)
Suspects							
1	140 (52.6)	4 (100)	139 (17.9)	46 (52.9)	-	2539 (48.7)	2868 (44.5)
2	51 (19.2)	-	174 (22.4)	16 (18.4)	26 (27.1)	1434 (27.5)	1701 (26.4)
3	34 (12.8)	-	130 (16.7)	8 (9.2)	19 (19.8)	501 (9.6)	692 (10.7)
4	16 (6.0)	-	101 (13.0)	4 (4.6)	12 (12.5)	247 (4.7)	380 (5.9)
5 ≥	25 (9.4)	-	233 (30.0)	13 (14.9)	39 (40.6)	490 (9.4)	800 (12.4)
Concomitants							
0	96 (36.1)	2 (50.0)	354 (45.6)	52 (59.8)	37 (38.5)	2347 (45.0)	2888 (44.8)
1	28 (10.5)	-	87 (11.2)	10 (11.5)	11 (11.5)	529 (10.2)	665 (10.3)
2	19 (7.1)	1 (25.0)	47 (6.0)	4 (4.6)	6 (6.3)	416 (8.0)	493 (7.7)
3	28 (10.5)	-	60 (7.7)	2 (2.3)	6 (6.3)	332 (6.4)	428 (6.6)
4	18 (6.8)	1 (25.0)	43 (5.5)	4 (4.6)	5 (5.2)	267 (5.1)	338 (5.2)
5 ≥	77 (28.9)	-	186 (23.9)	15 (17.2)	31 (32.3)	1320 (25.3)	1629 (25.3)

The classification of concomitant drugs according to the second ATC level showed that analgesics (N02A: N = 2,346; 12.1%) and psycholeptics (N05B: N = 1,697; 8.7%) were the most reported (Supplementary Table 1).

### Descriptive analysis of adverse events

We analysed a total of 34,971 AEs related to fentanyl (N = 28,928, 82.7% transdermal; N = 3,779, 10.8% intravenous; N = 1,345, 3.8% oral transmucosal; N = 511, 1.46% mixed; N = 390, 1.1% nasal; N = 18, 0.05% intramuscular; Supplementary Table 2). Seriousness and outcome criteria are shown in Table 2.

**Table 2:** Descriptive analysis of seriousness and outcome criteria of adverse events reported with different routes of fentanyl administration retrieved from the EudraVigilance spontaneous reporting system up to 7 March 2024. Data are expressed as N (%)

	Oral transmucosal (N = 1345)	Intramuscular (N = 18)	Intravenous (N = 3779)	Nasal (N = 390)	Mixed (N = 511)	Transdermal (N = 28,928)	Overall (N = 34,971)
**Seriousness**							
Serious	1256 (93.4)	18 (100)	3625 (95.9)	306 (78.5)	485 (94.9)	26,686 (92.3)	32,376 (92.8)
Caused/Prolonged Hospitalisation	341 (25.4)	10 (55.6)	1382 (36.6)	93 (23.8)	246 (48.1)	11,033 (38.1)	13,105 (37.5)
Disabling	38 (2.8)	-	53 (1.4)	-	-	540 (1.9)	631 (1.8)
Life Threatening	115 (8.6)	-	677 (17.9)	63 (16.2)	7 (1.4)	1508 (5.2)	2370 (6.8)
Other Medically Important Condition	517 (38.4)	-	1200 (31.8)	101 (25.9)	118 (23.1)	9949 (34.4)	11,885 (34.0)
Results in Death	245 (18.2)	8 (44.4)	313 (8.3)	49 (12.6)	114 (22.3)	3655 (12.6)	4384 (12.5)
Congenital Anomaly	-	-	-	-	-	2 (0.0)	2 (0.0)
Not serious	89 (6.6)	-	154 (4.1)	84 (21.5)	26 (5.1)	2241 (7.7)	2594 (7.4)
**Outcome**							
Fatal	245 (18.2)	8 (44.4)	313 (8.3)	49 (12.6)	114 (22.3)	3655 (12.6)	4384 (12.5)
Not Recovered/Not Resolved	202 (15.0)	-	333 (8.8)	11 (2.8)	49 (9.6)	5057 (17.5)	5652 (16.2)
Not Specified	17 (1.3)	-	12 (0.3)	-	-	58 (0.2)	87 (0.2)
Recovered/Resolved	384 (28.6)	1 (5.6)	1740 (46.0)	145 (37.2)	164 (32.1)	9466 (32.7)	11,900 (34.0)
Recovered/Resolved With Sequelae	1 (0.1)	-	46 (1.2)	2 (0.5)	2 (0.4)	80 (0.3)	131 (0.4)
Recovering/Resolving	75 (5.6)	6 (33.3)	282 (7.5)	50 (12.8)	46 (9.0)	1838 (6.4)	2297 (6.6)
Unknown	421 (31.3)	3 (16.7)	1053 (27.9)	133 (34.1)	136 (26.6)	8774 (30.3)	10,520 (30.1)

Of all events, 92.8% were serious and the most reported seriousness criterion was "caused/prolonged hospitalisation" (N = 13,105; 37.5%), followed by "other medically important condition" (N = 11,885; 34.0%), and "resulted in death" (N = 4,384; 12.5%). The outcome was favourable for most AEs for all routes of administration (N = 14,197; 40.59% overall), resulting in complete resolution (N = 11,900; 34.0%) or resolving (N = 2,297; 6.6%). In terms of the MedDRA SOCs classification (Table 3), “nervous system disorders” (N = 9,455; 27%) was the most frequently reported, followed by “general disorders and administration site conditions” (N = 5,150; 14.7%), and “injury poisoning and procedural complications” (N = 3,448; 9.9%).

**Table 3:** Descriptive analysis of adverse events categorized by MedDRA System Organ Class (SOC) and reported in Individual Case Safety Reports (ICSRs) with different routes of Fentanyl administration. The most four reported Preferred Terms (PTs) were described for each SOC. Data were retrieved from the EudraVigilance spontaneous reporting system up to March 7^th^, 2024, and expressed as N (%)

	Oral transmucosal	Intramuscular	Intravenous	Nasal	Mixed	Transdermal	Overall
**Blood and lymphatic system disorders**	**4 (0.3)**	**-**	**35 (0.9)**	**1 (0.3)**	**3 (0.6)**	**91 (0.3)**	**134 (0.4)**
Anaemia	3 (75.0)	-	15 (42.9)	-	1 (33.3)	39 (42.9)	58 (43.3)
Coagulopathy	-	-	6 (17.1)	-	1 (33.3)	3 (3.3)	10 (7.5)
Disseminated intravascular coagulation	-	-	5 (14.3)	-	-	3 (3.3)	8 (6.0)
Leukocytosis	-	-	3 (8.6)	1 (100)	-	4 (4.4)	8 (6.0)
**Cardiac disorders**	**15 (1.1)**	**1 (5.6)**	**168 (4.4)**	**10 (2.6)**	**8 (1.6)**	**512 (1.8)**	**714 (2.0)**
Bradycardia	1 (6.7)	-	28 (16.7)	-	1 (12.5)	43 (8.4)	73 (10.2)
Palpitations	3 (20.0)	-	2 (1.2)	1 (10.0)	-	63 (12.3)	69 (9.7)
Tachycardia	5 (33.3)	-	40 (23.8)	2 (20.0)	1 (12.5)	63 (12.3)	111 (15.5)
Cardiac arrest	-	1 (100)	24 (14.3)	3 (30.0)	-	31 (6.1)	59 (8.3)
**Congenital, familial and genetic disorders**	**-**	**-**	**5 (0.1)**	**-**	**-**	**6 (0.0)**	**11 (0.0)**
Atrial septal defect	-	-	1 (20.0)	-	-	-	1 (9.1)
Cerebrovascular arteriovenous malformation	-	-	1 (20.0)	-	-	-	1 (9.1)
Arnold-Chiari malformation	-	-		-	-	1 (20.0)	1 (9.1)
Cardiac septal defect	-	-		-	-	1 (20.0)	1 (9.1)
**Ear and labyrinth disorders**	**3 (0.2)**	**-**	**6 (0.2)**	**5 (1.3)**	**-**	**100 (0.3)**	**114 (0.3)**
Vertigo	2 (66.7)	-	2 (33.3)	4 (80.0)	-	54 (54.0)	62 (54.4)
Tinnitus	-	-	3 (50.0)	-	-	15 (15.0)	18 (15.8)
Deafness	-	-	-	-	-	6 (6.0)	6 (5.3)
Hypoacusis	-	-	-	-	-	12 (12.0)	12 (10.5)
**Endocrine disorders**	**2 (0.1)**	**-**	**2 (0.1)**	**-**	**-**	**46 (0.2)**	**50 (0.1)**
Hypothyroidism	-	-	1 (50.0)	-	-	9 (19.6)	10 (20.0)
Endocrine disorder	-	-	-	-	-	5 (10.9)	5 (10.0)
Hypothalamo-pituitary disorder	-	-	-	-	-	4 (8.7)	4 (8.0)
Thyroid disorder	-	-	-	-	-	9 (19.6)	9 (18.0)
**Eye disorders**	**12 (0.9)**	**-**	**80 (2.1)**	**11 (2.8)**	**4 (0.8)**	**410 (1.4)**	**517 (1.5)**
Miosis	7 (58.3)	-	14 (17.9)	6 (54.5)	2 (50.0)	160 (39.2)	189 (36.8)
Vision blurred	1 (8.3)	-	4 (5.1)	-	1 (25.0)	62 (15.2)	68 (13.3)
Mydriasis	-	-	9 (11.5)	1 (9.1)	-	14 (3.4)	24 (4.7)
Visual impairment	-	-	4 (5.1)	-	-	44 (10.8)	48 (9.4)
**Gastrointestinal disorders**	**175 (13.0)**	**-**	**226 (6.0)**	**16 (4.1)**	**35 (6.8)**	**2343 (8.1)**	**2795 (8.0)**
Constipation	23 (13.2)	-	9 (4.0)	2 (12.5)	2 (5.7)	214 (9.1)	250 (9.0)
Diarrhoea	7 (4.0)	-	7 (3.1)	1 (6.3)	2 (5.7)	154 (6.6)	171 (6.1)
Nausea	41 (23.6)	-	48 (21.2)	4 (25.0)	10 (28.6)	702 (30.0)	805 (28.8)
Vomiting	30 (17.2)	-	44 (19.5)	7 (43.8)	7 (20.0)	517 (22.1)	605 (21.7)
**General disorders and administration site conditions**	**172 (12.8)**	**1 (5.6)**	**408 (10.8)**	**33 (8.5)**	**59 (11.5)**	**4477 (15.5)**	**5150 (14.7)**
Drug ineffective	12 (7.0)	-	27 (6.6)	2 (6.1)	4 (6.8)	390 (8.7)	435 (8.4)
Pain	24 (14.0)	-	21 (5.1)	2 (6.1)	5 (8.5)	420 (9.4)	472 (9.2)
Drug interaction	3 (1.7)	-	83 (20.3)	3 (9.1)	5 (8.5)	195 (4.4)	289 (5.6)
Asthenia	5 (2.9)	1 (100)	10 (2.5)	2 (6.1)	2 (3.4)	216 (4.8)	236 (4.6)
**Hepatobiliary disorders**	**5 (0.4)**	**-**	**14 (0.4)**	**2 (0.5)**	**5 (1.0)**	**90 (0.3)**	**116 (0.3)**
Hepatic failure	1 (20.0)	-	5 (35.7)	-	2 (40.0)	9 (10.0)	17 (14.7)
Cholelithiasis	-	-	1 (7.1)	-	1 (20.0)	8 (8.9)	10 (8.6)
Hepatic function abnormal	-	-	2 (14.3)	-	-	9 (10.0)	11 (9.5)
Liver disorder	-	-	1 (7.1)	-	-	13 (14.4)	14 (12.1)
**Immune system disorders**	**-**	**-**	**19 (0.5)**	**-**	**1 (0.2)**	**47 (0.2)**	**67 (0.2)**
Anaphylactic reaction	-	-	2 (10.5)	-	-	1 (2.1)	3 (4.5)
Anaphylactic shock	-	-	6 (31.6)	-	-	1 (2.1)	7 (10.4)
Drug hypersensitivity	-	-	5 (26.3)	-	-	13 (27.7)	18 (26.9)
Hypersensitivity	-	-	5 (26.3)	-	-	20 (42.6)	25 (37.3)
**Infections and infestations**	**28 (2.1)**	**-**	**64 (1.7)**	**5 (1.3)**	**11 (2.2)**	**574 (2.0)**	**682 (2.0)**
Pneumonia	3 (10.7)	-	4 (6.3)	1 (20.0)	3 (27.3)	124 (21.6)	135 (19.8)
Pneumonia aspiration	4 (14.3)	-	4 (6.3)	2 (40.0)	-	39 (6.8)	49 (7.2)
Infection	2 (7.1)	-	4 (6.3)	-	-	31 (5.4)	37 (5.4)
Urinary tract infection	-	-	1 (1.6)	-	1 (9.1)	39 (6.8)	41 (6.0)
**Injury, poisoning and procedural complications**	**146 (10.9)**	**4 (22.2)**	**258 (6.8)**	**71 (18.2)**	**42 (8.2)**	**2927 (10.1)**	**3448 (9.9)**
Overdose	9 (6.2)	-	26 (10.1)	13 (18.3)	15 (35.7)	452 (15.4)	515 (14.9)
Wrong technique in product usage process	2 (1.4)	-	2 (0.8)	1 (1.4)	2 (4.8)	344 (11.8)	351 (10.2)
Fall	5 (3.4)	-	6 (2.3)	1 (1.4)	1 (2.4)	263 (9.0)	276 (8.0)
Toxicity to various agents	10 (6.8)	-	11 (4.3)	6 (8.5)	2 (4.8)	195 (6.7)	224 (6.5)
**Investigations**	**39 (2.9)**	**-**	**226 (6.0)**	**7 (1.8)**	**27 (5.3)**	**991 (3.4)**	**1290 (3.7)**
Weight decreased	7 (17.9)	-	8 (3.5)	1 (14.3)	-	164 (16.5)	180 (14.0)
Blood pressure increased	-	-	20 (8.8)	-	1 (3.7)	71 (7.2)	92 (7.1)
Heart rate increased	2 (5.1)	-	14 (6.2)	-	1 (3.7)	48 (4.8)	65 (5.0)
Oxygen saturation decreased	3 (7.7)	-	28 (12.4)	2 (28.6)	1 (3.7)	40 (4.0)	74 (5.7)
**Metabolism and nutrition disorders**	**33 (2.5)**	**-**	**56 (1.5)**	**5 (1.3)**	**7 (1.4)**	**556 (1.9)**	**657 (1.9)**
Decreased appetite	11 (33.3)	-	8 (14.3)	2 (40.0)	2 (28.6)	194 (34.9)	217 (33.0)
Dehydration	8 (24.2)	-	3 (5.4)	-	-	91 (16.4)	102 (15.5)
Diabetes mellitus	2 (6.1)	-	2 (3.6)	-	1 (14.3)	23 (4.1)	28 (4.3)
Abnormal loss of weight	-	-	-	-	-	31 (5.6)	31 (4.7)
**Musculoskeletal and connective tissue disorders**	**26 (1.9)**	**-**	**113 (3.0)**	**4 (1.0)**	**8 (1.6)**	**876 (3.0)**	**1027 (2.9)**
Arthralgia	2 (7.7)	-	3 (2.7)	-	-	65 (7.5)	70 (6.9)
Back pain	5 (19.2)	-	11 (9.7)	-	2 (28.6)	96 (11.1)	114 (11.2)
Muscle spasms	4 (15.4)	-	11 (9.7)	1 (25.0)	-	109 (12.6)	125 (12.3)
Pain in extremity	2 (7.7)	-	1 (0.9)	1 (25.0)	1 (14.3)	71 (8.2)	76 (7.5)
**Neoplasms benign, malignant and unspecified (incl cysts and polyps)**	**46 (3.4)**	**-**	**8 (0.2)**	**3 (0.8)**	**11 (2.2)**	**301 (1.0)**	**369 (1.1)**
Breast cancer	6 (13.0)	-	-	-	-	14 (4.7)	20 (5.4)
Neoplasm malignant	3 (6.5)	-	-	-	-	18 (6.0)	21 (5.7)
Lung neoplasm malignant	-	-	-	-	1 (9.1)	15 (5.0)	16 (4.3)
Malignant neoplasm progression	3 (6.5)	-	1 (12.5)	1 (33.3)	-	69 (22.9)	74 (20.1)
**Nervous system disorders**	**358 (26.6)**	**8 (44.4)**	**1209 (32.0)**	**107 (27.4)**	**140 (27.4)**	**7633 (26.4)**	**9455 (27.0)**
Somnolence	86 (24.1)	1 (12.5)	67 (5.5)	14 (13.1)	40 (28.6)	1163 (15.3)	1371 (14.5)
Loss of consciousness	20 (5.6)	1 (12.5)	65 (5.4)	8 (7.5)	3 (2.1)	415 (5.4)	512 (5.4)
Dizziness	20 (5.6)	-	25 (2.1)	4 (3.7)	4 (2.9)	660 (8.7)	713 (7.6)
Tremor	15 (4.2)	1 (12.5)	40 (3.3)	2 (1.9)	3 (2.1)	363 (4.8)	424 (4.5)
**Pregnancy, puerperium and perinatal conditions**	**2 (0.1)**	**-**	**5 (0.1)**	**-**	**-**	**-**	**7 (0.0)**
Live birth	-	-	1 (20.0)	-	-	-	1 (14.3)
Postpartum haemorrhage	-	-	1 (20.0)	-	-	-	1 (14.3)
Premature delivery	-	-	2 (40.0)	-	-	-	2 (28.6)
Uterine hypotonus	-	-	1 (20.0)	-	-	-	1 (14.3)
**Product issues**	**10 (0.7)**	**-**	**1 (0.0)**	**3 (0.8)**	**2 (0.4)**	**655 (2.3)**	**671 (1.9)**
Product quality issue	1 (10.0)		-	2 (66.7)	-	48 (7.3)	51 (7.6)
Product adhesion issue	-		-	-	1 (50.0)	156 (23.8)	157 (23.4)
Device leakage	-		-	-	-	34 (5.2)	34 (5.1)
Product complaint	-		-	-	-	276 (42.1)	276 (41.1)
**Psychiatric disorders**	**133 (9.9)**	**1 (5.6)**	**264 (7.0)**	**50 (12.8)**	**87 (17.0)**	**2862 (9.9)**	**3397 (9.7)**
Anxiety	10 (7.5)	-	10 (3.8)	2 (4.0)	4 (4.6)	210 (7.3)	236 (6.9)
Confusional state	14 (10.5)	1 (100)	18 (6.8)	3 (6.0)	5 (5.7)	373 (13.0)	414 (12.2)
Hallucination	4 (3.0)	-	9 (3.4)	2 (4.0)	3 (3.4)	167 (5.8)	185 (5.4)
Insomnia	6 (4.5)	-	16 (6.1)	-	3 (3.4)	239 (8.4)	264 (7.8)
**Renal and urinary disorders**	**13 (1.0)**	**-**	**39 (1.0)**	**-**	**5 (1.0)**	**320 (1.1)**	**377 (1.1)**
Acute kidney injury	1 (7.7)		7 (17.9)		-	45 (14.1)	53 (14.1)
Renal failure	3 (23.1)		3 (7.7)		2 (40.0)	48 (15.0)	56 (14.9)
Urinary retention	4 (30.8)		2 (5.1)		-	49 (15.3)	55 (14.6)
Urinary incontinence	-		1 (2.6)		1 (20.0)	27 (8.4)	29 (7.7)
**Reproductive system and breast disorders**	**8 (0.6)**	**-**	**25 (0.7)**	**-**	**-**	**34 (0.1)**	**67 (0.2)**
Pelvic pain	1 (12.5)	-	1 (4.0)	-	-	3 (8.8)	5 (7.5)
Female genital tract fistula	-	-	7 (28.0)	-	-	-	7 (10.4)
Vaginal discharge	-	-	7 (28.0)	-	-	1 (2.9)	8 (11.9)
Vaginal flatulence	-	-	7 (28.0)	-	-	-	7 (10.4)
**Respiratory, thoracic and mediastinal disorders**	**43 (3.2)**	**2 (11.1)**	**311 (8.2)**	**36 (9.2)**	**38 (7.4)**	**1412 (4.9)**	**1842 (5.3)**
Dyspnoea	9 (20.9)	2 (100)	31 (10.0)	1 (2.8)	3 (7.9)	303 (21.5)	349 (19.0)
Respiratory arrest	2 (4.7)	-	28 (9.0)	2 (5.6)	1 (2.6)	67 (4.8)	100 (5.4)
Respiratory depression	12 (27.9)	-	52 (16.8)	7 (19.4)	12 (31.6)	225 (16.0)	308 (16.7)
Respiratory failure	2 (4.7)	-	11 (3.5)	3 (8.3)	1 (2.6)	76 (5.4)	93 (5.1)
**Skin and subcutaneous tissue disorders**	**33 (2.5)**	**-**	**72 (1.9)**	**6 (1.5)**	**-**	**849 (2.9)**	**960 (2.7)**
Hyperhidrosis	16 (48.5)	-	24 (33.3)	4 (66.7)		369 (43.5)	413 (43.0)
Pruritus	9 (27.3)	-	9 (12.5)	-		89 (10.5)	107 (11.1)
Rash	1 (3.0)	-	7 (9.7)	-		62 (7.3)	70 (7.3)
Cold sweat	2 (6.1)	-	-	-		51 (6.0)	53 (5.5)
**Social circumstances**	**6 (0.4)**	**-**	**11 (0.3)**	**4 (1.0)**	**3 (0.6)**	**167 (0.6)**	**191 (0.5)**
Bedridden	2 (33.3)	-	1 (9.1)	-	-	26 (15.6)	29 (15.2)
Drug diversion	3 (50.0)	-	3 (27.3)	2 (50.0)	-	23 (13.8)	31 (16.2)
Loss of personal independence in daily activities	1 (16.7)	-	1 (9.1)	-	-	35 (21.0)	37 (19.4)
Disability	-	-	1 (9.1)	-	-	23 (13.8)	24 (12.6)
**Surgical and medical procedures**	**8 (0.6)**	**-**	**19 (0.5)**	**1 (0.3)**	**5 (1.0)**	**182 (0.6)**	**215 (0.6)**
Hospitalisation	2 (25.0)	-	-	-	-	42 (23.1)	44 (20.5)
Hip arthroplasty	-	-	1 (5.3)	-	-	6 (3.3)	7 (3.3)
Surgery	-	-	-	-	1 (20.0)	19 (10.4)	20 (9.3)
Spinal operation	-	-	-	-	-	10 (5.5)	10 (4.7)
**Vascular disorders**	**25 (1.9)**	**1 (5.6)**	**135 (3.6)**	**10 (2.6)**	**10 (2.0)**	**466 (1.6)**	**647 (1.9)**
Cyanosis	1 (4.0)	1 (100)	12 (9.0)	6 (60.0)	2 (20.0)	34 (7.3)	56 (8.7)
Hot flush	1 (4.0)	-	1 (0.7)	-	1 (10.0)	27 (5.8)	30 (4.7)
Hypertension	3 (12.0)	-	26 (19.4)	1 (10.0)	1 (10.0)	109 (23.4)	140 (21.7)
Hypotension	8 (32.0)	-	48 (35.8)	-	2 (20.0)	134 (28.8)	192 (29.8)

#### Descriptive analysis of brain lesions

A total of 50 brain lesions was found, corresponding to 47 brain injuries mostly reported with transdermal fentanyl (N = 36; 0.5%) than the other routes (oral transmucosal N = 1, 0.3%; intravenous N = 8, 0.7%; nasal N = 1, 0.9%; mixed N = 1, 0.7%) and 3 central nervous system lesions (oral transmucosal N = 1, 0.3%; transdermal N = 2, 0.0%; **Supplementary Table 2**). For brain injury, the seriousness criterion most reported was hospitalisation/prolonged hospitalisation (N = 19; 40.4%), while for the outcome the most reported was death (N = 15; 31.9%). In addition, fentanyl was the only suspected drug for 25 AEs. Among the three central nervous system lesions, two reported caused/prolonged hospitalisation (66.7%) and one resulted in death (33.3%). No other suspected drug was reported with these AEs, but 2 of them reported at least one concomitant drug.

#### Descriptive analysis of the SOC “nervous system disorders”, the HLGTs “central nervous system vascular disorders” and “Seizures”

Regarding the SOC “nervous system disorders” (Table 3), the PT most reported was “somnolence” (N = 1,371; 14.5%), followed by “loss of consciousness” (N = 512; 5.4%), and dizziness (N = 713; 7.6%). 

For HLGT, there were 255 AEs related to “central nervous system vascular disorders” and 548 AEs related to “seizures”. The PTs most reported were cerebrovascular accident (N = 130; 51.0%), transient ischaemic attack (N = 27; 10.6%), and cerebral haemorrhage (N = 22; 8.6%) for the HLGT “central nervous system vascular disorders”. On the other hand, the most reported PTs for the HLGT “seizures” were seizure (N = 398; 72.6%), generalised tonic–clonic seizure (N = 55; 10.0%), and epilepsy (N = 18; 3.3%). All PTs observed for HLGTs “seizures” and “central nervous system vascular disorders” are reported in Tables 4 and 5, respectively.

**Table 4:** Descriptive analysis adverse events grouped in the High-Level Group Term (HLGT) “Central nervous system vascular disorders” for each route of fentanyl administration. Data were retrieved from the EudraVigilance spontaneous reporting system up to March 7^th^, 2024, and expressed as N (%)

	Oral transmucosal (N = 5)	Intravenous (N = 27)	Nasal (N = 7)	Mixed (N = 2)	Transdermal (N = 214)	Overall (N = 255)
**Central nervous system vascular disorders**						
Cerebrovascular accident	3 (60.0)	2 (7.4)	3 (42.9)	2 (100)	120 (56.1)	130 (51.0)
Intracranial aneurysm	1 (20.0)	-	-	-	9 (4.2)	10 (3.9)
Transient ischaemic attack	1 (20.0)	1 (3.7)	1 (14.3)	-	24 (11.2)	27 (10.6)
Brain hypoxia	-	2 (7.4)	-	-	4 (1.9)	6 (2.4)
Cerebellar haemorrhage	-	2 (7.4)	-	-	-	2 (0.8)
Cerebellar infarction	-	1 (3.7)	-	-	1 (0.5)	2 (0.8)
Cerebral haematoma	-	1 (3.7)	-	-	-	1 (0.4)
Cerebral haemorrhage	-	3 (11.1)	1 (14.3)	-	18 (8.4)	22 (8.6)
Cerebral infarction	-	7 (25.9)	-	-	8 (3.7)	15 (5.9)
Cerebrovascular disorder	-	1 (3.7)	-	-	3 (1.4)	4 (1.6)
Embolic stroke	-	1 (3.7)	-	-	-	1 (0.4)
Intraventricular haemorrhage	-	2 (7.4)	-	-	-	2 (0.8)
Spinal cord infarction	-	1 (3.7)	-	-	-	1 (0.4)
Spinal epidural haematoma	-	1 (3.7)	-	-	-	1 (0.4)
Subarachnoid haemorrhage	-	2 (7.4)	-	-	2 (0.9)	4 (1.6)
Carotid artery stenosis	-	-	1 (14.3)	-	1 (0.5)	2 (0.8)
Ischaemic stroke	-	-	1 (14.3)	-	2 (0.9)	3 (1.2)
Basal ganglia infarction	-	-	-	-	1 (0.5)	1 (0.4)
Brain stem infarction	-	-	-	-	1 (0.5)	1 (0.4)
Brain stem ischaemia	-	-	-	-	1 (0.5)	1 (0.4)
Carotid artery dissection	-	-	-	-	1 (0.5)	1 (0.4)
Carotid artery occlusion	-	-	-	-	2 (0.9)	2 (0.8)
Cerebral hypoperfusion	-	-	-	-	1 (0.5)	1 (0.4)
Cerebral ischaemia	-	-	-	-	5 (2.3)	5 (2.0)
Cerebral small vessel ischaemic disease	-	-	-	-	1 (0.5)	1 (0.4)
Cerebral thrombosis	-	-	-	-	1 (0.5)	1 (0.4)
Embolic cerebral infarction	-	-	-	-	1 (0.5)	1 (0.4)
Haemorrhage intracranial	-	-	-	-	2 (0.9)	2 (0.8)
Haemorrhagic stroke	-	-	-	-	1 (0.5)	1 (0.4)
Intracranial haematoma	-	-	-	-	1 (0.5)	1 (0.4)
Lacunar infarction	-	-	-	-	2 (0.9)	2 (0.8)
Ruptured cerebral aneurysm	-	-	-	-	1 (0.5)	1 (0.4)

**Table 5:** Descriptive analysis of adverse events grouped in the High-Level Group Term (HLGT) “Seizures” for each route of fentanyl administration. Data were retrieved from the EudraVigilance spontaneous reporting system up to March 7th, 2024, and expressed as N (%)

	Oral transmucosal (N = 21)	Intramuscular (N = 1)	Intravenous (N = 121)	Nasal (N = 4)	Mixed (N = 9)	Transdermal (N = 392)	Overall (N = 548)
**Seizures (incl subtypes)**							
Drug withdrawal convulsions	1 (4.8)	-	1 (0.8)	-	-	13 (3.3)	15 (2.7)
Generalised tonic–clonic seizure	2 (9.5)	-	25 (20.7)	-	-	28 (7.1)	55 (10.0)
Seizure	18 (85.7)	1 (100)	64 (52.9)	3 (75.0)	8 (88.9)	304 (77.6)	398 (72.6)
Clonic convulsion	-	-	3 (2.5)	-	-	3 (0.8)	6 (1.1)
Dreamy state	-	-	1 (0.8)	-	-	3 (0.8)	4 (0.7)
Epilepsy	-	-	6 (5.0)	1 (25.0)	-	11 (2.8)	18 (3.3)
Febrile convulsion	-	-	2 (1.7)	-	-	-	2 (0.4)
Myoclonic epilepsy	-	-	2 (1.7)	-	-	-	2 (0.4)
Partial seizures	-	-	2 (1.7)	-	-	2 (0.5)	4 (0.7)
Petit mal epilepsy	-	-	1 (0.8)	-	1 (11.1)	5 (1.3)	7 (1.3)
Psychogenic seizure	-	-	1 (0.8)	-	-	1 (0.3)	2 (0.4)
Seizure cluster	-	-	1 (0.8)	-	-	-	1 (0.2)
Seizure like phenomena	-	-	1 (0.8)	-	-	-	1 (0.2)
Status epilepticus	-	-	5 (4.1)	-	-	7 (1.8)	12 (2.2)
Tonic clonic movements	-	-	4 (3.3)	-	-	2 (0.5)	6 (1.1)
Tonic convulsion	-	-	2 (1.7)	-	-	5 (1.3)	7 (1.3)
Atonic seizures	-	-	-	-	-	1 (0.3)	1 (0.2)
Convulsions local	-	-	-	-	-	2 (0.5)	2 (0.4)
Focal dyscognitive seizures	-	-	-	-	-	2 (0.5)	2 (0.4)
Generalised onset non-motor seizure	-	-	-	-	-	2 (0.5)	2 (0.4)
Hypoglycaemic seizure	-	-	-	-	-	1 (0.3)	1 (0.2)

### Disproportionality analyses

**Fig. 1:**
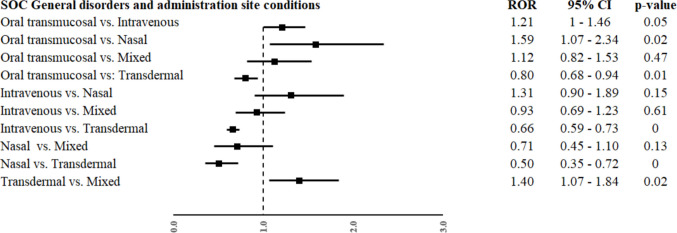
Reporting Odds Ratio (ROR) of System Organ Class (SOC) “General disorders and administration site conditions” comparing different routes of fentanyl administration. RORs and their 95% confidence intervals (CI) were calculated for the SOC and presented both graphically as Forrest Plot (black square with line) and numerically. A p-value greater than 0.05 was used for statistical significance

The disproportionality analysis on the SOC “general disorders and administration site conditions” (Fig. 1) showed a statistically significant lower disproportionate reporting for the routes oral transmucosal (ROR 0.80; 95% CI, 0.68–0.94), nasal (ROR 0.50; 95% CI, 0.35–0.72), and intravenous (ROR 0.66; 95% CI, 0.59–0.73) compared to the transdermal routes, while a higher disproportionate reporting fororal transmucosal than nasal (ROR 1.59; 95% CI, 1.07–2.34).

**Fig. 2:**
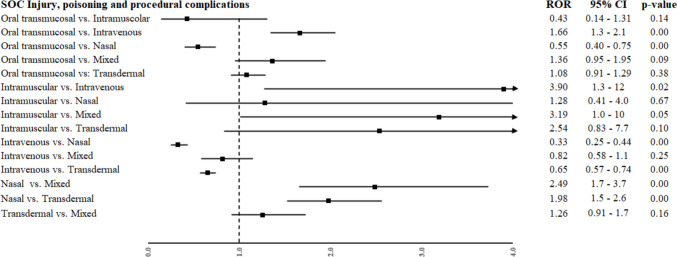
Reporting Odds Ratio (ROR) of System Organ Class (SOC) “Injury, poisoning and procedural complications” comparing different routes of fentanyl administration. RORs and their 95% confidence intervals (CI) were calculated for the SOC and presented both graphically as Forrest Plot (black square with line) and numerically. A p-value greater than 0.05 was used for statistical significance

For the SOC “injury poisoning and procedural complications” (Fig. 2), the nasal route of administration was associated with a higher disproportionate reporting for most comparisons (nasal vs. transdermal: ROR 1.98; 95% CI, 1.5–2.6; intravenous vs. nasal: ROR 0.33; 95% CI, 0.24–0.44; oral transmucosal vs. nasal: ROR 0.55; 95% CI, 0.40–0.75).

**Fig. 3:**
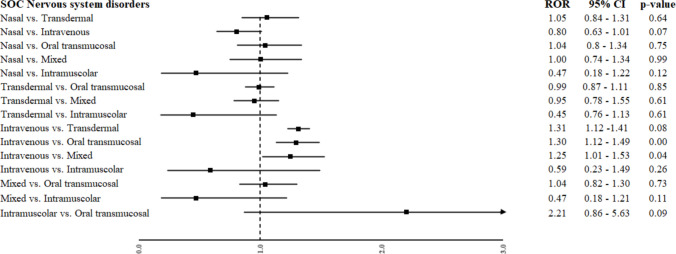
Reporting Odds Ratio (ROR) of System Organ Class (SOC) “nervous system disorders” comparing different routes of fentanyl administration. RORs and their 95% confidence intervals (CI) were calculated for the SOC and presented both graphically as Forrest plot (black square with line) and numerically. A p-value greater than 0.05 was used for statistical significance

The disproportionate reporting of SOC “nervous system disorders” was higher with the intravenous route compared to the transdermal (ROR 1.31; 95% CI, 1.12–1.41), oral transmucosal (ROR 1.30; 95% CI,1.12–1.49), and mixed (ROR 1.25; 95% CI, 1.01–1.53) routes of administration (Fig. 3).

**Fig. 4:**
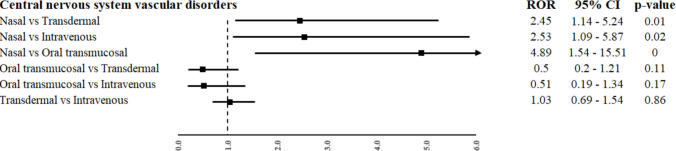
Reporting Odds ratio (ROR) of High-Level Group Term (HLGT) “central nervous system vascular disorders” comparing different routes of fentanyl administration. RORs and their 95% confidence intervals (CI) were calculated for the HLGT and presented both graphically as Forrest plot (black square with line) and numerically. A p-value greater than 0.05 was used for statistical significance

For the analysis on the LLT “brain lesions”, it was performed only the comparison intravenous vs. transdermal route of administration (that had more than 3 AEs) not showing a statistically significant difference (ROR, 1.61; 95% CI 0.75—3.45). Regarding the HLGT “central nervous system vascular disorders”, higher disproportionate reporting was observed for the nasal route compared to the transdermal (ROR 2.45; 95% CI, 1.14–5.24), intravenous (ROR 2.53; 95% CI, 1.09–5.87) and oral transmucosal (ROR 4.89; 95% CI 1.54–15.51) routes (Fig. 4).

**Fig. 5:**
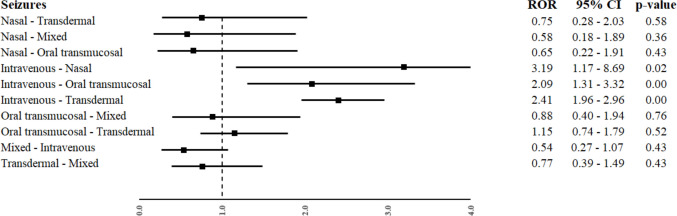
Reporting Odds ratio (ROR) of High-Level Group Term (HLGT) “seizures” comparing different routes of fentanyl administration. RORs and their 95% confidence intervals (CI) were calculated for the HLGT and presented both graphically as Forrest plot (black square with line) and numerically. A p-value greater than 0.05 was used for statistical significance

The HLGT “seizures” revealed a higher disproportionate reporting associated with the intravenous route compared to the nasal (ROR, 3.19; 95% CI, 1.17–8.69), oral transmucosal (ROR 2.09; 95% CI, 1.31- 3.32), and transdermal (ROR 2.41; 95% CI,1.96–2.96) routes (Fig. 5).

The stratified analyses by sex showed for the intravenous route that males reported more frequently AEs belonging to the SOC nervous system disorders (ROR 1.24, 95% CI 1.08–1.43) and the HLGT central nervous system vascular disorders (ROR 2.26, 95% CI 1.02–5.00) compared to females. However, there was no statistically significant difference in all other analyses stratified by sex (Supplementary Fig. 2).

### Discussion

The results of this analysis provide a picture of the safety profile of fentanyl administered through different routes of administration, with particular attention to brain lesions and AEs associated with brain lesions.

Firstly, it is important to note that most reports were related to transdermal administration, which accounted for 80.9% of reported cases. This is in accordance with the wide use in clinical practice of transdermal formulations. The transdermal route, indeed, provides a useful approach to deliver drugs through the skin, bypassing the gastrointestinal tract and avoiding first-pass metabolism. This method is particularly effective for drugs that are poorly absorbed orally or extensively metabolized (Khan and Sharman 2024). Transdermal fentanyl was associated with a positive signal of events belonging to the SOC “general disorders and administration site conditions”, probably due to the topical characteristic of this route of administration that can cause local adverse reactions including irritant and allergic reactions (Tanner and Marks 2008).

The nasal route of administration was instead associated with a positive signal for events belonging to the SOC “injury poisoning and procedural complications”. In this context, we need to reflect that events such as overdose or procedural complications belong to this SOC, supposing a higher frequency of medication errors with this route of administration. Nasal sprays can indeed face several technique errors during their application, potentially leading to dosing errors, especially when patients are poorly educated (Al-Taie 2025). Healthcare professionals are advised to provide proper instruction to patients on the correct use of nasal formulations.

The high number of central nervous system disorders observed with fentanyl is in accordance with its mechanism of action and its main site of therapeutic action represented by the central nervous system. Fentanyl is an opioid agonist that acts primarily by interacting with μ-opioid receptors located in the brain, spinal cord, and smooth muscle (Mystakidou et al. 2011). The chronic use of fentanyl has been associated with the occurrence of neurotoxicity such as cognitive impairment, severe sedation, hallucinations, and delirium. Fentanyl can exert its neurotoxic effects through a direct and indirect action on other neurotransmitter systems, such as dopaminergic and glutamatergic, and by inducing oxidative stress, mitochondrial dysfunction, and apoptosis (Cunha-Oliveira et al. 2008). Histopathological features of neuronal degeneration and necrosis were observed with its chronic use and related to a decreased Bcl-2/Bax ratio and increased of pro-apoptosis proteins (Alzu’bi et al. 2024). Moreover, another mechanism that could contribute to the onset of brain lesion is the hypoxic-ischaemic damage caused by respiratory depression after the fentanyl use. This condition is known to cause brain damage, particularly in cases of overdose. Studies conducted on animal models have shown that the intravenous administration of high doses of fentanyl induces rapid and severe respiratory depression (Solis et al. 2017). Another possible explanation of brain lesions is polypharmacy. The U.S. Food and Drug Administration has explicitly warned that concomitant use of fentanyl with benzodiazepines or other CNS depressants can cause deep sedation, respiratory depression, coma and death. They recommend limiting such combinations to cases where there are no adequate therapeutic alternatives (Medicine NL of DailyMed n.d.). In humans, cases of brain injury were observed after inhalation, intravenous, transdermal, and oral administration of fentanyl (Foy et al. 2011; Voigt 2013; Solis et al. 2018; Eden et al. 2024). In our analysis on “brain lesion”, a total of 50 AEs were found, of which the 76% (N = 38) were related to transdermal administration. However, this higher reporting was not confirmed in the disproportionality analysis comparing the transdermal vs. intravenous fentanyl. This apparent discrepancy can also be explained by the higher use of transdermal fentanyl in clinical practice that can generate an overall higher number of reports, increasing the denominator of disproportionate estimates. However, due to the rarity of the event and the limited number of cases available, any difference observed between the different routes of administration must be interpreted with extreme caution, as they do not permit the drawing of causal conclusions.

From our research other routes of administration, such as the nasal and the intravenous, were found to be associated with an increased reporting of AEs representing potential cause/symptoms of brain lesions. In particular, the nasal route was associated with a positive signal for events included in the HLGT “central nervous system vascular disorders” compared to the transdermal, intravenous and oral transmucosal routes. On the other hand, the intravenous route showed a positive signal for events included in the HLGT “seizures” compared to the nasal and transdermal route. Furthermore, the intravenous route of administration showed a stronger positive signal for central nervous system disorders than the transdermal and oral transmucosal routes.

It has been observed that intranasal administration of drugs with low molecular weight and high lipophilicity results in blood concentrations similar to those obtained with intravenous administration (Mystakidou et al. 2011). Indeed, it is important to note that rapid absorption and the resulting high blood concentration may increase the risk of AEs associated with fentanyl. Studies have shown that intranasal and intravenous administration of fentanyl may result in higher blood concentrations than other routes of administration, which may increase the risk of AEs (Peng and Sandler 1999; Mystakidou et al. 2011). Pharmacokinetic studies have also shown that nasal formulations of fentanyl may produce a significantly higher maximum concentration (Cmax) in the blood than other formulations, such as buccal tablets. This increase in Cmax may further contribute to the risk of AEs, especially if the administered dose is not adequately monitored (Mystakidou et al. 2011). Moreover, another study compared different doses of fentanyl nasal pectin (FPNS)—100, 200, 400 and 800 μg—to oral transmucosal fentanyl citrate, showing that FPNS had a shorter mean time to maximum blood concentration (Tmax) and a higher maximum concentration (Cmax), up to 2.3 times higher than oral transmucosal fentanyl citrate (Fisher et al. 2010). The central effects of fentanyl, altogether with higher concentrations when administered nasally or intravenously, may explain the high reporting of central neurologic and cardiovascular AEs.

A possible explanation for the high incidence of central neurological events associated with intravenous and intranasal formulations is the presence of confounding factors related to the clinical setting. These routes of administration are often employed in acute, emergency or perioperative settings, where patients tend to have more severe clinical conditions and an inherently higher risk of neurological complications. Consequently, the increase in reports may reflect the clinical setting rather than a direct effect of the route of administration.

This study identified a greater number of reports from countries outside Europe. The EV can also collect serious safety reports coming from non-EEA (Zinzi et al. 2023), explaining this discrepancy and the different patterns of fentanyl international use. One example is the illicit use of fentanyl, which is more prevalent in the United States (US) than in Europe and contributing to a severe overdose crisis. The Centers for Disease Control and Prevention (CDC) reported a significant increase in fentanyl-related overdose deaths since 2013, with 71,238 deaths in the US in 2021 (Walter 2023).

In the analysis for concomitants, the most reported were analgesic and psycholeptics. This is probably associated with the therapeutic use for pain control and anaesthesia. Indeed, the association between fentanyl and psycholeptics is a technique known as “neuroleptoanalgesia” that allows a pain-free state and suppression of motor activity (2018).

### Strengths and limitations

This study has several strengths. Pharmacovigilance analyses are important for increasing information on the safety profiles of drugs in the everyday clinical context. To do this, databases such as EV containing real data are used to identify potential safety problems at an early stage. These databases enable rapid and inexpensive methods to highlight adverse events that might go undetected in pre-market clinical studies of drugs.

Although pharmacovigilance studies are essential for monitoring the safety of medicines, they have some important limitations. Major problems are under-reporting, incomplete or missing data. In particular, the possibility of finding incomplete data in all ICSRs in the EV database may negatively affect the evaluation of safety signals and is a major limitation of our research. Incomplete data can also impede the evaluation of confounding factors. Indeed, we cannot be sure if all information on concomitant medicines or diseases is reported in a pharmacovigilance case. This limitation can affect the interpretation of our results by showing possible false positive results. Moreover, the EV database as all pharmacovigilance databases may have an inappropriate coding problem difficult to control that can affect our results. Another limiting aspect of our study is related to the dynamic nature of pharmacovigilance databases, which can be affected by changes over time, such as the removal of some reports for various reasons. For this reason, we have clearly defined data extraction periods in our methods to ensure consistency and reliability of results. A further limitation is the possible co-presence of multiple routes of administration for a single patient. As indicated in the Methods section, such cases were categorised as “mixed”. However, given the heterogeneity of this group and the impossibility of accurately attributing an adverse event to a specific route, there is difficulties in interpreting comparisons. Results for the 'mixed' category should be interpreted with particular caution. The last limitation is that almost half of the AEs were reported with two or more suspected drugs. This may create the possibility of false positives results, as the presence of multiple suspected drugs makes difficult to determine the specific contribution of each drug. For data quality issues, pharmacovigilance studies are not the best tool to evaluate drug-drug interactions, but further research with different methodologies can be used to establish whether the observed events are exclusively attributable to fentanyl administration or to its co-administration with other suspected or concomitant drugs.

## Conclusions

In conclusion, this study provides first information on the safety profile of fentanyl administered through different routes of administration that can be useful for clinical practice and further investigated in other studies with different designs. This study highlights the importance of a correct patient education in using nasal formulations, and to pay attention to intravenous and nasal fentanyl administration that were associated with positive signal of potential symptoms or causes of brain lesions. These data should be considered in pain management and in the risk–benefit assessment of different fentanyl formulations. Further research is needed to confirm our findings and to optimize the use of different formulations of fentanyl for pain management.

## Supplementary Information

Below is the link to the electronic supplementary material.

ESM 1 (PDF 208 KB)

ESM 2 (DOCX 688 KB)

## Data Availability

The data that support the findings of this study are openly available in EudraVigilance (European database of suspected adverse drug reaction reports) at [https://www.adrreports.eu/](https://www.adrreports.eu).
