# Supplementary material for: Safety profile of fentanyl with different routes of administration: a disproportionality analysis using the EudraVigilance database
Source: Naunyn Schmiedebergs Arch Pharmacol. 2026 Feb 27;399(8):11923–37. doi: 10.1007/s00210-026-05145-8 (PMC13269385; doi:10.1007/s00210-026-05145-8)
Supplement: Supplementary file 1 — (PDF 208 KB) [file 210_2026_5145_MOESM1_ESM.pdf]

The READUS-PV checklist

Section and topic	Item #	Checklist item	Location where item is reported
**Title**			
	1a	*If disproportionality analyses are a prominent component of the published study, the study should be identified as a "disproportionality analysis". The type of data and name of the database(s) should be specified.*	No. We specified them in the sections 2.2. Data source and 2.6. Disproportionality Analysis.
	1b	*Report the name of adverse event(s) and/or drug(s) under study, when applicable.*	Yes.
**Introduction**			
Background	*2a*	*Describe the drug(s) and its utilization, the nature of the adverse event(s) under study and its frequency, and the existing knowledge on the drug-event combination.*	Yes.
	2b	*Specify the rationale for performing the analysis, e.g., as part of routine pharmacovigilance, to investigate an overall safety profile, or to assess a pre-specified hypothesis.*	Yes.
	2c	*Explain why ICSR databases and disproportionality analysis are suitable to fill the knowledge gap.*	Yes.
Objectives	*3*	*State specific objectives, identifying the adverse event(s), the drug(s), and the reference group, including any pre-specified hypothesis, if applicable.*	Yes.
**Methods**			
Study design	*4a*	*Identify the study (i.e., "disproportionality analysis") and the type of data used (e.g., "individual case safety reports").*	Yes. We specified them in the sections 2.2. Data source and 2.6. Disproportionality Analysis.
	4b	*Provide an outline of the entire study design, including primary and sensitivity analyses performed, and other designs such as case-by-case analysis or literature review.*	Yes. We specified them in the sections 2.3. Study outcomes and 2.6. Disproportionality Analysis.
Data description, access, and pre-processing	*5a*	*Specify the name of the database(s), the database(s) custodian, and the coverage. Specify the type/number of drugs included within the database and the thesaurus, taxonomies, or ontologies used for coding drugs and events.*	Yes. We specified them in the section 2.2. Data source.
	5b	*Specify the extraction dates and describe and justify all choices used for data pre-processing, including any data transformation or exclusion, if appropriate.*	Yes. We specified them in the section

			2.4. ICSRs selection.
Variables definition	6a	*Describe the study population, including any restriction.*	Yes. We specified them in the sections 2.4. ICSRs selection and 2.5. Descriptive Analysis.
	6b	*Describe the nature and the meaning of key variables assessed in the work.*	Yes. We specified them in the sections 2.3. Study outcomes and 2.5. Descriptive Analysis.
	6c	*Specify and justify any grouping of drugs or events. For drugs, specify and justify whether active ingredients/trade names/salts were considered and/or the selected role.*	Yes. We specified them in the sections 2.3. Study outcomes and 2.4. ICSRs selection.
	6d	*Describe any additional data source used, the type of data, and how they interact with ICSRs.*	No. We did not use any additional data source.
Statistical methods	7a	*Present any descriptive analysis performed, specifying variables investigated, statistical tests, and significance thresholds.*	Yes. We specified them in the section 2.5. Descriptive Analysis.
	7b	*Describe the measure(s) selected for the disproportionality analysis including any threshold used to identify signals of disproportionate reporting. Explain the reason for this choice if applicable.*	Yes. We specified them in the section 2.6. Disproportionality Analysis.
	7c	*Clearly describe any sensitivity analysis and any tool to control confounding, including any restriction, subgroup, stratification, adjustment, or interaction.*	Yes. We specified them in the section 2.6. Disproportionality Analysis.
	7d	*Specify the variables and methods used for the case-by-case analysis, including any algorithm or criteria used to assess causality, if performed.*	No.
	7e	*Specify any statistical methods used for other data sources.*	No.

**Results**			
Participants	*8a*	*Specify the number of individual case safety reports included at each stage, including reasons for exclusion.*	Yes. We specified them in the section 3.1. Descriptive analysis of cases and in Figure 1.
	8b	*Provide key demographic and clinical characteristics of cases, if possible comparing cases with any appropriate reference group.*	Yes. We specified them in the section 3.1. Descriptive analysis.
Disproportionality analysis	*9*	*Present all results including confidence intervals. Present also results of sensitivity analyses, if performed.*	Yes. We specified them in the section 3.2 Disproportionality analyses.
Case-by-case analysis	*10*	*Present the case-by-case analysis of key variables. Present the causality assessment, if applicable.*	No.
**Discussion**			
Key results	*11*	*Discuss key results with reference to study objectives and contextualize them within the current literature and other consulted sources. Clearly discriminate between expected reactions and emerging safety signals.*	Yes.
External validity	*12a*	*Discuss the external validity of the results to the general population.*	Yes.
	12b	*Discuss the potential relevance of results in clinical practice*	Yes.
	12c	*Propose further study designs if applicable*	Yes. We specified them in the section 5. Conclusions.
Limitations	*13*	*Present general limitations, making clear that disproportionality analysis alone cannot prove causation or measure incidence, and specific limitations, including confounding and reporting bias and efforts to mitigate them.*	Yes. We specified them in the section 4.2. Strengths and limitations.
**Declarations**			
	14a	*Provide the source of funding/sponsorship and the role of the funders/sponsors for the present study and for any original study on which the present article is based.*	No.
	14b	*Clearly identify potential commercial and intellectual conflicts of interest (e.g., link to any drug/event investigated, whether financial, legal action, or software used).*	Yes.
	14c	*Declare any institutional approval needed or granted in the investigation.*	No.
	14d	*Include a statement on data availability, code availability (including the version of the statistical software used), and protocol registration.*	Yes.
