# Supplementary material for: Safety profile of fentanyl with different routes of administration: a disproportionality analysis using the EudraVigilance database
Source: Naunyn Schmiedebergs Arch Pharmacol. 2026 Feb 27;399(8):11923–37. doi: 10.1007/s00210-026-05145-8 (PMC13269385; doi:10.1007/s00210-026-05145-8)
Supplement: Supplementary file 2 — (DOCX 688 KB) [file 210_2026_5145_MOESM2_ESM.docx]

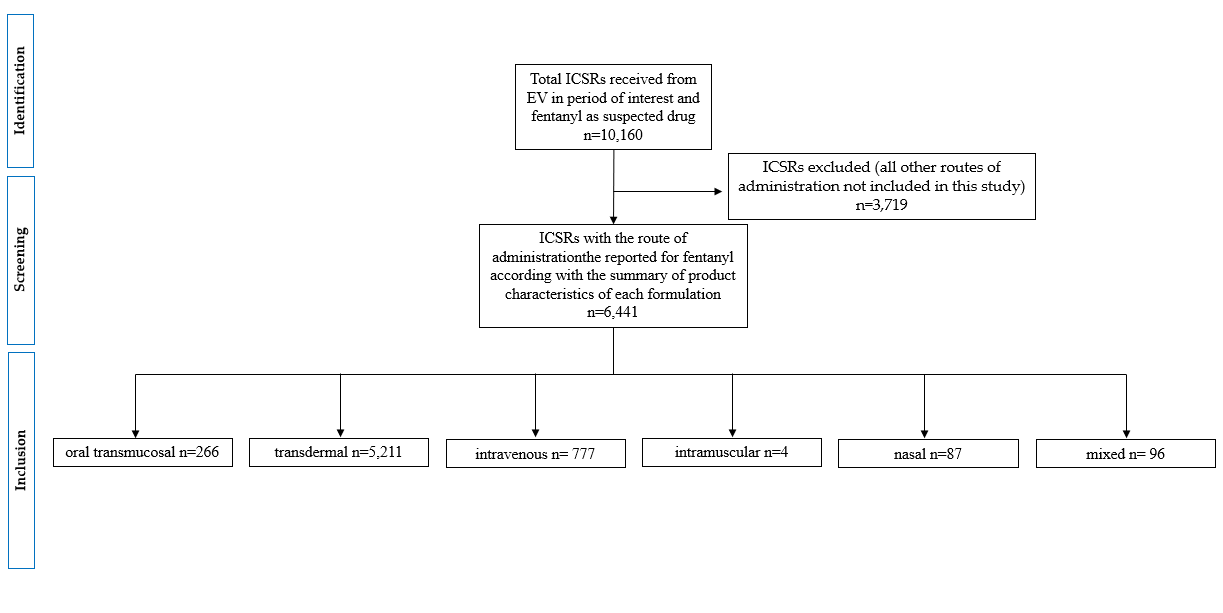


**Supplementary Figure 1**. Flowchart of the selection process of ICSRs from the EudraVigilance database.

**Supplementary Table 1.** Distribution of concomitant drugs classified by the second level of the Anatomical Therapeutic Chemical (ATC) classification system reported in the Individual Case Safety Reports (ICSRs) reported the different route of fentanyl administration as the suspected drug, retrieved from the EudraVigilance spontaneous reporting system as of 7 March 2024. Data are expressed as number and percentage (%).

|  |  | **Overall (N=19444)** |
| --- | --- | --- |
| **ATC** |  |  |
| A01 | Stomatological Preparations | 55 (0.3) |
| A02 | Drugs For Acid Related Disorders | 1041 (5.4) |
| A03 | Drugs For Functional Gastrointestinal Disorders | 299 (1.5) |
| A04 | Antiemetics And Antinauseants | 139 (0.7) |
| A05 | Bile And Liver Therapy | 21 (0.1) |
| A06 | Drugs For Constipation | 513 (2.6) |
| A07 | Antidiarrheals | 171 (0.9) |
| A08 | Antiobesity Preparations, Excl. Diet Products | 6 (0.0) |
| A09 | Digestives, incl. enzymes | 23 (0.1) |
| A10 | Drugs Used In Diabetes | 441 (2.3) |
| A11 | Vitamins | 429 (2.2) |
| A12 | Mineral Supplements | 340 (1.7) |
| A16 | Other Alimentary Tract And Metabolism Products | 23 (0.1) |
| B01 | Antithrombotic Agents | 392 (2.0) |
| B02 | Antihemorrhagics | 28 (0.1) |
| B03 | Antianemic Preparations | 203 (1.0) |
| B05 | Blood Substitutes And Perfusion Solutions | 253 (1.3) |
| C01 | Cardiac Therapy | 293 (1.5) |
| C02 | Antihypertensives | 106 (0.5) |
| C03 | Diuretics | 590 (3.0) |
| C04 | Peripheral Vasodilators | 12 (0.1) |
| C05 | Vasoprotectives | 3 (0.0) |
| C07 | Beta-Blocking Agents | 485 (2.5) |
| C08 | Calcium Channel Blockers | 332 (1.7) |
| C09 | Agents Acting On The Renin–Angiotensin System | 509 (2.6) |
| C10 | Lipid-Modifying Agents | 418 (2.1) |
| D01 | Antifungals For Dermatological Use | 42 (0.2) |
| D02 | Emollients And Protectives | 3 (0.0) |
| D03 | Preparations For Treatment Of Wounds And Ulcers | 1 (0.0) |
| D04 | Antipruritics, Incl. Antihistamines, Anesthetics, Etc. | 92 (0.5) |
| D05 | Antipsoriatics | 12 (0.1) |
| D07 | Corticosteroids, Dermatological Preparations | 56 (0.3) |
| D08 | Antiseptics And Disinfectants | 7 (0.0) |
| D09 | Medicated Dressings | 3 (0.0) |
| D10 | Anti-Acne Preparations | 3 (0.0) |
| D11 | Other Dermatological Preparations | 155 (0.8) |
| G01 | Gynecological Antiinfectives And Antiseptics | 20 (0.1) |
| G02 | Other Gynecologicals | 66 (0.3) |
| G03 | Sex Hormones And Modulators Of The Genital System | 177 (0.9) |
| G04 | Urologicals | 161 (0.8) |
| H01 | Pituitary And Hypothalamic Hormones And Analogues | 30 (0.2) |
| H02 | Corticosteroids For Systemic Use | 47 (0.2) |
| H03 | Thyroid Therapy | 294 (1.5) |
| H04 | Pancreatic Hormones | 2 (0.0) |
| H05 | Calcium Homeostasis | 26 (0.1) |
| J01 | Antibacterials For Systemic Use | 355 (1.8) |
| J02 | Antimycotics For Systemic Use | 16 (0.1) |
| J04 | Antimycobacterials | 10 (0.1) |
| J05 | Antivirals For Systemic Use | 35 (0.2) |
| J06 | Immune Sera And Immunoglobulins | 4 (0.0) |
| J07 | Vaccines | 2 (0.0) |
| L01 | Antineoplastic Agents | 243 (1.2) |
| L02 | Endocrine Therapy | 62 (0.3) |
| L03 | Immunostimulants | 16 (0.1) |
| L04 | Immunosuppressants | 96 (0.5) |
| M01 | Antiinflammatory And Antirheumatic Products, Non-Steroids | 350 (1.8) |
| M02 | Topical Products For Joint And Muscular Pain | 44 (0.2) |
| M03 | Muscle Relaxants | 506 (2.6) |
| M04 | Antigout Preparations | 70 (0.4) |
| M05 | Drugs For Treatment Of Bone Diseases | 75 (0.4) |
| M09 | Other Drugs For Disorders Of The Musculo-Skeletal System | 2 (0.0) |
| N01 | Anesthetics | 584 (3.0) |
| N02 | Analgesics | 2346 (12.1) |
| N03 | Antiepileptics | 1097 (5.6) |
| N04 | Anti-Parkinson Drugs | 107 (0.6) |
| N05 | Psycholeptics | 1697 (8.7) |
| N06 | Psychoanaleptics | 1364 (7.0) |
| N07 | Other Nervous System Drugs | 108 (0.6) |
| P01 | Antiprotozoals | 33 (0.2) |
| P02 | Anthelmintics | 1 (0.0) |
| P03 | Ectoparasiticides, Incl. Scabicides, Insecticides And Repellents | 8 (0.0) |
| R01 | Nasal Preparations | 118 (0.6) |
| R02 | Throat Preparations | 37 (0.2) |
| R03 | Drugs For Obstructive Airway Diseases | 374 (1.9) |
| R05 | Cough And Cold Preparations | 400 (2.1) |
| R06 | Antihistamines For Systemic Use | 236 (1.2) |
| R07 | Other Respiratory System Products | 4 (0.0) |
| S01 | Ophthalmologicals | 561 (2.9) |
| S02 | Otologicals | 5 (0.0) |
| S03 | Ophthalmological And Otological Preparations | 1 (0.0) |
| V03 | All Other Therapeutic Products | 126 (0.6) |
| V04 | Diagnostic Agents | 26 (0.1) |
| V08 | Contrast Media | 1 (0.0) |
| V09 | [Diagnostic Radiopharmaceuticals](https://atcddd.fhi.no/atc_ddd_index/?code=V09&showdescription=no) | 2 (0.0) |

**Supplementary Table 2** Descriptive analysis of preferred terms extracted from Individual Case Safety Reports (ICSRs) of different routes of fentanyl administration, retrieved from the EudraVigilance spontaneous reporting system up to 7 March 2024. Data are expressed as number and percentage (%).

|  | **Oral transmucosal (N=1345)** | **Intramuscular (N=18)** | **Intravenous (N=3779)** | **Nasal (N=390)** | **Other (N=511)** | **Transdermal (N=28928)** | **Overall (N=34971)** |
| --- | --- | --- | --- | --- | --- | --- | --- |
| **Preferred Terms** |  |  |  |  |  |  |  |
| Abdominal distension | 4 (0.3%) | - | 2 (0.1%) | 1 (0.3%) | - | 25 (0.1%) | 32 (0.1%) |
| Abdominal pain | 4 (0.3%) | - | 17 (0.4%) | - | 3 (0.6%) | 82 (0.3%) | 106 (0.3%) |
| Abdominal pain upper | 3 (0.2%) | - | 1 (0.0%) | - | 2 (0.4%) | 56 (0.2%) | 62 (0.2%) |
| Abnormal behaviour | 1 (0.1%) | - | 3 (0.1%) | - | 5 (1.0%) | 47 (0.2%) | 56 (0.2%) |
| Abnormal faeces | 1 (0.1%) | - | - | - | - | 2 (0.0%) | 3 (0.0%) |
| Abscess | 1 (0.1%) | - | - | - | - | - | 1 (0.0%) |
| Abscess rupture | 1 (0.1%) | - | - | - | - | - | 1 (0.0%) |
| Accidental exposure to product | 6 (0.4%) | - | 4 (0.1%) | - | - | 18 (0.1%) | 28 (0.1%) |
| Accidental exposure to product by child | 7 (0.5%) | - | - | - | - | 7 (0.0%) | 14 (0.0%) |
| Accidental overdose | 7 (0.5%) | - | 4 (0.1%) | 1 (0.3%) | - | 101 (0.3%) | 113 (0.3%) |
| Acute kidney injury | 1 (0.1%) | - | 7 (0.2%) | - | - | 45 (0.2%) | 53 (0.2%) |
| Acute resry failure | 1 (0.1%) | - | 2 (0.1%) | - | - | 11 (0.0%) | 14 (0.0%) |
| Administration site injury | 1 (0.1%) | - | - | - | - | - | 1 (0.0%) |
| Administration site pain | 1 (0.1%) | - | - | - | - | - | 1 (0.0%) |
| Adrenocortical insufficiency acute | 1 (0.1%) | - | - | - | - | 1 (0.0%) | 2 (0.0%) |
| Adverse event | 2 (0.1%) | - | 3 (0.1%) | - | - | 55 (0.2%) | 60 (0.2%) |
| Affect lability | 1 (0.1%) | - | 2 (0.1%) | - | - | 7 (0.0%) | 10 (0.0%) |
| Ageusia | 4 (0.3%) | - | 2 (0.1%) | - | - | 4 (0.0%) | 10 (0.0%) |
| Aggression | 3 (0.2%) | - | 2 (0.1%) | - | 4 (0.8%) | 36 (0.1%) | 45 (0.1%) |
| Agitation | 1 (0.1%) | - | 39 (1.0%) | 6 (1.5%) | 5 (1.0%) | 104 (0.4%) | 155 (0.4%) |
| Akathisia | 1 (0.1%) | - | 5 (0.1%) | - | - | 23 (0.1%) | 29 (0.1%) |
| Allodynia | 3 (0.2%) | - | 2 (0.1%) | - | 1 (0.2%) | 9 (0.0%) | 15 (0.0%) |
| Altered state of consciousness | 15 (1.1%) | - | 22 (0.6%) | 1 (0.3%) | 3 (0.6%) | 120 (0.4%) | 161 (0.5%) |
| Amnesia | 7 (0.5%) | - | 5 (0.1%) | 1 (0.3%) | 1 (0.2%) | 140 (0.5%) | 154 (0.4%) |
| Anaemia | 3 (0.2%) | - | 15 (0.4%) | - | 1 (0.2%) | 39 (0.1%) | 58 (0.2%) |
| Anaemia of chronic disease | 1 (0.1%) | - | - | - | - | - | 1 (0.0%) |
| Anal erosion | 1 (0.1%) | - | - | - | - | - | 1 (0.0%) |
| Anal incontinence | 1 (0.1%) | - | 1 (0.0%) | - | - | 11 (0.0%) | 13 (0.0%) |
| Anal ulcer | 1 (0.1%) | - | - | - | - | - | 1 (0.0%) |
| Androgen deficiency | 1 (0.1%) | - | - | - | - | 1 (0.0%) | 2 (0.0%) |
| Anticonvulsant drug level decreased | 1 (0.1%) | - | - | - | - | - | 1 (0.0%) |
| Anxiety | 10 (0.7%) | - | 10 (0.3%) | 2 (0.5%) | 4 (0.8%) | 210 (0.7%) | 236 (0.7%) |
| Apnoea | 2 (0.1%) | - | 25 (0.7%) | 3 (0.8%) | 1 (0.2%) | 42 (0.1%) | 73 (0.2%) |
| Appendix disorder | 1 (0.1%) | - | - | - | - | - | 1 (0.0%) |
| Appetite disorder | 1 (0.1%) | - | - | - | - | 1 (0.0%) | 2 (0.0%) |
| Application site irritation | 1 (0.1%) | - | - | - | - | 27 (0.1%) | 28 (0.1%) |
| Application site vesicles | 1 (0.1%) | - | - | - | - | 14 (0.0%) | 15 (0.0%) |
| Arteriosclerosis | 1 (0.1%) | - | - | - | - | 4 (0.0%) | 5 (0.0%) |
| Arthralgia | 2 (0.1%) | - | 3 (0.1%) | - | - | 65 (0.2%) | 70 (0.2%) |
| Asthenia | 5 (0.4%) | 1 (5.6%) | 10 (0.3%) | 2 (0.5%) | 2 (0.4%) | 216 (0.7%) | 236 (0.7%) |
| Ataxia | 1 (0.1%) | - | 7 (0.2%) | - | - | 20 (0.1%) | 28 (0.1%) |
| Back pain | 5 (0.4%) | - | 11 (0.3%) | - | 2 (0.4%) | 96 (0.3%) | 114 (0.3%) |
| Balance disorder | 2 (0.1%) | - | - | 1 (0.3%) | - | 69 (0.2%) | 72 (0.2%) |
| Bedridden | 2 (0.1%) | - | 1 (0.0%) | - | - | 26 (0.1%) | 29 (0.1%) |
| Blood immunoglobulin M decreased | 1 (0.1%) | - | - | - | - | - | 1 (0.0%) |
| Blood potassium decreased | 1 (0.1%) | - | - | - | 1 (0.2%) | 16 (0.1%) | 18 (0.1%) |
| Blood pressure decreased | 2 (0.1%) | - | 17 (0.4%) | 2 (0.5%) | - | 40 (0.1%) | 61 (0.2%) |
| Bone disorder | 2 (0.1%) | - | - | - | - | 5 (0.0%) | 7 (0.0%) |
| Bone pain | 1 (0.1%) | - | 1 (0.0%) | - | - | 8 (0.0%) | 10 (0.0%) |
| Bradycardia | 1 (0.1%) | - | 28 (0.7%) | - | 1 (0.2%) | 43 (0.1%) | 73 (0.2%) |
| Bradykinesia | 1 (0.1%) | - | 2 (0.1%) | - | - | 8 (0.0%) | 11 (0.0%) |
| Bradyphrenia | 2 (0.1%) | - | - | - | - | 11 (0.0%) | 13 (0.0%) |
| Bradypnoea | 1 (0.1%) | - | 4 (0.1%) | 4 (1.0%) | 2 (0.4%) | 46 (0.2%) | 57 (0.2%) |
| fog | 1 (0.1%) | - | - | - | - | 15 (0.1%) | 16 (0.0%) |
| Brain injury | 1 (0.1%) | - | 8 (0.2%) | 1 (0.3%) | 1 (0.2%) | 36 (0.1%) | 47 (0.1%) |
| Brain neoplasm | 1 (0.1%) | - | - | - | - | 5 (0.0%) | 6 (0.0%) |
| Brain oedema | 1 (0.1%) | - | 6 (0.2%) | 1 (0.3%) | - | 30 (0.1%) | 38 (0.1%) |
| Breakthrough pain | 3 (0.2%) | - | - | 1 (0.3%) | 1 (0.2%) | 36 (0.1%) | 41 (0.1%) |
| Breast cancer | 6 (0.4%) | - | - | - | - | 14 (0.0%) | 20 (0.1%) |
| Burning sensation | 1 (0.1%) | - | 3 (0.1%) | 1 (0.3%) | - | 43 (0.1%) | 48 (0.1%) |
| Burning sensation mucosal | 1 (0.1%) | - | - | - | - | - | 1 (0.0%) |
| Cachexia | 2 (0.1%) | - | - | - | 1 (0.2%) | 8 (0.0%) | 11 (0.0%) |
| Cardiac disorder | 1 (0.1%) | - | - | - | 1 (0.2%) | 19 (0.1%) | 21 (0.1%) |
| Cardio-respiratory arrest | 1 (0.1%) | - | 4 (0.1%) | - | - | 19 (0.1%) | 24 (0.1%) |
| Central nervous system infection | 1 (0.1%) | - | - | - | - | - | 1 (0.0%) |
| Central nervous system lesion | 1 (0.1%) | - | - | - | - | 2 (0.0%) | 3 (0.0%) |
| Cerebrovascular accident | 3 (0.2%) | - | 2 (0.1%) | 3 (0.8%) | 2 (0.4%) | 120 (0.4%) | 130 (0.4%) |
| Cervical vertebral fracture | 1 (0.1%) | - | - | - | - | 2 (0.0%) | 3 (0.0%) |
| Cervix disorder | 1 (0.1%) | - | - | - | - | - | 1 (0.0%) |
| Chest discomfort | 2 (0.1%) | - | 2 (0.1%) | - | 1 (0.2%) | 26 (0.1%) | 31 (0.1%) |
| Chills | 5 (0.4%) | - | 21 (0.6%) | - | 1 (0.2%) | 81 (0.3%) | 108 (0.3%) |
| Chronic kidney disease | 1 (0.1%) | - | - | - | - | 6 (0.0%) | 7 (0.0%) |
| Circumstance or information capable of leading to medication error | 1 (0.1%) | - | - | - | - | 1 (0.0%) | 2 (0.0%) |
| Cold sweat | 2 (0.1%) | - | - | - | - | 51 (0.2%) | 53 (0.2%) |
| Colon cancer | 1 (0.1%) | - | - | - | 1 (0.2%) | 11 (0.0%) | 13 (0.0%) |
| Colorectal cancer | 5 (0.4%) | - | - | - | - | - | 5 (0.0%) |
| Coma | 8 (0.6%) | 2 (11.1%) | 39 (1.0%) | 13 (3.3%) | 9 (1.8%) | 298 (1.0%) | 369 (1.1%) |
| Condition aggravated | 2 (0.1%) | - | 11 (0.3%) | - | 4 (0.8%) | 43 (0.1%) | 60 (0.2%) |
| Confusional state | 14 (1.0%) | 1 (5.6%) | 18 (0.5%) | 3 (0.8%) | 5 (1.0%) | 373 (1.3%) | 414 (1.2%) |
| Constipation | 23 (1.7%) | - | 9 (0.2%) | 2 (0.5%) | 2 (0.4%) | 214 (0.7%) | 250 (0.7%) |
| Contusion | 1 (0.1%) | - | 2 (0.1%) | - | - | 28 (0.1%) | 31 (0.1%) |
| Cyanosis | 1 (0.1%) | 1 (5.6%) | 12 (0.3%) | 6 (1.5%) | 2 (0.4%) | 34 (0.1%) | 56 (0.2%) |
| Cyst | 1 (0.1%) | - | 1 (0.0%) | - | - | 3 (0.0%) | 5 (0.0%) |
| Death | 13 (1.0%) | - | 5 (0.1%) | - | 3 (0.6%) | 98 (0.3%) | 119 (0.3%) |
| Decreased activity | 1 (0.1%) | - | - | - | 1 (0.2%) | 5 (0.0%) | 7 (0.0%) |
| Decreased appetite | 11 (0.8%) | - | 8 (0.2%) | 2 (0.5%) | 2 (0.4%) | 194 (0.7%) | 217 (0.6%) |
| Decubitus ulcer | 2 (0.1%) | - | 3 (0.1%) | - | - | 9 (0.0%) | 14 (0.0%) |
| Dehydration | 8 (0.6%) | - | 3 (0.1%) | - | - | 91 (0.3%) | 102 (0.3%) |
| Delirium | 6 (0.4%) | - | 20 (0.5%) | - | 11 (2.2%) | 95 (0.3%) | 132 (0.4%) |
| Dental caries | 5 (0.4%) | - | - | - | 1 (0.2%) | 4 (0.0%) | 10 (0.0%) |
| Dependence | 4 (0.3%) | - | - | - | 1 (0.2%) | 17 (0.1%) | 22 (0.1%) |
| Depressed level of consciousness | 17 (1.3%) | 1 (5.6%) | 63 (1.7%) | 3 (0.8%) | 9 (1.8%) | 308 (1.1%) | 401 (1.1%) |
| Depressed mood | 1 (0.1%) | - | 3 (0.1%) | - | 2 (0.4%) | 25 (0.1%) | 31 (0.1%) |
| Depression | 4 (0.3%) | - | 5 (0.1%) | - | 3 (0.6%) | 172 (0.6%) | 184 (0.5%) |
| Dermatitis allergic | 1 (0.1%) | - | - | - | - | 3 (0.0%) | 4 (0.0%) |
| Diabetes mellitus | 2 (0.1%) | - | 2 (0.1%) | - | 1 (0.2%) | 23 (0.1%) | 28 (0.1%) |
| Diarrhoea | 7 (0.5%) | - | 7 (0.2%) | 1 (0.3%) | 2 (0.4%) | 154 (0.5%) | 171 (0.5%) |
| Discomfort | 2 (0.1%) | - | 3 (0.1%) | - | 1 (0.2%) | 22 (0.1%) | 28 (0.1%) |
| Disease progression | 4 (0.3%) | - | 1 (0.0%) | - | 1 (0.2%) | 26 (0.1%) | 32 (0.1%) |
| Disorientation | 6 (0.4%) | - | 11 (0.3%) | 1 (0.3%) | 3 (0.6%) | 114 (0.4%) | 135 (0.4%) |
| Dissociation | 1 (0.1%) | - | 1 (0.0%) | - | - | - | 2 (0.0%) |
| Disturbance in attention | 4 (0.3%) | - | 7 (0.2%) | 2 (0.5%) | 3 (0.6%) | 99 (0.3%) | 115 (0.3%) |
| Diverticular perforation | 1 (0.1%) | - | - | - | - | - | 1 (0.0%) |
| Diverticulitis | 1 (0.1%) | - | - | - | - | 6 (0.0%) | 7 (0.0%) |
| Dizziness | 20 (1.5%) | - | 25 (0.7%) | 4 (1.0%) | 4 (0.8%) | 660 (2.3%) | 713 (2.0%) |
| Drooling | 1 (0.1%) | - | - | - | 1 (0.2%) | 5 (0.0%) | 7 (0.0%) |
| Drug abuse | 9 (0.7%) | - | 16 (0.4%) | 15 (3.8%) | 8 (1.6%) | 77 (0.3%) | 125 (0.4%) |
| Drug dependence | 19 (1.4%) | - | 12 (0.3%) | 6 (1.5%) | 12 (2.3%) | 120 (0.4%) | 169 (0.5%) |
| Drug detoxification | 1 (0.1%) | - | 1 (0.0%) | - | - | 5 (0.0%) | 7 (0.0%) |
| Drug diversion | 3 (0.2%) | - | 3 (0.1%) | 2 (0.5%) | - | 23 (0.1%) | 31 (0.1%) |
| Drug dose titration not performed | 1 (0.1%) | - | - | - | 1 (0.2%) | 1 (0.0%) | 3 (0.0%) |
| Drug ineffective | 12 (0.9%) | - | 27 (0.7%) | 2 (0.5%) | 4 (0.8%) | 390 (1.3%) | 435 (1.2%) |
| Drug ineffective for unapproved indication | 1 (0.1%) | - | 1 (0.0%) | 1 (0.3%) | - | 4 (0.0%) | 7 (0.0%) |
| Drug interaction | 3 (0.2%) | - | 83 (2.2%) | 3 (0.8%) | 5 (1.0%) | 195 (0.7%) | 289 (0.8%) |
| Drug screen positive | 1 (0.1%) | - | - | - | - | 2 (0.0%) | 3 (0.0%) |
| Drug tolerance | 1 (0.1%) | - | 6 (0.2%) | 1 (0.3%) | 1 (0.2%) | 37 (0.1%) | 46 (0.1%) |
| Drug withdrawal convulsions | 1 (0.1%) | - | 1 (0.0%) | - | - | 13 (0.0%) | 15 (0.0%) |
| Drug withdrawal syndrome | 10 (0.7%) | - | 21 (0.6%) | 3 (0.8%) | 3 (0.6%) | 108 (0.4%) | 145 (0.4%) |
| Dry mouth | 5 (0.4%) | - | 2 (0.1%) | - | - | 67 (0.2%) | 74 (0.2%) |
| Dysaesthesia | 1 (0.1%) | - | - | - | - | 5 (0.0%) | 6 (0.0%) |
| Dysarthria | 3 (0.2%) | - | 4 (0.1%) | 2 (0.5%) | 1 (0.2%) | 94 (0.3%) | 104 (0.3%) |
| Dysgeusia | 2 (0.1%) | - | 1 (0.0%) | - | - | 17 (0.1%) | 20 (0.1%) |
| Dysphagia | 2 (0.1%) | - | 1 (0.0%) | - | 2 (0.4%) | 44 (0.2%) | 49 (0.1%) |
| Dyspnoea | 9 (0.7%) | 2 (11.1%) | 31 (0.8%) | 1 (0.3%) | 3 (0.6%) | 303 (1.0%) | 349 (1.0%) |
| Dystonia | 1 (0.1%) | 1 (5.6%) | 16 (0.4%) | 1 (0.3%) | 1 (0.2%) | 3 (0.0%) | 23 (0.1%) |
| Ear discomfort | 1 (0.1%) | - | - | - | - | - | 1 (0.0%) |
| Eating disorder | 1 (0.1%) | - | 2 (0.1%) | - | - | 30 (0.1%) | 33 (0.1%) |
| ECG signs of myocardial ischaemia | 1 (0.1%) | - | - | - | - | 1 (0.0%) | 2 (0.0%) |
| Electrocardiogram abnormal | 1 (0.1%) | - | - | - | - | 4 (0.0%) | 5 (0.0%) |
| Electrocardiogram QT prolonged | 1 (0.1%) | - | 3 (0.1%) | - | 2 (0.4%) | 4 (0.0%) | 10 (0.0%) |
| Electrocardiogram T wave abnormal | 1 (0.1%) | - | - | - | 1 (0.2%) | - | 2 (0.0%) |
| Electrolyte imbalance | 1 (0.1%) | - | 1 (0.0%) | - | - | 2 (0.0%) | 4 (0.0%) |
| Emotional distress | 1 (0.1%) | - | 5 (0.1%) | - | 1 (0.2%) | 12 (0.0%) | 19 (0.1%) |
| Emotional poverty | 1 (0.1%) | - | - | - | - | 1 (0.0%) | 2 (0.0%) |
| Enamel anomaly | 1 (0.1%) | - | - | - | - | - | 1 (0.0%) |
| Encephalopathy | 2 (0.1%) | - | 9 (0.2%) | - | 2 (0.4%) | 32 (0.1%) | 45 (0.1%) |
| Erectile dysfunction | 1 (0.1%) | - | - | - | - | 3 (0.0%) | 4 (0.0%) |
| Euphoric mood | 1 (0.1%) | - | 4 (0.1%) | 1 (0.3%) | - | 27 (0.1%) | 33 (0.1%) |
| Exposure during pregnancy | 1 (0.1%) | - | 14 (0.4%) | - | - | 9 (0.0%) | 24 (0.1%) |
| Extra dose administered | 1 (0.1%) | - | - | 1 (0.3%) | - | 8 (0.0%) | 10 (0.0%) |
| Eye swelling | 1 (0.1%) | - | - | - | - | 3 (0.0%) | 4 (0.0%) |
| Face injury | 2 (0.1%) | - | 1 (0.0%) | - | - | 3 (0.0%) | 6 (0.0%) |
| Face oedema | 1 (0.1%) | - | 1 (0.0%) | - | - | 2 (0.0%) | 4 (0.0%) |
| Facial paralysis | 1 (0.1%) | - | 2 (0.1%) | 1 (0.3%) | - | 6 (0.0%) | 10 (0.0%) |
| Faecal volume decreased | 1 (0.1%) | - | - | - | - | - | 1 (0.0%) |
| Faecaloma | 1 (0.1%) | - | - | - | - | 10 (0.0%) | 11 (0.0%) |
| Fall | 5 (0.4%) | - | 6 (0.2%) | 1 (0.3%) | 1 (0.2%) | 263 (0.9%) | 276 (0.8%) |
| Fatigue | 9 (0.7%) | - | 7 (0.2%) | 2 (0.5%) | 3 (0.6%) | 214 (0.7%) | 235 (0.7%) |
| Feeding disorder | 2 (0.1%) | - | 1 (0.0%) | - | - | 9 (0.0%) | 12 (0.0%) |
| Feeling abnormal | 3 (0.2%) | - | 3 (0.1%) | 1 (0.3%) | - | 170 (0.6%) | 177 (0.5%) |
| Feeling jittery | 2 (0.1%) | - | 3 (0.1%) | - | - | 11 (0.0%) | 16 (0.0%) |
| Flight of ideas | 1 (0.1%) | - | - | - | - | - | 1 (0.0%) |
| Flushing | 2 (0.1%) | - | 7 (0.2%) | - | - | 10 (0.0%) | 19 (0.1%) |
| Foetal hypokinesia | 1 (0.1%) | - | - | - | - | - | 1 (0.0%) |
| Formication | 2 (0.1%) | - | - | - | - | 40 (0.1%) | 42 (0.1%) |
| Fungal infection | 1 (0.1%) | - | 1 (0.0%) | - | - | 4 (0.0%) | 6 (0.0%) |
| Gait disturbance | 3 (0.2%) | - | 2 (0.1%) | 1 (0.3%) | 1 (0.2%) | 102 (0.4%) | 109 (0.3%) |
| Gait inability | 1 (0.1%) | - | 2 (0.1%) | - | - | 36 (0.1%) | 39 (0.1%) |
| Gallbladder disorder | 1 (0.1%) | - | - | - | - | 4 (0.0%) | 5 (0.0%) |
| Gastric cancer | 3 (0.2%) | - | - | - | 1 (0.2%) | 7 (0.0%) | 11 (0.0%) |
| Gastric haemorrhage | 1 (0.1%) | - | - | - | - | 2 (0.0%) | 3 (0.0%) |
| Gastric ulcer | 1 (0.1%) | - | - | - | - | 6 (0.0%) | 7 (0.0%) |
| Gastritis | 2 (0.1%) | - | 1 (0.0%) | - | - | 8 (0.0%) | 11 (0.0%) |
| Gastrointestinal disorder | 1 (0.1%) | - | 1 (0.0%) | - | - | 28 (0.1%) | 30 (0.1%) |
| Gastrointestinal haemorrhage | 2 (0.1%) | - | 4 (0.1%) | - | - | 6 (0.0%) | 12 (0.0%) |
| Gastrointestinal stromal tumour | 1 (0.1%) | - | - | - | - | 1 (0.0%) | 2 (0.0%) |
| Gastrooesophageal reflux disease | 1 (0.1%) | - | - | - | - | 13 (0.0%) | 14 (0.0%) |
| Gaze palsy | 1 (0.1%) | - | 4 (0.1%) | - | - | - | 5 (0.0%) |
| General physical health deterioration | 2 (0.1%) | - | 4 (0.1%) | 3 (0.8%) | - | 46 (0.2%) | 55 (0.2%) |
| Generalised tonic-clonic seizure | 2 (0.1%) | - | 25 (0.7%) | - | - | 28 (0.1%) | 55 (0.2%) |
| Gingival disorder | 1 (0.1%) | - | - | - | - | - | 1 (0.0%) |
| Gingival pain | 1 (0.1%) | - | - | - | - | - | 1 (0.0%) |
| Gingival recession | 1 (0.1%) | - | - | - | - | - | 1 (0.0%) |
| Gingivitis | 1 (0.1%) | - | - | - | - | - | 1 (0.0%) |
| Glossitis | 1 (0.1%) | - | - | - | - | - | 1 (0.0%) |
| Gun shot wound | 1 (0.1%) | - | - | - | - | 1 (0.0%) | 2 (0.0%) |
| Gynaecomastia | 1 (0.1%) | - | - | - | - | - | 1 (0.0%) |
| Haematemesis | 1 (0.1%) | - | 1 (0.0%) | 1 (0.3%) | - | 10 (0.0%) | 13 (0.0%) |
| Haemorrhoids | 1 (0.1%) | - | - | - | - | 9 (0.0%) | 10 (0.0%) |
| Hallucination | 4 (0.3%) | - | 9 (0.2%) | 2 (0.5%) | 3 (0.6%) | 167 (0.6%) | 185 (0.5%) |
| Hallucination, visual | 1 (0.1%) | - | 5 (0.1%) | - | - | 24 (0.1%) | 30 (0.1%) |
| Head and neck cancer | 2 (0.1%) | - | - | - | - | 1 (0.0%) | 3 (0.0%) |
| Headache | 9 (0.7%) | - | 38 (1.0%) | 5 (1.3%) | 1 (0.2%) | 341 (1.2%) | 394 (1.1%) |
| Heart rate decreased | 1 (0.1%) | - | 5 (0.1%) | - | - | 17 (0.1%) | 23 (0.1%) |
| Heart rate increased | 2 (0.1%) | - | 14 (0.4%) | - | 1 (0.2%) | 48 (0.2%) | 65 (0.2%) |
| Helplessness | 1 (0.1%) | - | - | - | - | 1 (0.0%) | 2 (0.0%) |
| Hepatic cytolysis | 1 (0.1%) | - | - | - | - | 5 (0.0%) | 6 (0.0%) |
| Hepatic encephalopathy | 2 (0.1%) | - | 2 (0.1%) | - | - | 11 (0.0%) | 15 (0.0%) |
| Hepatic enzyme abnormal | 1 (0.1%) | - | 1 (0.0%) | - | - | 2 (0.0%) | 4 (0.0%) |
| Hepatic failure | 1 (0.1%) | - | 5 (0.1%) | - | 2 (0.4%) | 9 (0.0%) | 17 (0.0%) |
| Hepatic steatosis | 1 (0.1%) | - | - | - | - | 1 (0.0%) | 2 (0.0%) |
| Hepatocellular carcinoma | 1 (0.1%) | - | - | - | - | - | 1 (0.0%) |
| Hospitalisation | 2 (0.1%) | - | - | - | - | 42 (0.1%) | 44 (0.1%) |
| Hot flush | 1 (0.1%) | - | 1 (0.0%) | - | 1 (0.2%) | 27 (0.1%) | 30 (0.1%) |
| Hyperaesthesia | 7 (0.5%) | - | 21 (0.6%) | 2 (0.5%) | 5 (1.0%) | 63 (0.2%) | 98 (0.3%) |
| Hyperhidrosis | 16 (1.2%) | - | 24 (0.6%) | 4 (1.0%) | - | 369 (1.3%) | 413 (1.2%) |
| Hyperkalaemia | 1 (0.1%) | - | 2 (0.1%) | - | - | 12 (0.0%) | 15 (0.0%) |
| Hyperresponsive to stimuli | 1 (0.1%) | - | - | - | - | - | 1 (0.0%) |
| Hypertension | 3 (0.2%) | - | 26 (0.7%) | 1 (0.3%) | 1 (0.2%) | 109 (0.4%) | 140 (0.4%) |
| Hypertonia | 1 (0.1%) | - | 9 (0.2%) | - | - | 2 (0.0%) | 12 (0.0%) |
| Hyperventilation | 2 (0.1%) | - | 3 (0.1%) | - | 1 (0.2%) | 15 (0.1%) | 21 (0.1%) |
| Hypoaesthesia | 3 (0.2%) | - | 9 (0.2%) | 2 (0.5%) | 1 (0.2%) | 96 (0.3%) | 111 (0.3%) |
| Hypoaesthesia oral | 1 (0.1%) | - | 2 (0.1%) | - | - | 6 (0.0%) | 9 (0.0%) |
| Hypokalaemia | 1 (0.1%) | - | 2 (0.1%) | - | 1 (0.2%) | 21 (0.1%) | 25 (0.1%) |
| Hypokinesia | 1 (0.1%) | - | 2 (0.1%) | - | - | 31 (0.1%) | 34 (0.1%) |
| Hyponatraemia | 2 (0.1%) | - | 1 (0.0%) | - | - | 18 (0.1%) | 21 (0.1%) |
| Hypophagia | 1 (0.1%) | - | - | - | - | 7 (0.0%) | 8 (0.0%) |
| Hypopnoea | 1 (0.1%) | - | 4 (0.1%) | - | 2 (0.4%) | 34 (0.1%) | 41 (0.1%) |
| Hyporeflexia | 1 (0.1%) | - | - | - | - | 3 (0.0%) | 4 (0.0%) |
| Hypotension | 8 (0.6%) | - | 48 (1.3%) | - | 2 (0.4%) | 134 (0.5%) | 192 (0.5%) |
| Hypothermia | 1 (0.1%) | - | - | - | - | 7 (0.0%) | 8 (0.0%) |
| Hypotonia | 1 (0.1%) | - | 6 (0.2%) | - | - | 12 (0.0%) | 19 (0.1%) |
| Hypovolaemia | 1 (0.1%) | - | - | - | - | - | 1 (0.0%) |
| Hypoxia | 2 (0.1%) | - | 11 (0.3%) | 1 (0.3%) | - | 28 (0.1%) | 42 (0.1%) |
| Ileus paralytic | 1 (0.1%) | - | - | - | - | 2 (0.0%) | 3 (0.0%) |
| Illness | 2 (0.1%) | - | 2 (0.1%) | - | - | 45 (0.2%) | 49 (0.1%) |
| Impulsive behaviour | 1 (0.1%) | - | - | - | - | 2 (0.0%) | 3 (0.0%) |
| Inappropriate schedule of product administration | 3 (0.2%) | - | - | 1 (0.3%) | - | 153 (0.5%) | 157 (0.4%) |
| Incision site complication | 1 (0.1%) | - | - | - | - | - | 1 (0.0%) |
| Incoherent | 4 (0.3%) | - | - | - | - | 50 (0.2%) | 54 (0.2%) |
| Incorrect dosage administered | 1 (0.1%) | - | 1 (0.0%) | 1 (0.3%) | - | 6 (0.0%) | 9 (0.0%) |
| Incorrect dose administered | 3 (0.2%) | - | 1 (0.0%) | 6 (1.5%) | 1 (0.2%) | 25 (0.1%) | 36 (0.1%) |
| Incorrect route of product administration | 2 (0.1%) | 1 (5.6%) | 14 (0.4%) | 3 (0.8%) | 3 (0.6%) | 12 (0.0%) | 35 (0.1%) |
| Infection | 2 (0.1%) | - | 4 (0.1%) | - | - | 31 (0.1%) | 37 (0.1%) |
| Inflammation of wound | 1 (0.1%) | - | - | - | - | - | 1 (0.0%) |
| Influenza | 2 (0.1%) | - | - | - | - | 8 (0.0%) | 10 (0.0%) |
| Infrequent bowel movements | 1 (0.1%) | - | - | - | - | 2 (0.0%) | 3 (0.0%) |
| Insomnia | 6 (0.4%) | - | 16 (0.4%) | - | 3 (0.6%) | 239 (0.8%) | 264 (0.8%) |
| Intensive care | 1 (0.1%) | - | - | - | - | - | 1 (0.0%) |
| Intentional overdose | 2 (0.1%) | 1 (5.6%) | 4 (0.1%) | 5 (1.3%) | - | 27 (0.1%) | 39 (0.1%) |
| Intentional product misuse | 5 (0.4%) | - | 5 (0.1%) | 4 (1.0%) | 2 (0.4%) | 89 (0.3%) | 105 (0.3%) |
| Intentional product use issue | 3 (0.2%) | - | 3 (0.1%) | 4 (1.0%) | 2 (0.4%) | 26 (0.1%) | 38 (0.1%) |
| Interleukin level increased | 1 (0.1%) | - | - | - | - | - | 1 (0.0%) |
| Intestinal obstruction | 1 (0.1%) | - | - | - | - | 16 (0.1%) | 17 (0.0%) |
| Intracranial aneurysm | 1 (0.1%) | - | - | - | - | 9 (0.0%) | 10 (0.0%) |
| Irritability | 1 (0.1%) | - | 10 (0.3%) | - | - | 37 (0.1%) | 48 (0.1%) |
| Jaundice | 1 (0.1%) | - | 1 (0.0%) | - | - | 7 (0.0%) | 9 (0.0%) |
| Jealous delusion | 1 (0.1%) | - | - | - | - | - | 1 (0.0%) |
| Kidney infection | 1 (0.1%) | - | - | - | - | 6 (0.0%) | 7 (0.0%) |
| Labelled drug-drug interaction medication error | 1 (0.1%) | - | 1 (0.0%) | - | - | 6 (0.0%) | 8 (0.0%) |
| Labile blood pressure | 1 (0.1%) | - | - | - | - | 2 (0.0%) | 3 (0.0%) |
| Lacrimation increased | 1 (0.1%) | - | 1 (0.0%) | - | 1 (0.2%) | 9 (0.0%) | 12 (0.0%) |
| Left atrial enlargement | 1 (0.1%) | - | - | - | - | - | 1 (0.0%) |
| Leg amputation | 1 (0.1%) | - | - | - | 1 (0.2%) | 3 (0.0%) | 5 (0.0%) |
| Leiomyosarcoma | 1 (0.1%) | - | - | - | - | - | 1 (0.0%) |
| Lethargy | 4 (0.3%) | - | 4 (0.1%) | 2 (0.5%) | 2 (0.4%) | 141 (0.5%) | 153 (0.4%) |
| Libido decreased | 1 (0.1%) | - | - | - | 1 (0.2%) | 5 (0.0%) | 7 (0.0%) |
| Lip pain | 1 (0.1%) | - | - | - | - | - | 1 (0.0%) |
| Loss of consciousness | 20 (1.5%) | 1 (5.6%) | 65 (1.7%) | 8 (2.1%) | 3 (0.6%) | 415 (1.4%) | 512 (1.5%) |
| Loss of personal independence in daily activities | 1 (0.1%) | - | 1 (0.0%) | - | - | 35 (0.1%) | 37 (0.1%) |
| Lymphoedema | 1 (0.1%) | - | - | - | - | - | 1 (0.0%) |
| Malaise | 7 (0.5%) | - | 13 (0.3%) | 1 (0.3%) | 2 (0.4%) | 202 (0.7%) | 225 (0.6%) |
| Malignant melanoma | 1 (0.1%) | - | - | - | - | - | 1 (0.0%) |
| Malignant neoplasm of renal pelvis | 1 (0.1%) | - | - | - | - | 1 (0.0%) | 2 (0.0%) |
| Malignant neoplasm of thymus | 1 (0.1%) | - | - | - | - | - | 1 (0.0%) |
| Malignant neoplasm progression | 3 (0.2%) | - | 1 (0.0%) | 1 (0.3%) | - | 69 (0.2%) | 74 (0.2%) |
| Malocclusion | 1 (0.1%) | - | - | - | - | - | 1 (0.0%) |
| Memory impairment | 5 (0.4%) | - | 6 (0.2%) | 1 (0.3%) | 2 (0.4%) | 132 (0.5%) | 146 (0.4%) |
| Mental impairment | 4 (0.3%) | - | 2 (0.1%) | - | 1 (0.2%) | 65 (0.2%) | 72 (0.2%) |
| Mental status changes | 1 (0.1%) | - | 7 (0.2%) | - | 1 (0.2%) | 35 (0.1%) | 44 (0.1%) |
| Metabolic encephalopathy | 1 (0.1%) | - | - | - | - | 6 (0.0%) | 7 (0.0%) |
| Metastases to bone | 1 (0.1%) | - | 1 (0.0%) | 1 (0.3%) | 1 (0.2%) | 3 (0.0%) | 7 (0.0%) |
| Metastases to central nervous system | 2 (0.1%) | - | - | - | - | 3 (0.0%) | 5 (0.0%) |
| Metastases to liver | 1 (0.1%) | - | - | - | - | 4 (0.0%) | 5 (0.0%) |
| Metastases to lung | 1 (0.1%) | - | - | - | 1 (0.2%) | 3 (0.0%) | 5 (0.0%) |
| Metastatic renal cell carcinoma | 1 (0.1%) | - | - | - | - | - | 1 (0.0%) |
| Micturition disorder | 1 (0.1%) | - | - | - | - | 4 (0.0%) | 5 (0.0%) |
| Migraine | 8 (0.6%) | - | - | 1 (0.3%) | - | 84 (0.3%) | 93 (0.3%) |
| Miosis | 7 (0.5%) | - | 14 (0.4%) | 6 (1.5%) | 2 (0.4%) | 160 (0.6%) | 189 (0.5%) |
| Monoplegia | 2 (0.1%) | - | 2 (0.1%) | - | 1 (0.2%) | 8 (0.0%) | 13 (0.0%) |
| Mood swings | 1 (0.1%) | - | 1 (0.0%) | - | - | 18 (0.1%) | 20 (0.1%) |
| Motor dysfunction | 3 (0.2%) | - | 2 (0.1%) | - | - | 5 (0.0%) | 10 (0.0%) |
| Multiple sclerosis | 2 (0.1%) | - | 1 (0.0%) | - | - | 19 (0.1%) | 22 (0.1%) |
| Muscle spasms | 4 (0.3%) | - | 11 (0.3%) | 1 (0.3%) | - | 109 (0.4%) | 125 (0.4%) |
| Muscle strain | 1 (0.1%) | - | - | - | - | 3 (0.0%) | 4 (0.0%) |
| Muscle twitching | 1 (0.1%) | - | 6 (0.2%) | - | 1 (0.2%) | 34 (0.1%) | 42 (0.1%) |
| Muscular weakness | 1 (0.1%) | - | 6 (0.2%) | 1 (0.3%) | - | 46 (0.2%) | 54 (0.2%) |
| Myalgia | 2 (0.1%) | - | 2 (0.1%) | - | - | 24 (0.1%) | 28 (0.1%) |
| Myelopathy | 1 (0.1%) | - | - | - | - | 3 (0.0%) | 4 (0.0%) |
| Myocardial fibrosis | 1 (0.1%) | - | - | - | - | - | 1 (0.0%) |
| Myocardial infarction | 1 (0.1%) | - | - | 1 (0.3%) | 1 (0.2%) | 55 (0.2%) | 58 (0.2%) |
| Myocardial necrosis marker increased | 1 (0.1%) | - | - | - | - | 2 (0.0%) | 3 (0.0%) |
| Myoclonus | 2 (0.1%) | - | 36 (1.0%) | 1 (0.3%) | 2 (0.4%) | 67 (0.2%) | 108 (0.3%) |
| Nausea | 41 (3.0%) | - | 48 (1.3%) | 4 (1.0%) | 10 (2.0%) | 702 (2.4%) | 805 (2.3%) |
| Neck surgery | 1 (0.1%) | - | - | - | - | 1 (0.0%) | 2 (0.0%) |
| Neoplasm malignant | 3 (0.2%) | - | - | - | - | 18 (0.1%) | 21 (0.1%) |
| Neoplasm skin | 1 (0.1%) | - | - | - | - | - | 1 (0.0%) |
| Nervous system disorder | 1 (0.1%) | - | 6 (0.2%) | - | 1 (0.2%) | 14 (0.0%) | 22 (0.1%) |
| Nervousness | 2 (0.1%) | - | 1 (0.0%) | - | - | 57 (0.2%) | 60 (0.2%) |
| Neuroendocrine carcinoma | 1 (0.1%) | - | - | - | - | - | 1 (0.0%) |
| Neurotoxicity | 2 (0.1%) | - | 7 (0.2%) | - | 2 (0.4%) | 16 (0.1%) | 27 (0.1%) |
| Nightmare | 1 (0.1%) | - | 2 (0.1%) | - | - | 24 (0.1%) | 27 (0.1%) |
| Non-small cell lung cancer | 1 (0.1%) | - | - | - | - | 2 (0.0%) | 3 (0.0%) |
| Obstruction | 1 (0.1%) | - | - | - | - | - | 1 (0.0%) |
| Occult blood positive | 1 (0.1%) | - | - | - | - | - | 1 (0.0%) |
| Oedema | 2 (0.1%) | - | - | - | 1 (0.2%) | 14 (0.0%) | 17 (0.0%) |
| Oedema peripheral | 3 (0.2%) | - | 1 (0.0%) | - | 2 (0.4%) | 28 (0.1%) | 34 (0.1%) |
| Oesophageal carcinoma | 2 (0.1%) | - | - | - | 1 (0.2%) | 1 (0.0%) | 4 (0.0%) |
| Oesophageal ulcer | 1 (0.1%) | - | - | - | - | - | 1 (0.0%) |
| Off label use | 18 (1.3%) | 1 (5.6%) | 31 (0.8%) | 4 (1.0%) | 4 (0.8%) | 97 (0.3%) | 155 (0.4%) |
| Oral disorder | 1 (0.1%) | - | - | - | - | - | 1 (0.0%) |
| Oral mucosal blistering | 1 (0.1%) | - | - | - | - | 1 (0.0%) | 2 (0.0%) |
| Oral pain | 1 (0.1%) | - | - | - | - | - | 1 (0.0%) |
| Orthostatic hypotension | 1 (0.1%) | - | 1 (0.0%) | - | - | 14 (0.0%) | 16 (0.0%) |
| Osteoarthritis | 1 (0.1%) | - | - | - | - | 12 (0.0%) | 13 (0.0%) |
| Osteomyelitis | 1 (0.1%) | - | - | - | - | 6 (0.0%) | 7 (0.0%) |
| Osteonecrosis | 1 (0.1%) | - | - | - | - | 5 (0.0%) | 6 (0.0%) |
| Osteosarcoma metastatic | 1 (0.1%) | - | - | - | - | - | 1 (0.0%) |
| Ovarian disorder | 1 (0.1%) | - | - | - | - | - | 1 (0.0%) |
| Overdose | 9 (0.7%) | - | 26 (0.7%) | 13 (3.3%) | 15 (2.9%) | 452 (1.6%) | 515 (1.5%) |
| Oxygen saturation | 1 (0.1%) | - | - | - | - | - | 1 (0.0%) |
| Oxygen saturation decreased | 3 (0.2%) | - | 28 (0.7%) | 2 (0.5%) | 1 (0.2%) | 40 (0.1%) | 74 (0.2%) |
| Pachymeningitis | 1 (0.1%) | - | - | - | - | - | 1 (0.0%) |
| Pain | 24 (1.8%) | - | 21 (0.6%) | 2 (0.5%) | 5 (1.0%) | 420 (1.5%) | 472 (1.3%) |
| Pain in extremity | 2 (0.1%) | - | 1 (0.0%) | 1 (0.3%) | 1 (0.2%) | 71 (0.2%) | 76 (0.2%) |
| Pallor | 2 (0.1%) | - | 5 (0.1%) | - | - | 19 (0.1%) | 26 (0.1%) |
| Palpitations | 3 (0.2%) | - | 2 (0.1%) | 1 (0.3%) | - | 63 (0.2%) | 69 (0.2%) |
| Pancreatic carcinoma | 1 (0.1%) | - | - | - | - | 6 (0.0%) | 7 (0.0%) |
| Pancreatitis | 1 (0.1%) | - | - | - | - | 6 (0.0%) | 7 (0.0%) |
| Paradoxical pain | 1 (0.1%) | - | - | - | 2 (0.4%) | - | 3 (0.0%) |
| Paraesthesia | 6 (0.4%) | - | 7 (0.2%) | 1 (0.3%) | - | 102 (0.4%) | 116 (0.3%) |
| Paralysis | 3 (0.2%) | - | 5 (0.1%) | 1 (0.3%) | - | 17 (0.1%) | 26 (0.1%) |
| Paraplegia | 1 (0.1%) | - | 4 (0.1%) | - | - | 3 (0.0%) | 8 (0.0%) |
| Pelvic deformity | 1 (0.1%) | - | - | - | - | - | 1 (0.0%) |
| Pelvic pain | 1 (0.1%) | - | 1 (0.0%) | - | - | 3 (0.0%) | 5 (0.0%) |
| Peptic ulcer | 1 (0.1%) | - | - | - | - | 2 (0.0%) | 3 (0.0%) |
| Peripheral sensory neuropathy | 1 (0.1%) | - | - | - | - | 2 (0.0%) | 3 (0.0%) |
| Phantom limb syndrome | 1 (0.1%) | - | 1 (0.0%) | - | - | 4 (0.0%) | 6 (0.0%) |
| Pharyngitis | 1 (0.1%) | - | - | - | - | 1 (0.0%) | 2 (0.0%) |
| Photophobia | 1 (0.1%) | - | 1 (0.0%) | - | - | 8 (0.0%) | 10 (0.0%) |
| Piloerection | 1 (0.1%) | - | - | - | - | 3 (0.0%) | 4 (0.0%) |
| Platelet count decreased | 1 (0.1%) | - | 2 (0.1%) | - | - | 11 (0.0%) | 14 (0.0%) |
| Pneumonia | 3 (0.2%) | - | 4 (0.1%) | 1 (0.3%) | 3 (0.6%) | 124 (0.4%) | 135 (0.4%) |
| Pneumonia aspiration | 4 (0.3%) | - | 4 (0.1%) | 2 (0.5%) | - | 39 (0.1%) | 49 (0.1%) |
| Polyneuropathy | 1 (0.1%) | - | - | - | - | - | 1 (0.0%) |
| Polyneuropathy in malignant disease | 1 (0.1%) | - | - | - | - | - | 1 (0.0%) |
| Poor dental condition | 1 (0.1%) | - | - | - | - | - | 1 (0.0%) |
| Poor quality product administered | 1 (0.1%) | - | - | 1 (0.3%) | - | 10 (0.0%) | 12 (0.0%) |
| Premature labour | 1 (0.1%) | - | - | - | - | - | 1 (0.0%) |
| Presyncope | 3 (0.2%) | - | 9 (0.2%) | - | - | 22 (0.1%) | 34 (0.1%) |
| Proctalgia | 1 (0.1%) | - | - | - | - | 1 (0.0%) | 2 (0.0%) |
| Product administration error | 3 (0.2%) | - | 3 (0.1%) | 1 (0.3%) | - | 98 (0.3%) | 105 (0.3%) |
| Product availability issue | 1 (0.1%) | - | - | - | - | 6 (0.0%) | 7 (0.0%) |
| Product dispensing error | 1 (0.1%) | - | 1 (0.0%) | 1 (0.3%) | - | 15 (0.1%) | 18 (0.1%) |
| Product dose omission issue | 2 (0.1%) | - | - | - | - | 109 (0.4%) | 111 (0.3%) |
| Product physical issue | 1 (0.1%) | - | - | - | - | 12 (0.0%) | 13 (0.0%) |
| Product prescribing error | 25 (1.9%) | - | - | 1 (0.3%) | - | 48 (0.2%) | 74 (0.2%) |
| Product quality issue | 1 (0.1%) | - | - | 2 (0.5%) | - | 48 (0.2%) | 51 (0.1%) |
| Product solubility abnormal | 1 (0.1%) | - | - | - | - | - | 1 (0.0%) |
| Product substitution issue | 2 (0.1%) | - | - | - | - | 21 (0.1%) | 23 (0.1%) |
| Product supply issue | 1 (0.1%) | - | - | - | - | - | 1 (0.0%) |
| Product taste abnormal | 3 (0.2%) | - | - | - | - | - | 3 (0.0%) |
| Product use complaint | 1 (0.1%) | - | - | - | - | 1 (0.0%) | 2 (0.0%) |
| Product use in unapproved indication | 6 (0.4%) | 1 (5.6%) | 7 (0.2%) | 2 (0.5%) | 3 (0.6%) | 35 (0.1%) | 54 (0.2%) |
| Product use issue | 1 (0.1%) | - | 7 (0.2%) | 2 (0.5%) | - | 12 (0.0%) | 22 (0.1%) |
| Prostate cancer | 1 (0.1%) | - | - | - | - | 10 (0.0%) | 11 (0.0%) |
| Pruritus | 9 (0.7%) | - | 9 (0.2%) | - | - | 89 (0.3%) | 107 (0.3%) |
| Psychomotor hyperactivity | 1 (0.1%) | - | 6 (0.2%) | - | - | 42 (0.1%) | 49 (0.1%) |
| Psychomotor skills impaired | 2 (0.1%) | - | 1 (0.0%) | - | - | 10 (0.0%) | 13 (0.0%) |
| Psychopathic personality | 1 (0.1%) | - | - | - | - | - | 1 (0.0%) |
| Pustule | 1 (0.1%) | - | - | - | - | 1 (0.0%) | 2 (0.0%) |
| Pyrexia | 6 (0.4%) | - | 48 (1.3%) | 2 (0.5%) | 6 (1.2%) | 136 (0.5%) | 198 (0.6%) |
| Quadriparesis | 1 (0.1%) | - | 1 (0.0%) | - | - | 2 (0.0%) | 4 (0.0%) |
| Quality of life decreased | 1 (0.1%) | - | - | - | - | 4 (0.0%) | 5 (0.0%) |
| Rash | 1 (0.1%) | - | 7 (0.2%) | - | - | 62 (0.2%) | 70 (0.2%) |
| Rectal haemorrhage | 1 (0.1%) | - | 7 (0.2%) | - | - | 6 (0.0%) | 14 (0.0%) |
| Renal cancer | 1 (0.1%) | - | - | - | 1 (0.2%) | 1 (0.0%) | 3 (0.0%) |
| Renal failure | 3 (0.2%) | - | 3 (0.1%) | - | 2 (0.4%) | 48 (0.2%) | 56 (0.2%) |
| Renal impairment | 1 (0.1%) | - | 6 (0.2%) | - | - | 16 (0.1%) | 23 (0.1%) |
| Respiratory arrest | 2 (0.1%) | - | 28 (0.7%) | 2 (0.5%) | 1 (0.2%) | 67 (0.2%) | 100 (0.3%) |
| Respiratory depression | 12 (0.9%) | - | 52 (1.4%) | 7 (1.8%) | 12 (2.3%) | 225 (0.8%) | 308 (0.9%) |
| Respiratory distress | 4 (0.3%) | - | 4 (0.1%) | 2 (0.5%) | 3 (0.6%) | 39 (0.1%) | 52 (0.1%) |
| Respiratory failure | 2 (0.1%) | - | 11 (0.3%) | 3 (0.8%) | 1 (0.2%) | 76 (0.3%) | 93 (0.3%) |
| Respiratory rate decreased | 1 (0.1%) | - | 5 (0.1%) | 1 (0.3%) | 3 (0.6%) | 41 (0.1%) | 51 (0.1%) |
| Respiratory rate increased | 1 (0.1%) | - | 2 (0.1%) | - | - | 2 (0.0%) | 5 (0.0%) |
| Restless legs syndrome | 1 (0.1%) | - | - | - | 1 (0.2%) | 38 (0.1%) | 40 (0.1%) |
| Restlessness | 3 (0.2%) | - | 12 (0.3%) | 2 (0.5%) | 4 (0.8%) | 105 (0.4%) | 126 (0.4%) |
| Retching | 2 (0.1%) | - | 2 (0.1%) | - | - | 15 (0.1%) | 19 (0.1%) |
| Rhabdomyolysis | 1 (0.1%) | - | 8 (0.2%) | - | - | 22 (0.1%) | 31 (0.1%) |
| Rhinorrhoea | 2 (0.1%) | - | 1 (0.0%) | - | - | 16 (0.1%) | 19 (0.1%) |
| Rib fracture | 1 (0.1%) | - | 1 (0.0%) | - | - | 15 (0.1%) | 17 (0.0%) |
| Road traffic accident | 2 (0.1%) | - | 2 (0.1%) | - | 1 (0.2%) | 46 (0.2%) | 51 (0.1%) |
| Sciatica | 1 (0.1%) | - | - | - | - | 17 (0.1%) | 18 (0.1%) |
| Sedation | 2 (0.1%) | - | 19 (0.5%) | 2 (0.5%) | 6 (1.2%) | 142 (0.5%) | 171 (0.5%) |
| Seizure | 18 (1.3%) | 1 (5.6%) | 64 (1.7%) | 3 (0.8%) | 8 (1.6%) | 304 (1.1%) | 398 (1.1%) |
| Sepsis | 2 (0.1%) | - | 5 (0.1%) | - | 2 (0.4%) | 29 (0.1%) | 38 (0.1%) |
| Serotonin syndrome | 4 (0.3%) | - | 89 (2.4%) | 1 (0.3%) | 1 (0.2%) | 66 (0.2%) | 161 (0.5%) |
| Sinusitis | 1 (0.1%) | - | - | - | - | 14 (0.0%) | 15 (0.0%) |
| Skin lesion | 1 (0.1%) | - | - | - | - | 5 (0.0%) | 6 (0.0%) |
| Sleep apnoea syndrome | 1 (0.1%) | - | - | - | 1 (0.2%) | 20 (0.1%) | 22 (0.1%) |
| Sleep disorder | 1 (0.1%) | - | 1 (0.0%) | - | 1 (0.2%) | 22 (0.1%) | 25 (0.1%) |
| Small cell lung cancer | 1 (0.1%) | - | - | - | - | 1 (0.0%) | 2 (0.0%) |
| Social avoidant behaviour | 2 (0.1%) | - | 2 (0.1%) | - | - | 3 (0.0%) | 7 (0.0%) |
| Soliloquy | 2 (0.1%) | - | - | - | 1 (0.2%) | - | 3 (0.0%) |
| Somnambulism | 1 (0.1%) | - | - | 1 (0.3%) | - | 5 (0.0%) | 7 (0.0%) |
| Somnolence | 86 (6.4%) | 1 (5.6%) | 67 (1.8%) | 14 (3.6%) | 40 (7.8%) | 1163 (4.0%) | 1371 (3.9%) |
| Speech disorder | 2 (0.1%) | - | 2 (0.1%) | - | 2 (0.4%) | 76 (0.3%) | 82 (0.2%) |
| Spinal column injury | 1 (0.1%) | - | - | - | - | - | 1 (0.0%) |
| Spinal cord injury | 1 (0.1%) | - | - | - | - | 3 (0.0%) | 4 (0.0%) |
| Spinal fracture | 1 (0.1%) | - | - | - | - | 6 (0.0%) | 7 (0.0%) |
| Staphylococcal infection | 1 (0.1%) | - | - | - | 1 (0.2%) | 20 (0.1%) | 22 (0.1%) |
| Stoma prolapse | 1 (0.1%) | - | - | - | - | - | 1 (0.0%) |
| Stomatitis | 1 (0.1%) | - | - | - | - | 11 (0.0%) | 12 (0.0%) |
| Streptococcus test positive | 1 (0.1%) | - | - | - | - | - | 1 (0.0%) |
| Stress | 2 (0.1%) | - | - | - | - | 16 (0.1%) | 18 (0.1%) |
| Stupor | 1 (0.1%) | - | 7 (0.2%) | - | - | 42 (0.1%) | 50 (0.1%) |
| Substance abuse | 1 (0.1%) | - | 3 (0.1%) | 3 (0.8%) | - | 6 (0.0%) | 13 (0.0%) |
| Suffocation feeling | 1 (0.1%) | - | 1 (0.0%) | - | - | 1 (0.0%) | 3 (0.0%) |
| Suicidal ideation | 2 (0.1%) | - | 2 (0.1%) | 1 (0.3%) | 1 (0.2%) | 66 (0.2%) | 72 (0.2%) |
| Suicide attempt | 5 (0.4%) | - | 3 (0.1%) | 3 (0.8%) | 3 (0.6%) | 39 (0.1%) | 53 (0.2%) |
| Swelling | 1 (0.1%) | - | 2 (0.1%) | - | - | 15 (0.1%) | 18 (0.1%) |
| Swelling face | 2 (0.1%) | - | 2 (0.1%) | 1 (0.3%) | - | 9 (0.0%) | 14 (0.0%) |
| Syncope | 2 (0.1%) | - | 6 (0.2%) | 1 (0.3%) | 1 (0.2%) | 117 (0.4%) | 127 (0.4%) |
| Tachycardia | 5 (0.4%) | - | 40 (1.1%) | 2 (0.5%) | 1 (0.2%) | 63 (0.2%) | 111 (0.3%) |
| Taste disorder | 3 (0.2%) | - | - | 1 (0.3%) | 1 (0.2%) | 23 (0.1%) | 28 (0.1%) |
| Teeth brittle | 1 (0.1%) | - | - | - | - | - | 1 (0.0%) |
| Temporomandibular joint syndrome | 1 (0.1%) | - | - | - | - | 1 (0.0%) | 2 (0.0%) |
| Therapeutic product effect decreased | 1 (0.1%) | - | - | - | - | 78 (0.3%) | 79 (0.2%) |
| Therapeutic product effect increased | 1 (0.1%) | - | 1 (0.0%) | - | 1 (0.2%) | 50 (0.2%) | 53 (0.2%) |
| Therapeutic response unexpected | 1 (0.1%) | - | - | - | - | 10 (0.0%) | 11 (0.0%) |
| Thermal burn | 1 (0.1%) | - | - | - | - | 1 (0.0%) | 2 (0.0%) |
| Thinking abnormal | 4 (0.3%) | - | - | - | - | 21 (0.1%) | 25 (0.1%) |
| Thrombosis | 3 (0.2%) | - | - | - | - | 14 (0.0%) | 17 (0.0%) |
| Tic | 1 (0.1%) | - | - | - | - | 2 (0.0%) | 3 (0.0%) |
| Toe amputation | 1 (0.1%) | - | - | - | - | 1 (0.0%) | 2 (0.0%) |
| Tooth abscess | 1 (0.1%) | - | - | - | 1 (0.2%) | 3 (0.0%) | 5 (0.0%) |
| Tooth disorder | 1 (0.1%) | - | - | - | - | 1 (0.0%) | 2 (0.0%) |
| Tooth extraction | 1 (0.1%) | - | - | - | - | 1 (0.0%) | 2 (0.0%) |
| Tooth fracture | 2 (0.1%) | - | - | - | - | 4 (0.0%) | 6 (0.0%) |
| Tooth infection | 1 (0.1%) | - | - | - | - | - | 1 (0.0%) |
| Tooth loss | 7 (0.5%) | - | - | - | 1 (0.2%) | 6 (0.0%) | 14 (0.0%) |
| Toothache | 1 (0.1%) | - | - | - | - | 1 (0.0%) | 2 (0.0%) |
| Toxicity to various agents | 10 (0.7%) | - | 11 (0.3%) | 6 (1.5%) | 2 (0.4%) | 195 (0.7%) | 224 (0.6%) |
| Transient ischaemic attack | 1 (0.1%) | - | 1 (0.0%) | 1 (0.3%) | - | 24 (0.1%) | 27 (0.1%) |
| Treatment failure | 1 (0.1%) | - | - | - | - | 1 (0.0%) | 2 (0.0%) |
| Tremor | 15 (1.1%) | 1 (5.6%) | 40 (1.1%) | 2 (0.5%) | 3 (0.6%) | 363 (1.3%) | 424 (1.2%) |
| Ulcer | 1 (0.1%) | - | - | - | - | 3 (0.0%) | 4 (0.0%) |
| Unevaluable event | 4 (0.3%) | - | - | 2 (0.5%) | - | 31 (0.1%) | 37 (0.1%) |
| Unresponsive to stimuli | 6 (0.4%) | - | 59 (1.6%) | 10 (2.6%) | 3 (0.6%) | 146 (0.5%) | 224 (0.6%) |
| Urethral disorder | 1 (0.1%) | - | - | - | - | 1 (0.0%) | 2 (0.0%) |
| Urethral ulcer | 1 (0.1%) | - | - | - | - | - | 1 (0.0%) |
| Urinary retention | 4 (0.3%) | - | 2 (0.1%) | - | - | 49 (0.2%) | 55 (0.2%) |
| Uterine disorder | 1 (0.1%) | - | - | - | - | 1 (0.0%) | 2 (0.0%) |
| Vasodilatation | 1 (0.1%) | - | 1 (0.0%) | - | - | - | 2 (0.0%) |
| Ventricular tachycardia | 1 (0.1%) | - | 7 (0.2%) | - | - | 3 (0.0%) | 11 (0.0%) |
| Vertigo | 2 (0.1%) | - | 2 (0.1%) | 4 (1.0%) | - | 54 (0.2%) | 62 (0.2%) |
| Viral infection | 1 (0.1%) | - | - | - | - | 6 (0.0%) | 7 (0.0%) |
| Vision blurred | 1 (0.1%) | - | 4 (0.1%) | - | 1 (0.2%) | 62 (0.2%) | 68 (0.2%) |
| Vomiting | 30 (2.2%) | - | 44 (1.2%) | 7 (1.8%) | 7 (1.4%) | 517 (1.8%) | 605 (1.7%) |
| Vulval ulceration | 1 (0.1%) | - | - | - | - | - | 1 (0.0%) |
| Vulvar erosion | 1 (0.1%) | - | - | - | - | - | 1 (0.0%) |
| Weight bearing difficulty | 1 (0.1%) | - | - | - | - | - | 1 (0.0%) |
| Weight decreased | 7 (0.5%) | - | 8 (0.2%) | 1 (0.3%) | - | 164 (0.6%) | 180 (0.5%) |
| Weight increased | 3 (0.2%) | - | - | 1 (0.3%) | 1 (0.2%) | 40 (0.1%) | 45 (0.1%) |
| White blood cell count abnormal | 1 (0.1%) | - | - | - | - | - | 1 (0.0%) |
| White blood cell count decreased | 1 (0.1%) | - | 1 (0.0%) | - | - | 5 (0.0%) | 7 (0.0%) |
| Withdrawal syndrome | 21 (1.6%) | - | 16 (0.4%) | 2 (0.5%) | 2 (0.4%) | 402 (1.4%) | 443 (1.3%) |
| Wound | 1 (0.1%) | - | - | - | - | 5 (0.0%) | 6 (0.0%) |
| Wrong technique in product usage process | 2 (0.1%) | - | 2 (0.1%) | 1 (0.3%) | 2 (0.4%) | 344 (1.2%) | 351 (1.0%) |
| Yawning | 1 (0.1%) | - | - | - | - | 13 (0.0%) | 14 (0.0%) |
| Cardiac arrest | - | 1 (5.6%) | 24 (0.6%) | 3 (0.8%) | - | 31 (0.1%) | 59 (0.2%) |
| Abdominal compartment syndrome | - | - | 1 (0.0%) | - | - | - | 1 (0.0%) |
| Abdominal mass | - | - | 1 (0.0%) | - | - | - | 1 (0.0%) |
| Abdominal pain lower | - | - | 4 (0.1%) | - | - | 6 (0.0%) | 10 (0.0%) |
| Abdominal rigidity | - | - | 1 (0.0%) | - | - | 1 (0.0%) | 2 (0.0%) |
| Abdominal wall haematoma | - | - | 1 (0.0%) | - | - | - | 1 (0.0%) |
| Abortion induced | - | - | 1 (0.0%) | - | - | - | 1 (0.0%) |
| Acidosis | - | - | 2 (0.1%) | - | - | 5 (0.0%) | 7 (0.0%) |
| Acute lung injury | - | - | 1 (0.0%) | - | - | - | 1 (0.0%) |
| Acute myocardial infarction | - | - | 3 (0.1%) | - | - | 2 (0.0%) | 5 (0.0%) |
| Acute pulmonary oedema | - | - | 1 (0.0%) | - | 1 (0.2%) | 4 (0.0%) | 6 (0.0%) |
| Acute respiratory distress syndrome | - | - | 2 (0.1%) | 2 (0.5%) | - | 4 (0.0%) | 8 (0.0%) |
| Acute sinusitis | - | - | 1 (0.0%) | - | - | 1 (0.0%) | 2 (0.0%) |
| Adverse drug reaction | - | - | 1 (0.0%) | - | - | 26 (0.1%) | 27 (0.1%) |
| Agitation neonatal | - | - | 1 (0.0%) | - | - | - | 1 (0.0%) |
| Airway complication of anaesthesia | - | - | 1 (0.0%) | - | - | - | 1 (0.0%) |
| Akinesia | - | - | 3 (0.1%) | - | - | - | 3 (0.0%) |
| Alanine aminotransferase decreased | - | - | 1 (0.0%) | - | - | - | 1 (0.0%) |
| Alanine aminotransferase increased | - | - | 3 (0.1%) | - | 1 (0.2%) | 11 (0.0%) | 15 (0.0%) |
| Alcohol abuse | - | - | 2 (0.1%) | - | - | - | 2 (0.0%) |
| Amimia | - | - | 1 (0.0%) | - | - | 1 (0.0%) | 2 (0.0%) |
| Ammonia increased | - | - | 1 (0.0%) | - | - | 1 (0.0%) | 2 (0.0%) |
| Amnestic disorder | - | - | 1 (0.0%) | 1 (0.3%) | - | 3 (0.0%) | 5 (0.0%) |
| Amylase increased | - | - | 2 (0.1%) | - | - | 1 (0.0%) | 3 (0.0%) |
| Anaesthetic complication | - | - | 4 (0.1%) | - | - | - | 4 (0.0%) |
| Anaesthetic complication cardiac | - | - | 1 (0.0%) | - | - | - | 1 (0.0%) |
| Anaesthetic complication pulmonary | - | - | 1 (0.0%) | - | - | - | 1 (0.0%) |
| Anaphylactic reaction | - | - | 2 (0.1%) | - | - | 1 (0.0%) | 3 (0.0%) |
| Anaphylactic shock | - | - | 6 (0.2%) | - | - | 1 (0.0%) | 7 (0.0%) |
| Anger | - | - | 1 (0.0%) | - | - | 13 (0.0%) | 14 (0.0%) |
| Angioedema | - | - | 3 (0.1%) | - | - | 2 (0.0%) | 5 (0.0%) |
| Angle closure glaucoma | - | - | 1 (0.0%) | - | - | - | 1 (0.0%) |
| Anhidrosis | - | - | 1 (0.0%) | - | - | - | 1 (0.0%) |
| Anisocoria | - | - | 2 (0.1%) | - | - | 2 (0.0%) | 4 (0.0%) |
| Anosmia | - | - | 4 (0.1%) | 1 (0.3%) | - | 1 (0.0%) | 6 (0.0%) |
| Anterograde amnesia | - | - | 4 (0.1%) | - | - | - | 4 (0.0%) |
| Anticholinergic syndrome | - | - | 5 (0.1%) | - | - | - | 5 (0.0%) |
| Anticonvulsant drug level increased | - | - | 2 (0.1%) | - | - | 1 (0.0%) | 3 (0.0%) |
| Antidepressant drug level increased | - | - | 1 (0.0%) | - | - | 1 (0.0%) | 2 (0.0%) |
| Antiphospholipid antibodies positive | - | - | 1 (0.0%) | - | - | - | 1 (0.0%) |
| Anuria | - | - | 4 (0.1%) | - | - | 5 (0.0%) | 9 (0.0%) |
| Anxiety disorder | - | - | 1 (0.0%) | - | - | 3 (0.0%) | 4 (0.0%) |
| Apallic syndrome | - | - | 2 (0.1%) | - | - | 1 (0.0%) | 3 (0.0%) |
| Apathy | - | - | 1 (0.0%) | - | 1 (0.2%) | 12 (0.0%) | 14 (0.0%) |
| Aphasia | - | - | 6 (0.2%) | - | - | 43 (0.1%) | 49 (0.1%) |
| Appendicitis | - | - | 1 (0.0%) | - | - | - | 1 (0.0%) |
| Appendicitis perforated | - | - | 1 (0.0%) | - | - | - | 1 (0.0%) |
| Arachnoiditis | - | - | 1 (0.0%) | - | - | 6 (0.0%) | 7 (0.0%) |
| Areflexia | - | - | 8 (0.2%) | - | - | 3 (0.0%) | 11 (0.0%) |
| Arrhythmia | - | - | 3 (0.1%) | - | - | 13 (0.0%) | 16 (0.0%) |
| Arthritis | - | - | 1 (0.0%) | - | - | 21 (0.1%) | 22 (0.1%) |
| Arthropathy | - | - | 1 (0.0%) | - | - | 14 (0.0%) | 15 (0.0%) |
| Ascites | - | - | 1 (0.0%) | - | 1 (0.2%) | 5 (0.0%) | 7 (0.0%) |
| Aspartate aminotransferase increased | - | - | 2 (0.1%) | - | 1 (0.2%) | 15 (0.1%) | 18 (0.1%) |
| Asphyxia | - | - | 2 (0.1%) | 1 (0.3%) | 1 (0.2%) | 7 (0.0%) | 11 (0.0%) |
| Asterixis | - | - | 1 (0.0%) | - | - | 3 (0.0%) | 4 (0.0%) |
| Asthma | - | - | 3 (0.1%) | - | - | 9 (0.0%) | 12 (0.0%) |
| Atelectasis | - | - | 3 (0.1%) | - | - | 11 (0.0%) | 14 (0.0%) |
| Atrial fibrillation | - | - | 1 (0.0%) | - | 1 (0.2%) | 22 (0.1%) | 24 (0.1%) |
| Atrial septal defect | - | - | 1 (0.0%) | - | - | - | 1 (0.0%) |
| Atrioventricular block | - | - | 1 (0.0%) | - | - | 2 (0.0%) | 3 (0.0%) |
| Atrioventricular block complete | - | - | 1 (0.0%) | - | - | 2 (0.0%) | 3 (0.0%) |
| Atrioventricular block second degree | - | - | 2 (0.1%) | - | - | - | 2 (0.0%) |
| Atypical pneumonia | - | - | 1 (0.0%) | - | - | - | 1 (0.0%) |
| Autonomic dysreflexia | - | - | 4 (0.1%) | - | - | - | 4 (0.0%) |
| Autonomic nervous system imbalance | - | - | 4 (0.1%) | - | - | 3 (0.0%) | 7 (0.0%) |
| Back disorder | - | - | 1 (0.0%) | - | - | 16 (0.1%) | 17 (0.0%) |
| Bacteraemia | - | - | 2 (0.1%) | - | 1 (0.2%) | 4 (0.0%) | 7 (0.0%) |
| Basal ganglion degeneration | - | - | 1 (0.0%) | - | - | - | 1 (0.0%) |
| Behaviour disorder | - | - | 1 (0.0%) | - | 1 (0.2%) | 7 (0.0%) | 9 (0.0%) |
| Binocular eye movement disorder | - | - | 2 (0.1%) | - | - | - | 2 (0.0%) |
| Bladder disorder | - | - | 1 (0.0%) | - | - | 2 (0.0%) | 3 (0.0%) |
| Bladder pain | - | - | 1 (0.0%) | - | - | 4 (0.0%) | 5 (0.0%) |
| Bladder perforation | - | - | 1 (0.0%) | - | - | - | 1 (0.0%) |
| Bladder spasm | - | - | 1 (0.0%) | - | - | - | 1 (0.0%) |
| Blindness | - | - | 2 (0.1%) | - | - | 12 (0.0%) | 14 (0.0%) |
| Blindness cortical | - | - | 1 (0.0%) | - | - | 1 (0.0%) | 2 (0.0%) |
| Blindness transient | - | - | 1 (0.0%) | - | - | 3 (0.0%) | 4 (0.0%) |
| Blood bilirubin increased | - | - | 1 (0.0%) | - | 1 (0.2%) | 1 (0.0%) | 3 (0.0%) |
| Blood carbon monoxide increased | - | - | 1 (0.0%) | - | - | - | 1 (0.0%) |
| Blood creatine phosphokinase increased | - | - | 14 (0.4%) | - | - | 8 (0.0%) | 22 (0.1%) |
| Blood creatinine increased | - | - | 2 (0.1%) | - | - | 8 (0.0%) | 10 (0.0%) |
| Blood fibrinogen decreased | - | - | 1 (0.0%) | - | - | - | 1 (0.0%) |
| Blood lactate dehydrogenase increased | - | - | 2 (0.1%) | - | - | 2 (0.0%) | 4 (0.0%) |
| Blood pH decreased | - | - | 1 (0.0%) | - | - | 1 (0.0%) | 2 (0.0%) |
| Blood potassium increased | - | - | 3 (0.1%) | - | - | 2 (0.0%) | 5 (0.0%) |
| Blood pressure abnormal | - | - | 1 (0.0%) | - | - | 7 (0.0%) | 8 (0.0%) |
| Blood pressure fluctuation | - | - | 1 (0.0%) | - | - | 9 (0.0%) | 10 (0.0%) |
| Blood pressure immeasurable | - | - | 1 (0.0%) | - | 1 (0.2%) | - | 2 (0.0%) |
| Blood pressure increased | - | - | 20 (0.5%) | - | 1 (0.2%) | 71 (0.2%) | 92 (0.3%) |
| Blood pressure systolic decreased | - | - | 1 (0.0%) | - | - | - | 1 (0.0%) |
| Blood pressure systolic increased | - | - | 3 (0.1%) | - | - | 1 (0.0%) | 4 (0.0%) |
| Blood urea increased | - | - | 1 (0.0%) | - | - | 16 (0.1%) | 17 (0.0%) |
| Blood urine present | - | - | 1 (0.0%) | - | - | 3 (0.0%) | 4 (0.0%) |
| Body temperature increased | - | - | 6 (0.2%) | - | - | 25 (0.1%) | 31 (0.1%) |
| Brain death | - | - | 2 (0.1%) | - | - | 1 (0.0%) | 3 (0.0%) |
| Brain herniation | - | - | 1 (0.0%) | - | - | 1 (0.0%) | 2 (0.0%) |
| Brain hypoxia | - | - | 2 (0.1%) | - | - | 4 (0.0%) | 6 (0.0%) |
| Brain natriuretic peptide increased | - | - | 1 (0.0%) | - | - | - | 1 (0.0%) |
| Bronchitis | - | - | 1 (0.0%) | - | - | 16 (0.1%) | 17 (0.0%) |
| Bronchopulmonary dysplasia | - | - | 1 (0.0%) | - | - | - | 1 (0.0%) |
| Bronchospasm | - | - | 2 (0.1%) | - | - | 3 (0.0%) | 5 (0.0%) |
| Bundle branch block left | - | - | 1 (0.0%) | - | - | 1 (0.0%) | 2 (0.0%) |
| Caesarean section | - | - | 1 (0.0%) | - | - | 1 (0.0%) | 2 (0.0%) |
| Cancer pain | - | - | 1 (0.0%) | - | - | 6 (0.0%) | 7 (0.0%) |
| Candida sepsis | - | - | 1 (0.0%) | - | - | - | 1 (0.0%) |
| Capillary permeability increased | - | - | 1 (0.0%) | - | - | - | 1 (0.0%) |
| Carcinoid syndrome | - | - | 1 (0.0%) | - | - | - | 1 (0.0%) |
| Cardiac failure | - | - | 4 (0.1%) | - | 1 (0.2%) | 17 (0.1%) | 22 (0.1%) |
| Cardiac failure acute | - | - | 1 (0.0%) | - | - | 1 (0.0%) | 2 (0.0%) |
| Cardiac failure congestive | - | - | 2 (0.1%) | - | - | 24 (0.1%) | 26 (0.1%) |
| Cardiac output decreased | - | - | 1 (0.0%) | - | - | - | 1 (0.0%) |
| Cardiac valve vegetation | - | - | 1 (0.0%) | - | - | - | 1 (0.0%) |
| Cardio-respiratory distress | - | - | 1 (0.0%) | - | - | - | 1 (0.0%) |
| Cardiogenic shock | - | - | 3 (0.1%) | - | - | 1 (0.0%) | 4 (0.0%) |
| Cardiomegaly | - | - | 2 (0.1%) | 1 (0.3%) | - | 4 (0.0%) | 7 (0.0%) |
| Cardiomyopathy | - | - | 1 (0.0%) | - | - | 4 (0.0%) | 5 (0.0%) |
| Cardiovascular insufficiency | - | - | 1 (0.0%) | - | - | 1 (0.0%) | 2 (0.0%) |
| Cataract | - | - | 1 (0.0%) | - | - | 9 (0.0%) | 10 (0.0%) |
| Catheter placement | - | - | 2 (0.1%) | - | - | - | 2 (0.0%) |
| Catheter site infection | - | - | 1 (0.0%) | - | - | - | 1 (0.0%) |
| Cerebellar haemorrhage | - | - | 2 (0.1%) | - | - | - | 2 (0.0%) |
| Cerebellar infarction | - | - | 1 (0.0%) | - | - | 1 (0.0%) | 2 (0.0%) |
| Cerebral haematoma | - | - | 1 (0.0%) | - | - | - | 1 (0.0%) |
| Cerebral haemorrhage | - | - | 3 (0.1%) | 1 (0.3%) | - | 18 (0.1%) | 22 (0.1%) |
| Cerebral infarction | - | - | 7 (0.2%) | - | - | 8 (0.0%) | 15 (0.0%) |
| Cerebrospinal fluid leakage | - | - | 2 (0.1%) | - | - | 4 (0.0%) | 6 (0.0%) |
| Cerebrovascular arteriovenous malformation | - | - | 1 (0.0%) | - | - | - | 1 (0.0%) |
| Cerebrovascular disorder | - | - | 1 (0.0%) | - | - | 3 (0.0%) | 4 (0.0%) |
| Chest injury | - | - | 1 (0.0%) | - | - | 3 (0.0%) | 4 (0.0%) |
| Chest pain | - | - | 5 (0.1%) | - | 2 (0.4%) | 85 (0.3%) | 92 (0.3%) |
| Cholelithiasis | - | - | 1 (0.0%) | - | 1 (0.2%) | 8 (0.0%) | 10 (0.0%) |
| Chorea | - | - | 1 (0.0%) | - | - | 4 (0.0%) | 5 (0.0%) |
| Choreoathetosis | - | - | 4 (0.1%) | - | 1 (0.2%) | 4 (0.0%) | 9 (0.0%) |
| Chromaturia | - | - | 2 (0.1%) | - | 1 (0.2%) | - | 3 (0.0%) |
| Chronic sinusitis | - | - | 7 (0.2%) | - | - | - | 7 (0.0%) |
| Circadian rhythm sleep disorder | - | - | 1 (0.0%) | - | - | 2 (0.0%) | 3 (0.0%) |
| Circulatory collapse | - | - | 6 (0.2%) | - | - | 14 (0.0%) | 20 (0.1%) |
| Clonic convulsion | - | - | 3 (0.1%) | - | - | 3 (0.0%) | 6 (0.0%) |
| Clonus | - | - | 15 (0.4%) | - | - | 7 (0.0%) | 22 (0.1%) |
| Coagulopathy | - | - | 6 (0.2%) | - | 1 (0.2%) | 3 (0.0%) | 10 (0.0%) |
| Cognitive disorder | - | - | 6 (0.2%) | - | 3 (0.6%) | 63 (0.2%) | 72 (0.2%) |
| Cogwheel rigidity | - | - | 1 (0.0%) | - | - | 1 (0.0%) | 2 (0.0%) |
| Colitis | - | - | 6 (0.2%) | - | - | 3 (0.0%) | 9 (0.0%) |
| Colitis ischaemic | - | - | 1 (0.0%) | - | - | 1 (0.0%) | 2 (0.0%) |
| Colitis ulcerative | - | - | 7 (0.2%) | - | - | - | 7 (0.0%) |
| Colour blindness | - | - | 1 (0.0%) | - | - | - | 1 (0.0%) |
| Coma scale abnormal | - | - | 6 (0.2%) | - | - | 7 (0.0%) | 13 (0.0%) |
| Communication disorder | - | - | 1 (0.0%) | - | - | 7 (0.0%) | 8 (0.0%) |
| Contraindicated product administered | - | - | 1 (0.0%) | - | - | 4 (0.0%) | 5 (0.0%) |
| Conversion disorder | - | - | 1 (0.0%) | - | - | 6 (0.0%) | 7 (0.0%) |
| Coordination abnormal | - | - | 1 (0.0%) | - | - | 22 (0.1%) | 23 (0.1%) |
| Corneal light reflex test abnormal | - | - | 1 (0.0%) | - | - | - | 1 (0.0%) |
| Corneal reflex decreased | - | - | 1 (0.0%) | - | - | - | 1 (0.0%) |
| Cough | - | - | 6 (0.2%) | - | - | 38 (0.1%) | 44 (0.1%) |
| Cranial operation | - | - | 1 (0.0%) | - | - | - | 1 (0.0%) |
| Crepitations | - | - | 1 (0.0%) | - | - | 1 (0.0%) | 2 (0.0%) |
| Crying | - | - | 3 (0.1%) | - | - | 25 (0.1%) | 28 (0.1%) |
| CSF pressure increased | - | - | 1 (0.0%) | - | - | - | 1 (0.0%) |
| Cystitis | - | - | 1 (0.0%) | - | - | 9 (0.0%) | 10 (0.0%) |
| Cystostomy | - | - | 1 (0.0%) | - | - | - | 1 (0.0%) |
| Cytomegalovirus infection | - | - | 1 (0.0%) | - | - | - | 1 (0.0%) |
| Cytotoxic oedema | - | - | 1 (0.0%) | - | - | - | 1 (0.0%) |
| Decerebrate posture | - | - | 1 (0.0%) | - | - | 1 (0.0%) | 2 (0.0%) |
| Decorticate posture | - | - | 1 (0.0%) | - | - | - | 1 (0.0%) |
| Decreased eye contact | - | - | 1 (0.0%) | - | - | 1 (0.0%) | 2 (0.0%) |
| Deep vein thrombosis | - | - | 3 (0.1%) | - | - | 11 (0.0%) | 14 (0.0%) |
| Delayed recovery from anaesthesia | - | - | 10 (0.3%) | - | - | - | 10 (0.0%) |
| Delusion | - | - | 1 (0.0%) | - | 1 (0.2%) | 15 (0.1%) | 17 (0.0%) |
| Device programming error | - | - | 1 (0.0%) | - | - | - | 1 (0.0%) |
| Device related infection | - | - | 1 (0.0%) | - | - | 1 (0.0%) | 2 (0.0%) |
| Dilatation ventricular | - | - | 1 (0.0%) | - | - | - | 1 (0.0%) |
| Diplegia | - | - | 2 (0.1%) | - | - | 3 (0.0%) | 5 (0.0%) |
| Disability | - | - | 1 (0.0%) | - | - | 23 (0.1%) | 24 (0.1%) |
| Discouragement | - | - | 1 (0.0%) | - | - | - | 1 (0.0%) |
| Disease recurrence | - | - | 1 (0.0%) | - | - | - | 1 (0.0%) |
| Disseminated intravascular coagulation | - | - | 5 (0.1%) | - | - | 3 (0.0%) | 8 (0.0%) |
| Dose calculation error | - | - | 1 (0.0%) | - | - | - | 1 (0.0%) |
| Dreamy state | - | - | 1 (0.0%) | - | - | 3 (0.0%) | 4 (0.0%) |
| Drug abuser | - | - | 2 (0.1%) | - | - | 2 (0.0%) | 4 (0.0%) |
| Drug clearance decreased | - | - | 1 (0.0%) | - | - | - | 1 (0.0%) |
| Drug hypersensitivity | - | - | 5 (0.1%) | - | - | 13 (0.0%) | 18 (0.1%) |
| Drug intolerance | - | - | 1 (0.0%) | - | - | 24 (0.1%) | 25 (0.1%) |
| Drug level increased | - | - | 1 (0.0%) | - | 1 (0.2%) | 14 (0.0%) | 16 (0.0%) |
| Drug reaction with eosinophilia and systemic symptoms | - | - | 1 (0.0%) | - | - | - | 1 (0.0%) |
| Drug resistance | - | - | 3 (0.1%) | - | - | 2 (0.0%) | 5 (0.0%) |
| Drug use disorder | - | - | 2 (0.1%) | - | 1 (0.2%) | 2 (0.0%) | 5 (0.0%) |
| Duodenal ulcer | - | - | 1 (0.0%) | - | - | 1 (0.0%) | 2 (0.0%) |
| Duodenal vascular ectasia | - | - | 1 (0.0%) | - | - | - | 1 (0.0%) |
| Dyskinesia | - | - | 29 (0.8%) | - | 1 (0.2%) | 67 (0.2%) | 97 (0.3%) |
| Dyspepsia | - | - | 6 (0.2%) | - | - | 12 (0.0%) | 18 (0.1%) |
| Dysphonia | - | - | 3 (0.1%) | - | - | 7 (0.0%) | 10 (0.0%) |
| Dyspnoea exertional | - | - | 3 (0.1%) | - | - | 2 (0.0%) | 5 (0.0%) |
| Dysuria | - | - | 1 (0.0%) | - | - | 27 (0.1%) | 28 (0.1%) |
| Ejection fraction decreased | - | - | 1 (0.0%) | - | - | 1 (0.0%) | 2 (0.0%) |
| Elbow deformity | - | - | 1 (0.0%) | - | - | - | 1 (0.0%) |
| Electrocardiogram P wave abnormal | - | - | 1 (0.0%) | - | - | - | 1 (0.0%) |
| Electrocardiogram ST segment depression | - | - | 3 (0.1%) | - | - | - | 3 (0.0%) |
| Electrocardiogram ST segment elevation | - | - | 3 (0.1%) | - | - | - | 3 (0.0%) |
| Electrocardiogram T wave amplitude decreased | - | - | 1 (0.0%) | - | - | - | 1 (0.0%) |
| Electrocardiogram T wave inversion | - | - | 3 (0.1%) | - | - | 1 (0.0%) | 4 (0.0%) |
| Electrocardiogram U wave present | - | - | 1 (0.0%) | - | - | - | 1 (0.0%) |
| Electrocution | - | - | 2 (0.1%) | - | - | - | 2 (0.0%) |
| Electroencephalogram abnormal | - | - | 2 (0.1%) | - | - | 4 (0.0%) | 6 (0.0%) |
| Embolic stroke | - | - | 1 (0.0%) | - | - | - | 1 (0.0%) |
| Embolism | - | - | 1 (0.0%) | - | 1 (0.2%) | 1 (0.0%) | 3 (0.0%) |
| Embolism arterial | - | - | 1 (0.0%) | - | - | 1 (0.0%) | 2 (0.0%) |
| Emotional disorder | - | - | 1 (0.0%) | - | - | 20 (0.1%) | 21 (0.1%) |
| End-tidal CO2 decreased | - | - | 1 (0.0%) | - | - | - | 1 (0.0%) |
| End-tidal CO2 increased | - | - | 2 (0.1%) | - | - | - | 2 (0.0%) |
| Endocarditis | - | - | 1 (0.0%) | - | - | 2 (0.0%) | 3 (0.0%) |
| Endocarditis bacterial | - | - | 1 (0.0%) | - | - | - | 1 (0.0%) |
| Endotracheal intubation | - | - | 3 (0.1%) | - | 1 (0.2%) | - | 4 (0.0%) |
| Endotracheal intubation complication | - | - | 1 (0.0%) | - | - | - | 1 (0.0%) |
| Enophthalmos | - | - | 1 (0.0%) | - | - | - | 1 (0.0%) |
| Enterococcal sepsis | - | - | 1 (0.0%) | - | - | - | 1 (0.0%) |
| Enterocolitis | - | - | 1 (0.0%) | - | - | - | 1 (0.0%) |
| Epidural lipomatosis | - | - | 1 (0.0%) | - | - | - | 1 (0.0%) |
| Epigastric discomfort | - | - | 1 (0.0%) | - | - | 3 (0.0%) | 4 (0.0%) |
| Epilepsy | - | - | 6 (0.2%) | 1 (0.3%) | - | 11 (0.0%) | 18 (0.1%) |
| Epistaxis | - | - | 3 (0.1%) | - | - | 6 (0.0%) | 9 (0.0%) |
| Erythema | - | - | 12 (0.3%) | - | - | 32 (0.1%) | 44 (0.1%) |
| Extensor plantar response | - | - | 2 (0.1%) | - | - | 1 (0.0%) | 3 (0.0%) |
| Extrapyramidal disorder | - | - | 8 (0.2%) | - | - | 7 (0.0%) | 15 (0.0%) |
| Extubation | - | - | 1 (0.0%) | - | - | - | 1 (0.0%) |
| Eye movement disorder | - | - | 7 (0.2%) | - | - | 3 (0.0%) | 10 (0.0%) |
| Eyelid function disorder | - | - | 1 (0.0%) | - | - | 2 (0.0%) | 3 (0.0%) |
| Eyelid ptosis | - | - | 2 (0.1%) | - | - | - | 2 (0.0%) |
| Face lift | - | - | 1 (0.0%) | - | - | - | 1 (0.0%) |
| Facial paresis | - | - | 1 (0.0%) | - | - | - | 1 (0.0%) |
| Facial spasm | - | - | 1 (0.0%) | - | - | - | 1 (0.0%) |
| Faeces discoloured | - | - | 1 (0.0%) | - | - | 1 (0.0%) | 2 (0.0%) |
| Fear | - | - | 3 (0.1%) | - | 1 (0.2%) | 15 (0.1%) | 19 (0.1%) |
| Febrile convulsion | - | - | 2 (0.1%) | - | - | - | 2 (0.0%) |
| Feeling cold | - | - | 3 (0.1%) | - | 1 (0.2%) | 51 (0.2%) | 55 (0.2%) |
| Feeling drunk | - | - | 1 (0.0%) | - | - | 10 (0.0%) | 11 (0.0%) |
| Feeling hot | - | - | 1 (0.0%) | - | - | 35 (0.1%) | 36 (0.1%) |
| Female genital tract fistula | - | - | 7 (0.2%) | - | - | - | 7 (0.0%) |
| Finger deformity | - | - | 1 (0.0%) | - | - | - | 1 (0.0%) |
| Flail chest | - | - | 1 (0.0%) | - | - | - | 1 (0.0%) |
| Flashback | - | - | 1 (0.0%) | - | - | 1 (0.0%) | 2 (0.0%) |
| Foot fracture | - | - | 1 (0.0%) | - | - | 11 (0.0%) | 12 (0.0%) |
| Frequent bowel movements | - | - | 7 (0.2%) | - | - | 3 (0.0%) | 10 (0.0%) |
| Frustration tolerance decreased | - | - | 1 (0.0%) | - | - | 5 (0.0%) | 6 (0.0%) |
| Gastrointestinal hypomotility | - | - | 1 (0.0%) | - | - | - | 1 (0.0%) |
| Glomerulonephritis proliferative | - | - | 1 (0.0%) | - | - | - | 1 (0.0%) |
| Glossoptosis | - | - | 1 (0.0%) | - | - | - | 1 (0.0%) |
| Grip strength decreased | - | - | 1 (0.0%) | - | - | 2 (0.0%) | 3 (0.0%) |
| Groin pain | - | - | 1 (0.0%) | - | - | 3 (0.0%) | 4 (0.0%) |
| Grunting | - | - | 1 (0.0%) | - | - | - | 1 (0.0%) |
| Guillain-Barre syndrome | - | - | 1 (0.0%) | - | - | 1 (0.0%) | 2 (0.0%) |
| Haematochezia | - | - | 7 (0.2%) | - | - | 5 (0.0%) | 12 (0.0%) |
| Haematuria | - | - | 2 (0.1%) | - | - | 4 (0.0%) | 6 (0.0%) |
| Haematuria traumatic | - | - | 1 (0.0%) | - | - | - | 1 (0.0%) |
| Haemodynamic instability | - | - | 3 (0.1%) | - | - | 1 (0.0%) | 4 (0.0%) |
| Haemoglobin decreased | - | - | 1 (0.0%) | - | 1 (0.2%) | 8 (0.0%) | 10 (0.0%) |
| Haemoptysis | - | - | 3 (0.1%) | 1 (0.3%) | - | 6 (0.0%) | 10 (0.0%) |
| Haemorrhage | - | - | 4 (0.1%) | 2 (0.5%) | - | 9 (0.0%) | 15 (0.0%) |
| Hallucination, auditory | - | - | 1 (0.0%) | - | - | 8 (0.0%) | 9 (0.0%) |
| Head discomfort | - | - | 2 (0.1%) | 1 (0.3%) | - | 15 (0.1%) | 18 (0.1%) |
| Head titubation | - | - | 1 (0.0%) | - | - | 1 (0.0%) | 2 (0.0%) |
| Heart rate irregular | - | - | 2 (0.1%) | - | - | 6 (0.0%) | 8 (0.0%) |
| Heart sounds abnormal | - | - | 1 (0.0%) | - | - | - | 1 (0.0%) |
| Hemianopia | - | - | 1 (0.0%) | - | - | - | 1 (0.0%) |
| Hemiparesis | - | - | 4 (0.1%) | - | - | 12 (0.0%) | 16 (0.0%) |
| Hemiplegia | - | - | 4 (0.1%) | - | - | 8 (0.0%) | 12 (0.0%) |
| Hemiplegic migraine | - | - | 2 (0.1%) | - | - | - | 2 (0.0%) |
| Heparin-induced thrombocytopenia | - | - | 1 (0.0%) | - | - | - | 1 (0.0%) |
| Hepatic function abnormal | - | - | 2 (0.1%) | - | - | 9 (0.0%) | 11 (0.0%) |
| Hepatitis | - | - | 1 (0.0%) | - | - | 2 (0.0%) | 3 (0.0%) |
| Hepatitis A | - | - | 1 (0.0%) | - | - | 1 (0.0%) | 2 (0.0%) |
| Hepatitis B | - | - | 1 (0.0%) | - | - | - | 1 (0.0%) |
| Hepatitis C | - | - | 1 (0.0%) | - | - | - | 1 (0.0%) |
| Hepatitis fulminant | - | - | 2 (0.1%) | - | - | 1 (0.0%) | 3 (0.0%) |
| Herpes simplex | - | - | 1 (0.0%) | - | - | - | 1 (0.0%) |
| Herpes virus infection | - | - | 2 (0.1%) | - | - | - | 2 (0.0%) |
| Herpes zoster | - | - | 1 (0.0%) | - | - | 13 (0.0%) | 14 (0.0%) |
| Hiccups | - | - | 1 (0.0%) | - | - | 6 (0.0%) | 7 (0.0%) |
| Hip arthroplasty | - | - | 1 (0.0%) | - | - | 6 (0.0%) | 7 (0.0%) |
| Horner's syndrome | - | - | 7 (0.2%) | - | - | - | 7 (0.0%) |
| Hunger | - | - | 1 (0.0%) | - | - | - | 1 (0.0%) |
| Hydrocephalus | - | - | 3 (0.1%) | 1 (0.3%) | - | 2 (0.0%) | 6 (0.0%) |
| Hypercalcaemia | - | - | 1 (0.0%) | - | - | 5 (0.0%) | 6 (0.0%) |
| Hypercapnia | - | - | 6 (0.2%) | - | - | 11 (0.0%) | 17 (0.0%) |
| Hyperchlorhydria | - | - | 1 (0.0%) | - | - | - | 1 (0.0%) |
| Hyperdynamic left ventricle | - | - | 1 (0.0%) | - | - | - | 1 (0.0%) |
| Hyperglycaemia | - | - | 3 (0.1%) | - | - | 6 (0.0%) | 9 (0.0%) |
| Hyperkinesia | - | - | 2 (0.1%) | - | - | 2 (0.0%) | 4 (0.0%) |
| Hyperlactacidaemia | - | - | 1 (0.0%) | - | - | 1 (0.0%) | 2 (0.0%) |
| Hypernatraemia | - | - | 1 (0.0%) | - | - | 1 (0.0%) | 2 (0.0%) |
| Hyperreflexia | - | - | 11 (0.3%) | - | - | 7 (0.0%) | 18 (0.1%) |
| Hypersensitivity | - | - | 5 (0.1%) | - | - | 20 (0.1%) | 25 (0.1%) |
| Hypersensitivity vasculitis | - | - | 1 (0.0%) | - | - | - | 1 (0.0%) |
| Hypersomnia | - | - | 1 (0.0%) | - | 1 (0.2%) | 108 (0.4%) | 110 (0.3%) |
| Hypertensive crisis | - | - | 2 (0.1%) | - | 1 (0.2%) | 2 (0.0%) | 5 (0.0%) |
| Hypertensive emergency | - | - | 1 (0.0%) | - | - | - | 1 (0.0%) |
| Hyperthermia | - | - | 10 (0.3%) | - | - | 11 (0.0%) | 21 (0.1%) |
| Hyperthermia malignant | - | - | 5 (0.1%) | - | - | - | 5 (0.0%) |
| Hypervolaemia | - | - | 3 (0.1%) | - | - | 2 (0.0%) | 5 (0.0%) |
| Hypocalcaemia | - | - | 5 (0.1%) | - | - | 3 (0.0%) | 8 (0.0%) |
| Hypochromic anaemia | - | - | 1 (0.0%) | - | - | - | 1 (0.0%) |
| Hypoglossal nerve disorder | - | - | 1 (0.0%) | - | - | - | 1 (0.0%) |
| Hypoglossal nerve paralysis | - | - | 1 (0.0%) | - | - | - | 1 (0.0%) |
| Hypoglycaemia | - | - | 4 (0.1%) | - | - | 16 (0.1%) | 20 (0.1%) |
| Hypomagnesaemia | - | - | 1 (0.0%) | - | - | - | 1 (0.0%) |
| Hypophosphataemia | - | - | 1 (0.0%) | - | - | - | 1 (0.0%) |
| Hyporesponsive to stimuli | - | - | 2 (0.1%) | - | - | 1 (0.0%) | 3 (0.0%) |
| Hypothyroidism | - | - | 1 (0.0%) | - | - | 9 (0.0%) | 10 (0.0%) |
| Hypoventilation | - | - | 4 (0.1%) | - | - | 23 (0.1%) | 27 (0.1%) |
| Hypovolaemic shock | - | - | 2 (0.1%) | - | - | - | 2 (0.0%) |
| Hypoxic-ischaemic encephalopathy | - | - | 10 (0.3%) | - | - | 14 (0.0%) | 24 (0.1%) |
| IIIrd nerve paralysis | - | - | 1 (0.0%) | - | - | - | 1 (0.0%) |
| Ileus | - | - | 1 (0.0%) | - | 1 (0.2%) | 7 (0.0%) | 9 (0.0%) |
| Immunodeficiency | - | - | 1 (0.0%) | - | - | - | 1 (0.0%) |
| Impaired gastric emptying | - | - | 1 (0.0%) | - | - | 5 (0.0%) | 6 (0.0%) |
| Impaired work ability | - | - | 1 (0.0%) | - | - | 7 (0.0%) | 8 (0.0%) |
| Implant site pain | - | - | 1 (0.0%) | - | - | 1 (0.0%) | 2 (0.0%) |
| Implant site swelling | - | - | 1 (0.0%) | - | - | 1 (0.0%) | 2 (0.0%) |
| Imprisonment | - | - | 2 (0.1%) | - | - | - | 2 (0.0%) |
| Inadequate analgesia | - | - | 2 (0.1%) | - | - | 65 (0.2%) | 67 (0.2%) |
| Incorrect drug administration rate | - | - | 2 (0.1%) | - | - | 8 (0.0%) | 10 (0.0%) |
| Incorrect product administration duration | - | - | 1 (0.0%) | - | - | 50 (0.2%) | 51 (0.1%) |
| Increased bronchial secretion | - | - | 1 (0.0%) | - | - | 1 (0.0%) | 2 (0.0%) |
| Increased upper airway secretion | - | - | 1 (0.0%) | - | - | 2 (0.0%) | 3 (0.0%) |
| Infantile apnoea | - | - | 1 (0.0%) | - | - | - | 1 (0.0%) |
| Influenza like illness | - | - | 1 (0.0%) | - | - | 19 (0.1%) | 20 (0.1%) |
| Infusion related reaction | - | - | 7 (0.2%) | - | - | 1 (0.0%) | 8 (0.0%) |
| Infusion site discharge | - | - | 1 (0.0%) | - | - | - | 1 (0.0%) |
| Infusion site extravasation | - | - | 2 (0.1%) | - | - | - | 2 (0.0%) |
| Injection site extravasation | - | - | 1 (0.0%) | - | - | - | 1 (0.0%) |
| Injection site hypoaesthesia | - | - | 1 (0.0%) | - | - | - | 1 (0.0%) |
| Injection site pain | - | - | 4 (0.1%) | - | - | 1 (0.0%) | 5 (0.0%) |
| Injury | - | - | 1 (0.0%) | - | - | 26 (0.1%) | 27 (0.1%) |
| Intensive care unit acquired weakness | - | - | 2 (0.1%) | - | - | - | 2 (0.0%) |
| Interstitial lung disease | - | - | 1 (0.0%) | - | 1 (0.2%) | 5 (0.0%) | 7 (0.0%) |
| Intervertebral disc protrusion | - | - | 1 (0.0%) | - | - | 14 (0.0%) | 15 (0.0%) |
| Intestinal ischaemia | - | - | 1 (0.0%) | - | - | 1 (0.0%) | 2 (0.0%) |
| Intracranial pressure increased | - | - | 3 (0.1%) | - | - | 3 (0.0%) | 6 (0.0%) |
| Intraventricular haemorrhage | - | - | 2 (0.1%) | - | - | - | 2 (0.0%) |
| Irregular breathing | - | - | 2 (0.1%) | - | 1 (0.2%) | 3 (0.0%) | 6 (0.0%) |
| Judgement impaired | - | - | 3 (0.1%) | - | - | 2 (0.0%) | 5 (0.0%) |
| Knee arthroplasty | - | - | 1 (0.0%) | - | - | 3 (0.0%) | 4 (0.0%) |
| Laboratory test abnormal | - | - | 1 (0.0%) | - | - | 3 (0.0%) | 4 (0.0%) |
| Lactic acidosis | - | - | 1 (0.0%) | 1 (0.3%) | - | - | 2 (0.0%) |
| Language disorder | - | - | 1 (0.0%) | - | - | 11 (0.0%) | 12 (0.0%) |
| Laryngeal discomfort | - | - | 1 (0.0%) | - | - | - | 1 (0.0%) |
| Laryngeal oedema | - | - | 1 (0.0%) | - | - | - | 1 (0.0%) |
| Laryngospasm | - | - | 3 (0.1%) | - | - | - | 3 (0.0%) |
| Left atrial dilatation | - | - | 1 (0.0%) | - | - | - | 1 (0.0%) |
| Left ventricular failure | - | - | 1 (0.0%) | - | - | - | 1 (0.0%) |
| Leukocytosis | - | - | 3 (0.1%) | 1 (0.3%) | - | 4 (0.0%) | 8 (0.0%) |
| Ligament sprain | - | - | 1 (0.0%) | - | - | 5 (0.0%) | 6 (0.0%) |
| Limb discomfort | - | - | 1 (0.0%) | - | - | 8 (0.0%) | 9 (0.0%) |
| Live birth | - | - | 1 (0.0%) | - | - | - | 1 (0.0%) |
| Liver disorder | - | - | 1 (0.0%) | - | - | 13 (0.0%) | 14 (0.0%) |
| Liver function test abnormal | - | - | 2 (0.1%) | - | - | 11 (0.0%) | 13 (0.0%) |
| Locked-in syndrome | - | - | 1 (0.0%) | - | - | 1 (0.0%) | 2 (0.0%) |
| Lung consolidation | - | - | 1 (0.0%) | - | - | - | 1 (0.0%) |
| Lung disorder | - | - | 1 (0.0%) | - | - | 15 (0.1%) | 16 (0.0%) |
| Lung infiltration | - | - | 2 (0.1%) | - | - | 7 (0.0%) | 9 (0.0%) |
| Lymphadenopathy | - | - | 1 (0.0%) | - | - | 3 (0.0%) | 4 (0.0%) |
| Macular degeneration | - | - | 7 (0.2%) | - | - | 1 (0.0%) | 8 (0.0%) |
| Malabsorption | - | - | 1 (0.0%) | - | - | 1 (0.0%) | 2 (0.0%) |
| Malignant hypertension | - | - | 1 (0.0%) | - | - | 1 (0.0%) | 2 (0.0%) |
| Mallory-Weiss syndrome | - | - | 1 (0.0%) | - | - | 3 (0.0%) | 4 (0.0%) |
| Malnutrition | - | - | 1 (0.0%) | - | - | 11 (0.0%) | 12 (0.0%) |
| Maternal exposure during breast feeding | - | - | 2 (0.1%) | - | - | - | 2 (0.0%) |
| Maternal exposure during delivery | - | - | 3 (0.1%) | - | - | - | 3 (0.0%) |
| Maternal exposure during pregnancy | - | - | 7 (0.2%) | - | - | 4 (0.0%) | 11 (0.0%) |
| Mechanical ventilation | - | - | 3 (0.1%) | - | - | 1 (0.0%) | 4 (0.0%) |
| Medical device discomfort | - | - | 1 (0.0%) | - | - | - | 1 (0.0%) |
| Medical device site haemorrhage | - | - | 1 (0.0%) | - | - | - | 1 (0.0%) |
| Medication error | - | - | 2 (0.1%) | 3 (0.8%) | - | 77 (0.3%) | 82 (0.2%) |
| Mental disorder | - | - | 1 (0.0%) | 1 (0.3%) | - | 16 (0.1%) | 18 (0.1%) |
| Metabolic acidosis | - | - | 10 (0.3%) | - | - | 9 (0.0%) | 19 (0.1%) |
| Metastases to adrenals | - | - | 1 (0.0%) | - | - | 1 (0.0%) | 2 (0.0%) |
| Metastases to kidney | - | - | 1 (0.0%) | - | - | - | 1 (0.0%) |
| Metastases to lymph nodes | - | - | 1 (0.0%) | - | - | 1 (0.0%) | 2 (0.0%) |
| Middle cerebral artery stroke | - | - | 1 (0.0%) | - | - | 1 (0.0%) | 2 (0.0%) |
| Mitral valve calcification | - | - | 1 (0.0%) | - | - | - | 1 (0.0%) |
| Mitral valve incompetence | - | - | 1 (0.0%) | - | - | 2 (0.0%) | 3 (0.0%) |
| Monoparesis | - | - | 3 (0.1%) | - | - | 1 (0.0%) | 4 (0.0%) |
| Mouth haemorrhage | - | - | 1 (0.0%) | - | - | 3 (0.0%) | 4 (0.0%) |
| Movement disorder | - | - | 9 (0.2%) | - | 1 (0.2%) | 19 (0.1%) | 29 (0.1%) |
| Multiple organ dysfunction syndrome | - | - | 6 (0.2%) | 1 (0.3%) | 1 (0.2%) | 12 (0.0%) | 20 (0.1%) |
| Multiple system atrophy | - | - | 1 (0.0%) | - | - | - | 1 (0.0%) |
| Muscle contractions involuntary | - | - | 8 (0.2%) | - | - | 6 (0.0%) | 14 (0.0%) |
| Muscle contracture | - | - | 1 (0.0%) | - | - | 3 (0.0%) | 4 (0.0%) |
| Muscle rigidity | - | - | 26 (0.7%) | - | - | 10 (0.0%) | 36 (0.1%) |
| Muscle spasticity | - | - | 4 (0.1%) | - | - | 8 (0.0%) | 12 (0.0%) |
| Muscle tightness | - | - | 1 (0.0%) | - | - | 10 (0.0%) | 11 (0.0%) |
| Musculoskeletal chest pain | - | - | 2 (0.1%) | - | - | 11 (0.0%) | 13 (0.0%) |
| Musculoskeletal discomfort | - | - | 1 (0.0%) | - | - | 1 (0.0%) | 2 (0.0%) |
| Musculoskeletal disorder | - | - | 1 (0.0%) | - | - | 2 (0.0%) | 3 (0.0%) |
| Musculoskeletal pain | - | - | 1 (0.0%) | - | - | 2 (0.0%) | 3 (0.0%) |
| Musculoskeletal stiffness | - | - | 9 (0.2%) | - | 1 (0.2%) | 24 (0.1%) | 34 (0.1%) |
| Mutism | - | - | 1 (0.0%) | - | - | - | 1 (0.0%) |
| Myasthenia gravis crisis | - | - | 2 (0.1%) | - | - | - | 2 (0.0%) |
| Mydriasis | - | - | 9 (0.2%) | 1 (0.3%) | - | 14 (0.0%) | 24 (0.1%) |
| Myoclonic epilepsy | - | - | 2 (0.1%) | - | - | - | 2 (0.0%) |
| Myoglobin urine present | - | - | 2 (0.1%) | - | - | - | 2 (0.0%) |
| Myotonia | - | - | 1 (0.0%) | - | - | - | 1 (0.0%) |
| Nasopharyngitis | - | - | 3 (0.1%) | - | - | 11 (0.0%) | 14 (0.0%) |
| Near death experience | - | - | 1 (0.0%) | - | - | 7 (0.0%) | 8 (0.0%) |
| Neck pain | - | - | 7 (0.2%) | - | - | 18 (0.1%) | 25 (0.1%) |
| Neonatal respiratory depression | - | - | 2 (0.1%) | - | - | - | 2 (0.0%) |
| Nephrolithiasis | - | - | 1 (0.0%) | - | - | 11 (0.0%) | 12 (0.0%) |
| Neuralgia | - | - | 1 (0.0%) | 2 (0.5%) | - | 26 (0.1%) | 29 (0.1%) |
| Neuroleptic malignant syndrome | - | - | 16 (0.4%) | - | - | 6 (0.0%) | 22 (0.1%) |
| Neurological decompensation | - | - | 3 (0.1%) | - | - | - | 3 (0.0%) |
| Neurological symptom | - | - | 2 (0.1%) | - | 1 (0.2%) | 7 (0.0%) | 10 (0.0%) |
| Neuromuscular block prolonged | - | - | 2 (0.1%) | - | - | - | 2 (0.0%) |
| Neuropathy peripheral | - | - | 1 (0.0%) | - | - | 54 (0.2%) | 55 (0.2%) |
| Non-small cell lung cancer stage I | - | - | 1 (0.0%) | - | - | - | 1 (0.0%) |
| Nystagmus | - | - | 5 (0.1%) | - | - | 2 (0.0%) | 7 (0.0%) |
| Obstructive airways disorder | - | - | 5 (0.1%) | - | - | 5 (0.0%) | 10 (0.0%) |
| Obstructive sleep apnoea syndrome | - | - | 1 (0.0%) | - | - | 1 (0.0%) | 2 (0.0%) |
| Occipital neuralgia | - | - | 1 (0.0%) | - | - | - | 1 (0.0%) |
| Ocular hyperaemia | - | - | 1 (0.0%) | - | - | - | 1 (0.0%) |
| Ocular hypertension | - | - | 1 (0.0%) | - | - | - | 1 (0.0%) |
| Ocular icterus | - | - | 1 (0.0%) | - | - | - | 1 (0.0%) |
| Oculogyric crisis | - | - | 1 (0.0%) | - | - | 1 (0.0%) | 2 (0.0%) |
| Ophthalmic migraine | - | - | 1 (0.0%) | - | - | - | 1 (0.0%) |
| Ophthalmoplegia | - | - | 1 (0.0%) | - | - | - | 1 (0.0%) |
| Opisthotonus | - | - | 1 (0.0%) | - | - | - | 1 (0.0%) |
| Optic nerve disorder | - | - | 1 (0.0%) | - | - | - | 1 (0.0%) |
| Optic neuritis | - | - | 1 (0.0%) | - | - | 1 (0.0%) | 2 (0.0%) |
| Oral candidiasis | - | - | 7 (0.2%) | - | - | 2 (0.0%) | 9 (0.0%) |
| Oral discharge | - | - | 1 (0.0%) | - | - | - | 1 (0.0%) |
| Oropharyngeal pain | - | - | 1 (0.0%) | - | - | 16 (0.1%) | 17 (0.0%) |
| Orthostatic intolerance | - | - | 1 (0.0%) | - | - | - | 1 (0.0%) |
| Otorrhoea | - | - | 1 (0.0%) | - | - | - | 1 (0.0%) |
| Overweight | - | - | 1 (0.0%) | - | - | 1 (0.0%) | 2 (0.0%) |
| Oxygen consumption decreased | - | - | 3 (0.1%) | - | - | - | 3 (0.0%) |
| Oxygen saturation abnormal | - | - | 3 (0.1%) | - | - | 2 (0.0%) | 5 (0.0%) |
| Pain of skin | - | - | 1 (0.0%) | - | - | 2 (0.0%) | 3 (0.0%) |
| Panic reaction | - | - | 1 (0.0%) | - | - | 5 (0.0%) | 6 (0.0%) |
| Papilloedema | - | - | 1 (0.0%) | - | - | - | 1 (0.0%) |
| Paraesthesia oral | - | - | 8 (0.2%) | - | - | 5 (0.0%) | 13 (0.0%) |
| Paranoia | - | - | 1 (0.0%) | - | - | 9 (0.0%) | 10 (0.0%) |
| Paresis | - | - | 1 (0.0%) | - | - | 4 (0.0%) | 5 (0.0%) |
| Parkinsonism | - | - | 1 (0.0%) | - | - | 6 (0.0%) | 7 (0.0%) |
| Parosmia | - | - | 2 (0.1%) | - | - | 10 (0.0%) | 12 (0.0%) |
| Partial seizures | - | - | 2 (0.1%) | - | - | 2 (0.0%) | 4 (0.0%) |
| Patent ductus arteriosus | - | - | 1 (0.0%) | - | - | - | 1 (0.0%) |
| PCO2 increased | - | - | 1 (0.0%) | - | - | 2 (0.0%) | 3 (0.0%) |
| Pericardial effusion | - | - | 1 (0.0%) | - | - | 3 (0.0%) | 4 (0.0%) |
| Pericarditis uraemic | - | - | 1 (0.0%) | - | - | - | 1 (0.0%) |
| Periorbital oedema | - | - | 1 (0.0%) | - | - | - | 1 (0.0%) |
| Peripheral coldness | - | - | 3 (0.1%) | - | - | 8 (0.0%) | 11 (0.0%) |
| Peripheral nerve palsy | - | - | 1 (0.0%) | - | - | - | 1 (0.0%) |
| Peripheral swelling | - | - | 1 (0.0%) | - | - | 35 (0.1%) | 36 (0.1%) |
| Petit mal epilepsy | - | - | 1 (0.0%) | - | 1 (0.2%) | 5 (0.0%) | 7 (0.0%) |
| Pharyngeal haemorrhage | - | - | 1 (0.0%) | - | - | - | 1 (0.0%) |
| Pharyngeal mass | - | - | 1 (0.0%) | - | - | - | 1 (0.0%) |
| Phrenic nerve paralysis | - | - | 1 (0.0%) | - | - | - | 1 (0.0%) |
| Physical examination abnormal | - | - | 1 (0.0%) | - | - | - | 1 (0.0%) |
| Platelet dysfunction | - | - | 1 (0.0%) | - | - | - | 1 (0.0%) |
| Pleural effusion | - | - | 6 (0.2%) | 1 (0.3%) | 1 (0.2%) | 14 (0.0%) | 22 (0.1%) |
| Pneumocephalus | - | - | 1 (0.0%) | - | - | - | 1 (0.0%) |
| Pneumonia staphylococcal | - | - | 1 (0.0%) | - | - | 4 (0.0%) | 5 (0.0%) |
| Pneumonitis aspiration | - | - | 1 (0.0%) | - | - | 2 (0.0%) | 3 (0.0%) |
| Pneumothorax | - | - | 4 (0.1%) | - | 1 (0.2%) | 4 (0.0%) | 9 (0.0%) |
| PO2 decreased | - | - | 2 (0.1%) | - | - | 1 (0.0%) | 3 (0.0%) |
| Poisoning | - | - | 5 (0.1%) | - | - | 7 (0.0%) | 12 (0.0%) |
| Pollakiuria | - | - | 2 (0.1%) | - | - | 14 (0.0%) | 16 (0.0%) |
| Post-traumatic stress disorder | - | - | 1 (0.0%) | - | - | 13 (0.0%) | 14 (0.0%) |
| Post procedural complication | - | - | 4 (0.1%) | - | - | 2 (0.0%) | 6 (0.0%) |
| Post procedural fever | - | - | 1 (0.0%) | - | - | - | 1 (0.0%) |
| Posterior reversible encephalopathy syndrome | - | - | 3 (0.1%) | - | - | 3 (0.0%) | 6 (0.0%) |
| Posterior tibial tendon dysfunction | - | - | 1 (0.0%) | - | - | - | 1 (0.0%) |
| Postoperative delirium | - | - | 3 (0.1%) | - | - | - | 3 (0.0%) |
| Postpartum haemorrhage | - | - | 1 (0.0%) | - | - | - | 1 (0.0%) |
| Postresuscitation encephalopathy | - | - | 1 (0.0%) | - | - | - | 1 (0.0%) |
| Potentiating drug interaction | - | - | 5 (0.1%) | - | - | 7 (0.0%) | 12 (0.0%) |
| Premature delivery | - | - | 2 (0.1%) | - | - | - | 2 (0.0%) |
| Prescribed overdose | - | - | 1 (0.0%) | 1 (0.3%) | 1 (0.2%) | 9 (0.0%) | 12 (0.0%) |
| Procedural nausea | - | - | 1 (0.0%) | - | - | - | 1 (0.0%) |
| Procedural pain | - | - | 10 (0.3%) | - | 1 (0.2%) | 4 (0.0%) | 15 (0.0%) |
| Procedural vomiting | - | - | 2 (0.1%) | - | - | - | 2 (0.0%) |
| Proctitis | - | - | 7 (0.2%) | - | - | - | 7 (0.0%) |
| Product label confusion | - | - | 1 (0.0%) | - | - | 2 (0.0%) | 3 (0.0%) |
| Product monitoring error | - | - | 1 (0.0%) | - | - | - | 1 (0.0%) |
| Productive cough | - | - | 1 (0.0%) | - | - | 3 (0.0%) | 4 (0.0%) |
| Propofol infusion syndrome | - | - | 1 (0.0%) | - | - | - | 1 (0.0%) |
| Prothrombin time prolonged | - | - | 1 (0.0%) | - | - | 2 (0.0%) | 3 (0.0%) |
| Pseudomembranous colitis | - | - | 1 (0.0%) | - | - | - | 1 (0.0%) |
| Pseudomonal sepsis | - | - | 1 (0.0%) | - | - | 1 (0.0%) | 2 (0.0%) |
| Psychogenic seizure | - | - | 1 (0.0%) | - | - | 1 (0.0%) | 2 (0.0%) |
| Psychotic disorder | - | - | 1 (0.0%) | - | - | 17 (0.1%) | 18 (0.1%) |
| Pubic pain | - | - | 1 (0.0%) | - | - | - | 1 (0.0%) |
| Pulmonary arterial pressure increased | - | - | 1 (0.0%) | - | - | - | 1 (0.0%) |
| Pulmonary cavitation | - | - | 1 (0.0%) | - | - | - | 1 (0.0%) |
| Pulmonary congestion | - | - | 1 (0.0%) | - | - | 6 (0.0%) | 7 (0.0%) |
| Pulmonary embolism | - | - | 1 (0.0%) | - | 1 (0.2%) | 11 (0.0%) | 13 (0.0%) |
| Pulmonary granuloma | - | - | 2 (0.1%) | - | - | - | 2 (0.0%) |
| Pulmonary haemorrhage | - | - | 1 (0.0%) | - | - | 1 (0.0%) | 2 (0.0%) |
| Pulmonary hypertension | - | - | 1 (0.0%) | - | - | 1 (0.0%) | 2 (0.0%) |
| Pulmonary hypertensive crisis | - | - | 1 (0.0%) | - | - | - | 1 (0.0%) |
| Pulmonary infarction | - | - | 1 (0.0%) | - | - | 1 (0.0%) | 2 (0.0%) |
| Pulmonary oedema | - | - | 6 (0.2%) | 1 (0.3%) | - | 38 (0.1%) | 45 (0.1%) |
| Pulmonary valve incompetence | - | - | 1 (0.0%) | - | - | - | 1 (0.0%) |
| Pulse abnormal | - | - | 2 (0.1%) | - | - | 2 (0.0%) | 4 (0.0%) |
| Pulse absent | - | - | 2 (0.1%) | - | - | 4 (0.0%) | 6 (0.0%) |
| Pulseless electrical activity | - | - | 2 (0.1%) | - | - | - | 2 (0.0%) |
| Pupil fixed | - | - | 1 (0.0%) | 1 (0.3%) | - | 2 (0.0%) | 4 (0.0%) |
| Pupillary reflex impaired | - | - | 2 (0.1%) | 1 (0.3%) | - | 4 (0.0%) | 7 (0.0%) |
| Radiculopathy | - | - | 7 (0.2%) | - | 1 (0.2%) | 3 (0.0%) | 11 (0.0%) |
| Rales | - | - | 2 (0.1%) | - | - | 2 (0.0%) | 4 (0.0%) |
| Rash erythematous | - | - | 1 (0.0%) | - | - | 5 (0.0%) | 6 (0.0%) |
| Rash maculo-papular | - | - | 1 (0.0%) | - | - | - | 1 (0.0%) |
| Rectal cancer | - | - | 1 (0.0%) | - | - | 6 (0.0%) | 7 (0.0%) |
| Recurrence of neuromuscular blockade | - | - | 1 (0.0%) | - | - | - | 1 (0.0%) |
| Renal disorder | - | - | 2 (0.1%) | - | - | 8 (0.0%) | 10 (0.0%) |
| Respiration abnormal | - | - | 5 (0.1%) | - | 1 (0.2%) | 2 (0.0%) | 8 (0.0%) |
| Respiratory acidosis | - | - | 6 (0.2%) | - | - | 13 (0.0%) | 19 (0.1%) |
| Respiratory disorder | - | - | 5 (0.1%) | - | - | 20 (0.1%) | 25 (0.1%) |
| Respiratory paralysis | - | - | 1 (0.0%) | - | - | - | 1 (0.0%) |
| Respiratory tract infection | - | - | 1 (0.0%) | - | - | 3 (0.0%) | 4 (0.0%) |
| Retinal degeneration | - | - | 1 (0.0%) | - | - | - | 1 (0.0%) |
| Retrograde amnesia | - | - | 4 (0.1%) | - | - | - | 4 (0.0%) |
| Retroperitoneal lymphadenopathy | - | - | 2 (0.1%) | - | 1 (0.2%) | - | 3 (0.0%) |
| Reversal of sedation | - | - | 1 (0.0%) | - | - | - | 1 (0.0%) |
| Salivary hypersecretion | - | - | 2 (0.1%) | - | - | 5 (0.0%) | 7 (0.0%) |
| Screaming | - | - | 1 (0.0%) | - | - | 6 (0.0%) | 7 (0.0%) |
| Sedation complication | - | - | 5 (0.1%) | - | 1 (0.2%) | 20 (0.1%) | 26 (0.1%) |
| Seizure cluster | - | - | 1 (0.0%) | - | - | - | 1 (0.0%) |
| Seizure like phenomena | - | - | 1 (0.0%) | - | - | - | 1 (0.0%) |
| Sensorimotor disorder | - | - | 1 (0.0%) | - | - | - | 1 (0.0%) |
| Sensory disturbance | - | - | 4 (0.1%) | - | - | 19 (0.1%) | 23 (0.1%) |
| Sensory loss | - | - | 2 (0.1%) | - | - | 14 (0.0%) | 16 (0.0%) |
| Shock | - | - | 1 (0.0%) | - | - | 3 (0.0%) | 4 (0.0%) |
| Shock haemorrhagic | - | - | 1 (0.0%) | - | - | - | 1 (0.0%) |
| Sinus bradycardia | - | - | 1 (0.0%) | 1 (0.3%) | - | 5 (0.0%) | 7 (0.0%) |
| Sinus tachycardia | - | - | 6 (0.2%) | - | - | 11 (0.0%) | 17 (0.0%) |
| Skin discolouration | - | - | 2 (0.1%) | - | - | 7 (0.0%) | 9 (0.0%) |
| Skin exfoliation | - | - | 1 (0.0%) | - | - | 5 (0.0%) | 6 (0.0%) |
| Slow response to stimuli | - | - | 1 (0.0%) | - | 2 (0.4%) | 7 (0.0%) | 10 (0.0%) |
| Small fibre neuropathy | - | - | 1 (0.0%) | - | - | - | 1 (0.0%) |
| Snoring | - | - | 2 (0.1%) | - | - | 4 (0.0%) | 6 (0.0%) |
| Sopor | - | - | 2 (0.1%) | - | - | 15 (0.1%) | 17 (0.0%) |
| Spinal cord compression | - | - | 1 (0.0%) | - | - | 9 (0.0%) | 10 (0.0%) |
| Spinal cord disorder | - | - | 1 (0.0%) | - | - | 1 (0.0%) | 2 (0.0%) |
| Spinal cord infarction | - | - | 1 (0.0%) | - | - | - | 1 (0.0%) |
| Spinal epidural haematoma | - | - | 1 (0.0%) | - | - | - | 1 (0.0%) |
| Spinal pain | - | - | 1 (0.0%) | - | - | 3 (0.0%) | 4 (0.0%) |
| Splenic rupture | - | - | 1 (0.0%) | - | - | 1 (0.0%) | 2 (0.0%) |
| Sputum discoloured | - | - | 1 (0.0%) | - | - | - | 1 (0.0%) |
| Sputum retention | - | - | 1 (0.0%) | - | - | 1 (0.0%) | 2 (0.0%) |
| Status epilepticus | - | - | 5 (0.1%) | - | - | 7 (0.0%) | 12 (0.0%) |
| Stereotypy | - | - | 3 (0.1%) | - | - | 1 (0.0%) | 4 (0.0%) |
| Strabismus | - | - | 2 (0.1%) | - | - | 1 (0.0%) | 3 (0.0%) |
| Stress cardiomyopathy | - | - | 2 (0.1%) | - | - | 5 (0.0%) | 7 (0.0%) |
| Subacute combined cord degeneration | - | - | 1 (0.0%) | - | - | - | 1 (0.0%) |
| Subarachnoid haemorrhage | - | - | 2 (0.1%) | - | - | 2 (0.0%) | 4 (0.0%) |
| Substance dependence | - | - | 4 (0.1%) | 1 (0.3%) | - | - | 5 (0.0%) |
| Substance use disorder | - | - | 1 (0.0%) | - | - | 1 (0.0%) | 2 (0.0%) |
| Sudden onset of sleep | - | - | 2 (0.1%) | - | - | 2 (0.0%) | 4 (0.0%) |
| Supraventricular extrasystoles | - | - | 1 (0.0%) | - | - | 1 (0.0%) | 2 (0.0%) |
| Supraventricular tachycardia | - | - | 2 (0.1%) | - | 1 (0.2%) | 2 (0.0%) | 5 (0.0%) |
| Suspected product contamination | - | - | 1 (0.0%) | - | - | - | 1 (0.0%) |
| Syringomyelia | - | - | 1 (0.0%) | - | - | - | 1 (0.0%) |
| Tachyarrhythmia | - | - | 1 (0.0%) | - | - | 3 (0.0%) | 4 (0.0%) |
| Tachypnoea | - | - | 8 (0.2%) | - | - | 6 (0.0%) | 14 (0.0%) |
| Tardive dyskinesia | - | - | 2 (0.1%) | - | - | 3 (0.0%) | 5 (0.0%) |
| Temperature intolerance | - | - | 1 (0.0%) | - | - | 2 (0.0%) | 3 (0.0%) |
| Therapeutic product effect incomplete | - | - | 4 (0.1%) | 1 (0.3%) | - | 17 (0.1%) | 22 (0.1%) |
| Therapeutic product effect prolonged | - | - | 1 (0.0%) | - | - | 1 (0.0%) | 2 (0.0%) |
| Therapeutic product ineffective for unapproved indication | - | - | 1 (0.0%) | - | - | - | 1 (0.0%) |
| Therapeutic response decreased | - | - | 2 (0.1%) | - | - | 115 (0.4%) | 117 (0.3%) |
| Therapy non-responder | - | - | 1 (0.0%) | - | - | 2 (0.0%) | 3 (0.0%) |
| Thirst | - | - | 2 (0.1%) | - | - | 8 (0.0%) | 10 (0.0%) |
| Thrombophlebitis | - | - | 1 (0.0%) | - | - | - | 1 (0.0%) |
| Tinnitus | - | - | 3 (0.1%) | - | - | 15 (0.1%) | 18 (0.1%) |
| Tissue anoxia | - | - | 1 (0.0%) | - | - | - | 1 (0.0%) |
| Tongue movement disturbance | - | - | 1 (0.0%) | - | - | - | 1 (0.0%) |
| Tonic clonic movements | - | - | 4 (0.1%) | - | - | 2 (0.0%) | 6 (0.0%) |
| Tonic convulsion | - | - | 2 (0.1%) | - | - | 5 (0.0%) | 7 (0.0%) |
| Torsade de pointes | - | - | 1 (0.0%) | - | - | 2 (0.0%) | 3 (0.0%) |
| Toxic encephalopathy | - | - | 2 (0.1%) | - | 1 (0.2%) | 11 (0.0%) | 14 (0.0%) |
| Toxic leukoencephalopathy | - | - | 2 (0.1%) | - | - | 1 (0.0%) | 3 (0.0%) |
| Tracheal oedema | - | - | 1 (0.0%) | - | - | - | 1 (0.0%) |
| Transaminases increased | - | - | 1 (0.0%) | - | - | 8 (0.0%) | 9 (0.0%) |
| Transfusion | - | - | 1 (0.0%) | - | - | - | 1 (0.0%) |
| Transplant failure | - | - | 1 (0.0%) | - | - | - | 1 (0.0%) |
| Treatment noncompliance | - | - | 3 (0.1%) | - | - | 33 (0.1%) | 36 (0.1%) |
| Tricuspid valve incompetence | - | - | 1 (0.0%) | - | - | 2 (0.0%) | 3 (0.0%) |
| Trigeminal nerve disorder | - | - | 1 (0.0%) | - | - | - | 1 (0.0%) |
| Trigemino-cardiac reflex | - | - | 1 (0.0%) | - | - | - | 1 (0.0%) |
| Trismus | - | - | 4 (0.1%) | - | - | 3 (0.0%) | 7 (0.0%) |
| Troponin I increased | - | - | 1 (0.0%) | - | - | - | 1 (0.0%) |
| Troponin increased | - | - | 4 (0.1%) | - | - | 4 (0.0%) | 8 (0.0%) |
| Upper airway obstruction | - | - | 4 (0.1%) | - | - | - | 4 (0.0%) |
| Upper respiratory tract infection bacterial | - | - | 1 (0.0%) | - | - | - | 1 (0.0%) |
| Uraemic encephalopathy | - | - | 1 (0.0%) | - | - | - | 1 (0.0%) |
| Urinary incontinence | - | - | 1 (0.0%) | - | 1 (0.2%) | 27 (0.1%) | 29 (0.1%) |
| Urinary tract infection | - | - | 1 (0.0%) | - | 1 (0.2%) | 39 (0.1%) | 41 (0.1%) |
| Urinary tract obstruction | - | - | 1 (0.0%) | - | - | - | 1 (0.0%) |
| Urticaria | - | - | 5 (0.1%) | - | - | 12 (0.0%) | 17 (0.0%) |
| Uterine haemorrhage | - | - | 2 (0.1%) | - | - | 1 (0.0%) | 3 (0.0%) |
| Uterine hypotonus | - | - | 1 (0.0%) | - | - | - | 1 (0.0%) |
| Vaginal discharge | - | - | 7 (0.2%) | - | - | 1 (0.0%) | 8 (0.0%) |
| Vaginal flatulence | - | - | 7 (0.2%) | - | - | - | 7 (0.0%) |
| Vaginal haemorrhage | - | - | 1 (0.0%) | - | - | 3 (0.0%) | 4 (0.0%) |
| Vasospasm | - | - | 1 (0.0%) | - | - | - | 1 (0.0%) |
| Venoocclusive disease | - | - | 1 (0.0%) | - | - | - | 1 (0.0%) |
| Ventricular dysfunction | - | - | 1 (0.0%) | - | - | - | 1 (0.0%) |
| Ventricular extrasystoles | - | - | 4 (0.1%) | - | - | 2 (0.0%) | 6 (0.0%) |
| Ventricular fibrillation | - | - | 5 (0.1%) | - | - | - | 5 (0.0%) |
| VIIth nerve injury | - | - | 1 (0.0%) | - | - | - | 1 (0.0%) |
| Visual acuity reduced | - | - | 1 (0.0%) | - | - | 2 (0.0%) | 3 (0.0%) |
| Visual field defect | - | - | 1 (0.0%) | 1 (0.3%) | - | 3 (0.0%) | 5 (0.0%) |
| Visual impairment | - | - | 4 (0.1%) | - | - | 44 (0.2%) | 48 (0.1%) |
| Vocal cord paralysis | - | - | 2 (0.1%) | - | - | - | 2 (0.0%) |
| Vocal cord paresis | - | - | 1 (0.0%) | - | - | - | 1 (0.0%) |
| Volume blood decreased | - | - | 1 (0.0%) | - | - | - | 1 (0.0%) |
| Vth nerve injury | - | - | 1 (0.0%) | - | - | - | 1 (0.0%) |
| White blood cell count increased | - | - | 3 (0.1%) | - | - | 11 (0.0%) | 14 (0.0%) |
| Wrong dose | - | - | 1 (0.0%) | - | - | 3 (0.0%) | 4 (0.0%) |
| Wrong patient received product | - | - | 1 (0.0%) | - | - | 2 (0.0%) | 3 (0.0%) |
| Wrong product administered | - | - | 6 (0.2%) | 2 (0.5%) | - | 3 (0.0%) | 11 (0.0%) |
| Wrong route | - | - | 1 (0.0%) | - | - | - | 1 (0.0%) |
| Aspiration | - | - | - | 2 (0.5%) | 1 (0.2%) | 22 (0.1%) | 25 (0.1%) |
| Breast cancer metastatic | - | - | - | 1 (0.3%) | - | 4 (0.0%) | 5 (0.0%) |
| Cardiovascular disorder | - | - | - | 1 (0.3%) | - | 10 (0.0%) | 11 (0.0%) |
| Carotid artery stenosis | - | - | - | 1 (0.3%) | - | 1 (0.0%) | 2 (0.0%) |
| Cervical radiculopathy | - | - | - | 1 (0.3%) | - | 1 (0.0%) | 2 (0.0%) |
| Chemical burn | - | - | - | 1 (0.3%) | - | 1 (0.0%) | 2 (0.0%) |
| Cheyne-Stokes respiration | - | - | - | 1 (0.3%) | - | 4 (0.0%) | 5 (0.0%) |
| Child neglect | - | - | - | 2 (0.5%) | - | - | 2 (0.0%) |
| Cluster headache | - | - | - | 1 (0.3%) | - | 3 (0.0%) | 4 (0.0%) |
| Congestive hepatopathy | - | - | - | 1 (0.3%) | - | 1 (0.0%) | 2 (0.0%) |
| Counterfeit product administered | - | - | - | 1 (0.3%) | - | - | 1 (0.0%) |
| Deafness neurosensory | - | - | - | 1 (0.3%) | - | - | 1 (0.0%) |
| Drop attacks | - | - | - | 1 (0.3%) | - | 4 (0.0%) | 5 (0.0%) |
| Erysipelas | - | - | - | 1 (0.3%) | - | - | 1 (0.0%) |
| Feeling of relaxation | - | - | - | 1 (0.3%) | - | - | 1 (0.0%) |
| Hepatomegaly | - | - | - | 1 (0.3%) | - | 2 (0.0%) | 3 (0.0%) |
| Ischaemic stroke | - | - | - | 1 (0.3%) | - | 2 (0.0%) | 3 (0.0%) |
| Joint stiffness | - | - | - | 1 (0.3%) | - | 3 (0.0%) | 4 (0.0%) |
| Major depression | - | - | - | 1 (0.3%) | - | 5 (0.0%) | 6 (0.0%) |
| Meningococcal bacteraemia | - | - | - | 1 (0.3%) | - | - | 1 (0.0%) |
| Mitochondrial toxicity | - | - | - | 2 (0.5%) | 1 (0.2%) | 1 (0.0%) | 4 (0.0%) |
| Mood altered | - | - | - | 1 (0.3%) | - | 13 (0.0%) | 14 (0.0%) |
| Needle track marks | - | - | - | 1 (0.3%) | - | - | 1 (0.0%) |
| Neonatal respiratory failure | - | - | - | 1 (0.3%) | - | - | 1 (0.0%) |
| Pneumonitis chemical | - | - | - | 2 (0.5%) | - | - | 2 (0.0%) |
| Procedural hypotension | - | - | - | 1 (0.3%) | - | - | 1 (0.0%) |
| Product appearance confusion | - | - | - | 1 (0.3%) | - | - | 1 (0.0%) |
| Product container issue | - | - | - | 1 (0.3%) | - | - | 1 (0.0%) |
| Pulmonary alveolar haemorrhage | - | - | - | 3 (0.8%) | - | 1 (0.0%) | 4 (0.0%) |
| Retinopathy | - | - | - | 1 (0.3%) | - | - | 1 (0.0%) |
| Skin mass | - | - | - | 1 (0.3%) | - | 1 (0.0%) | 2 (0.0%) |
| Therapy interrupted | - | - | - | 1 (0.3%) | - | - | 1 (0.0%) |
| Vein collapse | - | - | - | 1 (0.3%) | - | - | 1 (0.0%) |
| Application site erythema | - | - | - | - | 1 (0.2%) | 42 (0.1%) | 43 (0.1%) |
| Autoimmune hepatitis | - | - | - | - | 1 (0.2%) | - | 1 (0.0%) |
| Biopsy liver | - | - | - | - | 1 (0.2%) | - | 1 (0.0%) |
| Blood albumin decreased | - | - | - | - | 1 (0.2%) | 4 (0.0%) | 5 (0.0%) |
| Blood alkaline phosphatase increased | - | - | - | - | 1 (0.2%) | 11 (0.0%) | 12 (0.0%) |
| Blood calcium decreased | - | - | - | - | 1 (0.2%) | 3 (0.0%) | 4 (0.0%) |
| Blood prolactin increased | - | - | - | - | 1 (0.2%) | - | 1 (0.0%) |
| Cancer fatigue | - | - | - | - | 1 (0.2%) | - | 1 (0.0%) |
| Cholecystitis acute | - | - | - | - | 1 (0.2%) | - | 1 (0.0%) |
| Clostridium difficile colitis | - | - | - | - | 1 (0.2%) | 1 (0.0%) | 2 (0.0%) |
| Colorectal cancer metastatic | - | - | - | - | 1 (0.2%) | 1 (0.0%) | 2 (0.0%) |
| Dementia Alzheimer's type | - | - | - | - | 1 (0.2%) | 6 (0.0%) | 7 (0.0%) |
| Device adhesion issue | - | - | - | - | 1 (0.2%) | 9 (0.0%) | 10 (0.0%) |
| Diabetic complication | - | - | - | - | 1 (0.2%) | - | 1 (0.0%) |
| Dialysis | - | - | - | - | 1 (0.2%) | 1 (0.0%) | 2 (0.0%) |
| Drug level decreased | - | - | - | - | 1 (0.2%) | 3 (0.0%) | 4 (0.0%) |
| Encephalomalacia | - | - | - | - | 1 (0.2%) | 1 (0.0%) | 2 (0.0%) |
| Faecal vomiting | - | - | - | - | 1 (0.2%) | 1 (0.0%) | 2 (0.0%) |
| Failed back surgery syndrome | - | - | - | - | 1 (0.2%) | 1 (0.0%) | 2 (0.0%) |
| Haematocrit decreased | - | - | - | - | 1 (0.2%) | 2 (0.0%) | 3 (0.0%) |
| Head injury | - | - | - | - | 2 (0.4%) | 21 (0.1%) | 23 (0.1%) |
| Hepatic enzyme increased | - | - | - | - | 1 (0.2%) | 7 (0.0%) | 8 (0.0%) |
| Intestinal perforation | - | - | - | - | 1 (0.2%) | 2 (0.0%) | 3 (0.0%) |
| Left ventricular hypertrophy | - | - | - | - | 1 (0.2%) | 3 (0.0%) | 4 (0.0%) |
| Lung neoplasm malignant | - | - | - | - | 1 (0.2%) | 15 (0.1%) | 16 (0.0%) |
| Lupus-like syndrome | - | - | - | - | 1 (0.2%) | - | 1 (0.0%) |
| Metastases to spine | - | - | - | - | 1 (0.2%) | 2 (0.0%) | 3 (0.0%) |
| Nephropathy | - | - | - | - | 1 (0.2%) | - | 1 (0.0%) |
| Oesophageal achalasia | - | - | - | - | 1 (0.2%) | - | 1 (0.0%) |
| Osteoporosis | - | - | - | - | 1 (0.2%) | 17 (0.1%) | 18 (0.1%) |
| Pain threshold decreased | - | - | - | - | 1 (0.2%) | - | 1 (0.0%) |
| Partner stress | - | - | - | - | 2 (0.4%) | 1 (0.0%) | 3 (0.0%) |
| Pleurisy | - | - | - | - | 1 (0.2%) | - | 1 (0.0%) |
| Pneumonia bacterial | - | - | - | - | 1 (0.2%) | 1 (0.0%) | 2 (0.0%) |
| Pneumonitis | - | - | - | - | 1 (0.2%) | 1 (0.0%) | 2 (0.0%) |
| Product adhesion issue | - | - | - | - | 1 (0.2%) | 156 (0.5%) | 157 (0.4%) |
| Sensitisation | - | - | - | - | 1 (0.2%) | - | 1 (0.0%) |
| Surgery | - | - | - | - | 1 (0.2%) | 19 (0.1%) | 20 (0.1%) |
| Systemic inflammatory response syndrome | - | - | - | - | 1 (0.2%) | - | 1 (0.0%) |
| Thoracic cavity drainage | - | - | - | - | 1 (0.2%) | - | 1 (0.0%) |
| Tumour pain | - | - | - | - | 1 (0.2%) | - | 1 (0.0%) |
| Urine output decreased | - | - | - | - | 1 (0.2%) | 6 (0.0%) | 7 (0.0%) |
| Venous occlusion | - | - | - | - | 1 (0.2%) | - | 1 (0.0%) |
| Venous thrombosis | - | - | - | - | 1 (0.2%) | - | 1 (0.0%) |
| Victim of homicide | - | - | - | - | 1 (0.2%) | 1 (0.0%) | 2 (0.0%) |
| Abdominal discomfort | - | - | - | - | - | 37 (0.1%) | 37 (0.1%) |
| Abdominal hernia | - | - | - | - | - | 2 (0.0%) | 2 (0.0%) |
| Abdominal hernia obstructive | - | - | - | - | - | 1 (0.0%) | 1 (0.0%) |
| Abdominal wall abscess | - | - | - | - | - | 2 (0.0%) | 2 (0.0%) |
| Abnormal dreams | - | - | - | - | - | 14 (0.0%) | 14 (0.0%) |
| Abnormal loss of weight | - | - | - | - | - | 31 (0.1%) | 31 (0.1%) |
| Abnormal sleep-related event | - | - | - | - | - | 1 (0.0%) | 1 (0.0%) |
| Abnormal weight gain | - | - | - | - | - | 1 (0.0%) | 1 (0.0%) |
| Abulia | - | - | - | - | - | 3 (0.0%) | 3 (0.0%) |
| Accident | - | - | - | - | - | 1 (0.0%) | 1 (0.0%) |
| Accidental death | - | - | - | - | - | 1 (0.0%) | 1 (0.0%) |
| Accidental poisoning | - | - | - | - | - | 3 (0.0%) | 3 (0.0%) |
| Acetabulum fracture | - | - | - | - | - | 1 (0.0%) | 1 (0.0%) |
| Acne | - | - | - | - | - | 1 (0.0%) | 1 (0.0%) |
| Acoustic neuroma | - | - | - | - | - | 1 (0.0%) | 1 (0.0%) |
| Activated partial thromboplastin time prolonged | - | - | - | - | - | 1 (0.0%) | 1 (0.0%) |
| Acute coronary syndrome | - | - | - | - | - | 2 (0.0%) | 2 (0.0%) |
| Acute hepatic failure | - | - | - | - | - | 1 (0.0%) | 1 (0.0%) |
| Acute psychosis | - | - | - | - | - | 1 (0.0%) | 1 (0.0%) |
| Adhesion | - | - | - | - | - | 1 (0.0%) | 1 (0.0%) |
| Adjustment disorder with depressed mood | - | - | - | - | - | 1 (0.0%) | 1 (0.0%) |
| Adrenal disorder | - | - | - | - | - | 1 (0.0%) | 1 (0.0%) |
| Adrenal insufficiency | - | - | - | - | - | 3 (0.0%) | 3 (0.0%) |
| Adverse reaction | - | - | - | - | - | 6 (0.0%) | 6 (0.0%) |
| Affective disorder | - | - | - | - | - | 3 (0.0%) | 3 (0.0%) |
| Agoraphobia | - | - | - | - | - | 1 (0.0%) | 1 (0.0%) |
| Alanine aminotransferase | - | - | - | - | - | 1 (0.0%) | 1 (0.0%) |
| Alcohol interaction | - | - | - | - | - | 2 (0.0%) | 2 (0.0%) |
| Alcohol poisoning | - | - | - | - | - | 1 (0.0%) | 1 (0.0%) |
| Alcohol problem | - | - | - | - | - | 1 (0.0%) | 1 (0.0%) |
| Alcoholisation procedure | - | - | - | - | - | 1 (0.0%) | 1 (0.0%) |
| Alopecia | - | - | - | - | - | 16 (0.1%) | 16 (0.0%) |
| Amenorrhoea | - | - | - | - | - | 1 (0.0%) | 1 (0.0%) |
| Amyotrophic lateral sclerosis | - | - | - | - | - | 4 (0.0%) | 4 (0.0%) |
| Anal injury | - | - | - | - | - | 1 (0.0%) | 1 (0.0%) |
| Anal sphincter atony | - | - | - | - | - | 1 (0.0%) | 1 (0.0%) |
| Anal stenosis | - | - | - | - | - | 1 (0.0%) | 1 (0.0%) |
| Analgesic drug level decreased | - | - | - | - | - | 1 (0.0%) | 1 (0.0%) |
| Analgesic drug level increased | - | - | - | - | - | 4 (0.0%) | 4 (0.0%) |
| Analgesic therapy | - | - | - | - | - | 1 (0.0%) | 1 (0.0%) |
| Anaphylactoid reaction | - | - | - | - | - | 3 (0.0%) | 3 (0.0%) |
| Aneurysm | - | - | - | - | - | 2 (0.0%) | 2 (0.0%) |
| Angina pectoris | - | - | - | - | - | 7 (0.0%) | 7 (0.0%) |
| Angina unstable | - | - | - | - | - | 1 (0.0%) | 1 (0.0%) |
| Anhedonia | - | - | - | - | - | 1 (0.0%) | 1 (0.0%) |
| Anion gap abnormal | - | - | - | - | - | 1 (0.0%) | 1 (0.0%) |
| Ankle fracture | - | - | - | - | - | 7 (0.0%) | 7 (0.0%) |
| Ankylosing spondylitis | - | - | - | - | - | 1 (0.0%) | 1 (0.0%) |
| Anorectal discomfort | - | - | - | - | - | 1 (0.0%) | 1 (0.0%) |
| Anoxia | - | - | - | - | - | 1 (0.0%) | 1 (0.0%) |
| Anti-erythropoietin antibody | - | - | - | - | - | 1 (0.0%) | 1 (0.0%) |
| Anticonvulsant drug level above therapeutic | - | - | - | - | - | 1 (0.0%) | 1 (0.0%) |
| Antinuclear antibody increased | - | - | - | - | - | 1 (0.0%) | 1 (0.0%) |
| Antisocial behaviour | - | - | - | - | - | 1 (0.0%) | 1 (0.0%) |
| Aortic aneurysm | - | - | - | - | - | 1 (0.0%) | 1 (0.0%) |
| Aortic arteriosclerosis | - | - | - | - | - | 2 (0.0%) | 2 (0.0%) |
| Aortic dilatation | - | - | - | - | - | 1 (0.0%) | 1 (0.0%) |
| Aortic thrombosis | - | - | - | - | - | 1 (0.0%) | 1 (0.0%) |
| Aortic valve disease | - | - | - | - | - | 1 (0.0%) | 1 (0.0%) |
| Aortic valve prolapse | - | - | - | - | - | 1 (0.0%) | 1 (0.0%) |
| Aortic valve stenosis | - | - | - | - | - | 1 (0.0%) | 1 (0.0%) |
| Aphonia | - | - | - | - | - | 3 (0.0%) | 3 (0.0%) |
| Aplastic anaemia | - | - | - | - | - | 1 (0.0%) | 1 (0.0%) |
| Apnoeic attack | - | - | - | - | - | 1 (0.0%) | 1 (0.0%) |
| Appendicectomy | - | - | - | - | - | 1 (0.0%) | 1 (0.0%) |
| Application site bruise | - | - | - | - | - | 1 (0.0%) | 1 (0.0%) |
| Application site burn | - | - | - | - | - | 9 (0.0%) | 9 (0.0%) |
| Application site dermatitis | - | - | - | - | - | 2 (0.0%) | 2 (0.0%) |
| Application site discharge | - | - | - | - | - | 1 (0.0%) | 1 (0.0%) |
| Application site discolouration | - | - | - | - | - | 6 (0.0%) | 6 (0.0%) |
| Application site discomfort | - | - | - | - | - | 1 (0.0%) | 1 (0.0%) |
| Application site dryness | - | - | - | - | - | 2 (0.0%) | 2 (0.0%) |
| Application site eczema | - | - | - | - | - | 1 (0.0%) | 1 (0.0%) |
| Application site erosion | - | - | - | - | - | 12 (0.0%) | 12 (0.0%) |
| Application site exfoliation | - | - | - | - | - | 2 (0.0%) | 2 (0.0%) |
| Application site haemorrhage | - | - | - | - | - | 2 (0.0%) | 2 (0.0%) |
| Application site hypersensitivity | - | - | - | - | - | 1 (0.0%) | 1 (0.0%) |
| Application site infection | - | - | - | - | - | 3 (0.0%) | 3 (0.0%) |
| Application site inflammation | - | - | - | - | - | 2 (0.0%) | 2 (0.0%) |
| Application site pain | - | - | - | - | - | 22 (0.1%) | 22 (0.1%) |
| Application site perspiration | - | - | - | - | - | 2 (0.0%) | 2 (0.0%) |
| Application site pruritus | - | - | - | - | - | 43 (0.1%) | 43 (0.1%) |
| Application site pustules | - | - | - | - | - | 4 (0.0%) | 4 (0.0%) |
| Application site rash | - | - | - | - | - | 48 (0.2%) | 48 (0.1%) |
| Application site reaction | - | - | - | - | - | 57 (0.2%) | 57 (0.2%) |
| Application site scab | - | - | - | - | - | 3 (0.0%) | 3 (0.0%) |
| Application site scar | - | - | - | - | - | 6 (0.0%) | 6 (0.0%) |
| Application site swelling | - | - | - | - | - | 4 (0.0%) | 4 (0.0%) |
| Application site ulcer | - | - | - | - | - | 5 (0.0%) | 5 (0.0%) |
| Application site urticaria | - | - | - | - | - | 8 (0.0%) | 8 (0.0%) |
| Application site warmth | - | - | - | - | - | 2 (0.0%) | 2 (0.0%) |
| Apraxia | - | - | - | - | - | 4 (0.0%) | 4 (0.0%) |
| Aptyalism | - | - | - | - | - | 1 (0.0%) | 1 (0.0%) |
| Arnold-Chiari malformation | - | - | - | - | - | 1 (0.0%) | 1 (0.0%) |
| Arrhythmia supraventricular | - | - | - | - | - | 1 (0.0%) | 1 (0.0%) |
| Arterial disorder | - | - | - | - | - | 1 (0.0%) | 1 (0.0%) |
| Arterial haemorrhage | - | - | - | - | - | 1 (0.0%) | 1 (0.0%) |
| Arterial occlusive disease | - | - | - | - | - | 4 (0.0%) | 4 (0.0%) |
| Arteriosclerosis coronary artery | - | - | - | - | - | 2 (0.0%) | 2 (0.0%) |
| Arthritis bacterial | - | - | - | - | - | 1 (0.0%) | 1 (0.0%) |
| Arthropod bite | - | - | - | - | - | 1 (0.0%) | 1 (0.0%) |
| Arthropod sting | - | - | - | - | - | 1 (0.0%) | 1 (0.0%) |
| Asthmatic crisis | - | - | - | - | - | 1 (0.0%) | 1 (0.0%) |
| Astigmatism | - | - | - | - | - | 1 (0.0%) | 1 (0.0%) |
| Astringent therapy | - | - | - | - | - | 1 (0.0%) | 1 (0.0%) |
| Atonic seizures | - | - | - | - | - | 1 (0.0%) | 1 (0.0%) |
| Atrial tachycardia | - | - | - | - | - | 1 (0.0%) | 1 (0.0%) |
| Atrioventricular block first degree | - | - | - | - | - | 1 (0.0%) | 1 (0.0%) |
| Atrophy | - | - | - | - | - | 1 (0.0%) | 1 (0.0%) |
| Attention deficit hyperactivity disorder | - | - | - | - | - | 1 (0.0%) | 1 (0.0%) |
| Auditory disorder | - | - | - | - | - | 1 (0.0%) | 1 (0.0%) |
| Autoimmune colitis | - | - | - | - | - | 1 (0.0%) | 1 (0.0%) |
| Autoimmune disorder | - | - | - | - | - | 2 (0.0%) | 2 (0.0%) |
| Autoimmune thyroiditis | - | - | - | - | - | 1 (0.0%) | 1 (0.0%) |
| Autonomic neuropathy | - | - | - | - | - | 2 (0.0%) | 2 (0.0%) |
| Azotaemia | - | - | - | - | - | 1 (0.0%) | 1 (0.0%) |
| Bacillus infection | - | - | - | - | - | 1 (0.0%) | 1 (0.0%) |
| Back injury | - | - | - | - | - | 7 (0.0%) | 7 (0.0%) |
| Bacterial disease carrier | - | - | - | - | - | 1 (0.0%) | 1 (0.0%) |
| Bacterial food poisoning | - | - | - | - | - | 1 (0.0%) | 1 (0.0%) |
| Bacterial infection | - | - | - | - | - | 2 (0.0%) | 2 (0.0%) |
| Bacterial test | - | - | - | - | - | 1 (0.0%) | 1 (0.0%) |
| Bacterial vaginosis | - | - | - | - | - | 1 (0.0%) | 1 (0.0%) |
| Barrett's oesophagus | - | - | - | - | - | 1 (0.0%) | 1 (0.0%) |
| Basal ganglia infarction | - | - | - | - | - | 1 (0.0%) | 1 (0.0%) |
| Bell's palsy | - | - | - | - | - | 1 (0.0%) | 1 (0.0%) |
| Benign breast neoplasm | - | - | - | - | - | 2 (0.0%) | 2 (0.0%) |
| Benign neoplasm | - | - | - | - | - | 1 (0.0%) | 1 (0.0%) |
| Benign prostatic hyperplasia | - | - | - | - | - | 1 (0.0%) | 1 (0.0%) |
| Bile duct cancer | - | - | - | - | - | 1 (0.0%) | 1 (0.0%) |
| Bile duct stone | - | - | - | - | - | 2 (0.0%) | 2 (0.0%) |
| Biliary colic | - | - | - | - | - | 1 (0.0%) | 1 (0.0%) |
| Biliary dilatation | - | - | - | - | - | 3 (0.0%) | 3 (0.0%) |
| Biliary tract disorder | - | - | - | - | - | 1 (0.0%) | 1 (0.0%) |
| Biliary tract infection | - | - | - | - | - | 1 (0.0%) | 1 (0.0%) |
| Bipolar disorder | - | - | - | - | - | 12 (0.0%) | 12 (0.0%) |
| Bipolar I disorder | - | - | - | - | - | 2 (0.0%) | 2 (0.0%) |
| Bladder cancer | - | - | - | - | - | 1 (0.0%) | 1 (0.0%) |
| Bladder dilatation | - | - | - | - | - | 3 (0.0%) | 3 (0.0%) |
| Bladder outlet obstruction | - | - | - | - | - | 1 (0.0%) | 1 (0.0%) |
| Bladder stenosis | - | - | - | - | - | 1 (0.0%) | 1 (0.0%) |
| Bleeding varicose vein | - | - | - | - | - | 1 (0.0%) | 1 (0.0%) |
| Blindness unilateral | - | - | - | - | - | 4 (0.0%) | 4 (0.0%) |
| Blister | - | - | - | - | - | 13 (0.0%) | 13 (0.0%) |
| Blood 1,25-dihydroxycholecalciferol increased | - | - | - | - | - | 1 (0.0%) | 1 (0.0%) |
| Blood albumin abnormal | - | - | - | - | - | 1 (0.0%) | 1 (0.0%) |
| Blood calcium increased | - | - | - | - | - | 3 (0.0%) | 3 (0.0%) |
| Blood cholesterol increased | - | - | - | - | - | 22 (0.1%) | 22 (0.1%) |
| Blood disorder | - | - | - | - | - | 1 (0.0%) | 1 (0.0%) |
| Blood glucose abnormal | - | - | - | - | - | 3 (0.0%) | 3 (0.0%) |
| Blood glucose decreased | - | - | - | - | - | 12 (0.0%) | 12 (0.0%) |
| Blood glucose fluctuation | - | - | - | - | - | 1 (0.0%) | 1 (0.0%) |
| Blood glucose increased | - | - | - | - | - | 19 (0.1%) | 19 (0.1%) |
| Blood iron decreased | - | - | - | - | - | 1 (0.0%) | 1 (0.0%) |
| Blood magnesium abnormal | - | - | - | - | - | 1 (0.0%) | 1 (0.0%) |
| Blood magnesium decreased | - | - | - | - | - | 8 (0.0%) | 8 (0.0%) |
| Blood magnesium increased | - | - | - | - | - | 1 (0.0%) | 1 (0.0%) |
| Blood oestrogen decreased | - | - | - | - | - | 1 (0.0%) | 1 (0.0%) |
| Blood potassium abnormal | - | - | - | - | - | 2 (0.0%) | 2 (0.0%) |
| Blood pressure diastolic decreased | - | - | - | - | - | 2 (0.0%) | 2 (0.0%) |
| Blood pressure orthostatic | - | - | - | - | - | 1 (0.0%) | 1 (0.0%) |
| Blood sodium decreased | - | - | - | - | - | 5 (0.0%) | 5 (0.0%) |
| Blood test abnormal | - | - | - | - | - | 1 (0.0%) | 1 (0.0%) |
| Blood testosterone decreased | - | - | - | - | - | 3 (0.0%) | 3 (0.0%) |
| Blood triglycerides abnormal | - | - | - | - | - | 1 (0.0%) | 1 (0.0%) |
| Blood triglycerides increased | - | - | - | - | - | 1 (0.0%) | 1 (0.0%) |
| Body dysmorphic disorder | - | - | - | - | - | 4 (0.0%) | 4 (0.0%) |
| Body height decreased | - | - | - | - | - | 23 (0.1%) | 23 (0.1%) |
| Body temperature | - | - | - | - | - | 1 (0.0%) | 1 (0.0%) |
| Body temperature decreased | - | - | - | - | - | 3 (0.0%) | 3 (0.0%) |
| Body temperature fluctuation | - | - | - | - | - | 2 (0.0%) | 2 (0.0%) |
| Bone cancer | - | - | - | - | - | 2 (0.0%) | 2 (0.0%) |
| Bone density decreased | - | - | - | - | - | 1 (0.0%) | 1 (0.0%) |
| Bone lesion | - | - | - | - | - | 1 (0.0%) | 1 (0.0%) |
| Bowel movement irregularity | - | - | - | - | - | 1 (0.0%) | 1 (0.0%) |
| Brachial plexus injury | - | - | - | - | - | 1 (0.0%) | 1 (0.0%) |
| Brain cancer metastatic | - | - | - | - | - | 2 (0.0%) | 2 (0.0%) |
| Brain neoplasm malignant | - | - | - | - | - | 2 (0.0%) | 2 (0.0%) |
| Brain operation | - | - | - | - | - | 1 (0.0%) | 1 (0.0%) |
| Brain scan abnormal | - | - | - | - | - | 2 (0.0%) | 2 (0.0%) |
| Brain stem infarction | - | - | - | - | - | 1 (0.0%) | 1 (0.0%) |
| Brain stem ischaemia | - | - | - | - | - | 1 (0.0%) | 1 (0.0%) |
| Brain stem syndrome | - | - | - | - | - | 1 (0.0%) | 1 (0.0%) |
| Breast cancer recurrent | - | - | - | - | - | 1 (0.0%) | 1 (0.0%) |
| Breast discharge | - | - | - | - | - | 1 (0.0%) | 1 (0.0%) |
| Breast enlargement | - | - | - | - | - | 1 (0.0%) | 1 (0.0%) |
| Breast operation | - | - | - | - | - | 1 (0.0%) | 1 (0.0%) |
| Breast tenderness | - | - | - | - | - | 1 (0.0%) | 1 (0.0%) |
| Breath odour | - | - | - | - | - | 1 (0.0%) | 1 (0.0%) |
| Breath sounds abnormal | - | - | - | - | - | 8 (0.0%) | 8 (0.0%) |
| Breath sounds absent | - | - | - | - | - | 1 (0.0%) | 1 (0.0%) |
| Breathing-related sleep disorder | - | - | - | - | - | 1 (0.0%) | 1 (0.0%) |
| Bronchial aspiration procedure | - | - | - | - | - | 3 (0.0%) | 3 (0.0%) |
| Bronchial obstruction | - | - | - | - | - | 1 (0.0%) | 1 (0.0%) |
| Bronchitis chronic | - | - | - | - | - | 1 (0.0%) | 1 (0.0%) |
| Bruxism | - | - | - | - | - | 3 (0.0%) | 3 (0.0%) |
| Burn infection | - | - | - | - | - | 1 (0.0%) | 1 (0.0%) |
| Burns second degree | - | - | - | - | - | 4 (0.0%) | 4 (0.0%) |
| Bursa disorder | - | - | - | - | - | 1 (0.0%) | 1 (0.0%) |
| Bursitis | - | - | - | - | - | 4 (0.0%) | 4 (0.0%) |
| Buttock injury | - | - | - | - | - | 1 (0.0%) | 1 (0.0%) |
| C-reactive protein increased | - | - | - | - | - | 3 (0.0%) | 3 (0.0%) |
| Calcium deficiency | - | - | - | - | - | 1 (0.0%) | 1 (0.0%) |
| Candida infection | - | - | - | - | - | 2 (0.0%) | 2 (0.0%) |
| Cardiac amyloidosis | - | - | - | - | - | 2 (0.0%) | 2 (0.0%) |
| Cardiac aneurysm | - | - | - | - | - | 1 (0.0%) | 1 (0.0%) |
| Cardiac death | - | - | - | - | - | 11 (0.0%) | 11 (0.0%) |
| Cardiac fibrillation | - | - | - | - | - | 1 (0.0%) | 1 (0.0%) |
| Cardiac flutter | - | - | - | - | - | 3 (0.0%) | 3 (0.0%) |
| Cardiac hypertrophy | - | - | - | - | - | 2 (0.0%) | 2 (0.0%) |
| Cardiac murmur | - | - | - | - | - | 1 (0.0%) | 1 (0.0%) |
| Cardiac murmur functional | - | - | - | - | - | 1 (0.0%) | 1 (0.0%) |
| Cardiac pacemaker insertion | - | - | - | - | - | 3 (0.0%) | 3 (0.0%) |
| Cardiac septal defect | - | - | - | - | - | 1 (0.0%) | 1 (0.0%) |
| Cardiac tamponade | - | - | - | - | - | 1 (0.0%) | 1 (0.0%) |
| Cardiac valve disease | - | - | - | - | - | 2 (0.0%) | 2 (0.0%) |
| Cardioactive drug level increased | - | - | - | - | - | 1 (0.0%) | 1 (0.0%) |
| Cardiopulmonary failure | - | - | - | - | - | 4 (0.0%) | 4 (0.0%) |
| Cardioversion | - | - | - | - | - | 1 (0.0%) | 1 (0.0%) |
| Carotid artery dissection | - | - | - | - | - | 1 (0.0%) | 1 (0.0%) |
| Carotid artery occlusion | - | - | - | - | - | 2 (0.0%) | 2 (0.0%) |
| Carpal tunnel syndrome | - | - | - | - | - | 16 (0.1%) | 16 (0.0%) |
| Catabolic state | - | - | - | - | - | 1 (0.0%) | 1 (0.0%) |
| Cataplexy | - | - | - | - | - | 1 (0.0%) | 1 (0.0%) |
| Cataract operation | - | - | - | - | - | 1 (0.0%) | 1 (0.0%) |
| Catatonia | - | - | - | - | - | 2 (0.0%) | 2 (0.0%) |
| Catheter site erosion | - | - | - | - | - | 1 (0.0%) | 1 (0.0%) |
| Catheter site inflammation | - | - | - | - | - | 1 (0.0%) | 1 (0.0%) |
| Catheter site pain | - | - | - | - | - | 1 (0.0%) | 1 (0.0%) |
| Cauda equina syndrome | - | - | - | - | - | 1 (0.0%) | 1 (0.0%) |
| Cellulite | - | - | - | - | - | 1 (0.0%) | 1 (0.0%) |
| Cellulitis | - | - | - | - | - | 13 (0.0%) | 13 (0.0%) |
| Central-alveolar hypoventilation | - | - | - | - | - | 1 (0.0%) | 1 (0.0%) |
| Central sleep apnoea syndrome | - | - | - | - | - | 1 (0.0%) | 1 (0.0%) |
| Cerebellar syndrome | - | - | - | - | - | 1 (0.0%) | 1 (0.0%) |
| Cerebral atrophy | - | - | - | - | - | 7 (0.0%) | 7 (0.0%) |
| Cerebral cyst | - | - | - | - | - | 1 (0.0%) | 1 (0.0%) |
| Cerebral disorder | - | - | - | - | - | 8 (0.0%) | 8 (0.0%) |
| Cerebral hypoperfusion | - | - | - | - | - | 1 (0.0%) | 1 (0.0%) |
| Cerebral ischaemia | - | - | - | - | - | 5 (0.0%) | 5 (0.0%) |
| Cerebral small vessel ischaemic disease | - | - | - | - | - | 1 (0.0%) | 1 (0.0%) |
| Cerebral thrombosis | - | - | - | - | - | 1 (0.0%) | 1 (0.0%) |
| Cervix carcinoma | - | - | - | - | - | 1 (0.0%) | 1 (0.0%) |
| Change of bowel habit | - | - | - | - | - | 1 (0.0%) | 1 (0.0%) |
| Chapped lips | - | - | - | - | - | 1 (0.0%) | 1 (0.0%) |
| Chemical burn of skin | - | - | - | - | - | 2 (0.0%) | 2 (0.0%) |
| Chest X-ray abnormal | - | - | - | - | - | 3 (0.0%) | 3 (0.0%) |
| Choking | - | - | - | - | - | 2 (0.0%) | 2 (0.0%) |
| Choking sensation | - | - | - | - | - | 1 (0.0%) | 1 (0.0%) |
| Cholangitis | - | - | - | - | - | 1 (0.0%) | 1 (0.0%) |
| Cholecystectomy | - | - | - | - | - | 2 (0.0%) | 2 (0.0%) |
| Cholestasis | - | - | - | - | - | 1 (0.0%) | 1 (0.0%) |
| Cholestatic liver injury | - | - | - | - | - | 1 (0.0%) | 1 (0.0%) |
| Cholinergic syndrome | - | - | - | - | - | 2 (0.0%) | 2 (0.0%) |
| Chondropathy | - | - | - | - | - | 2 (0.0%) | 2 (0.0%) |
| Chronic fatigue syndrome | - | - | - | - | - | 1 (0.0%) | 1 (0.0%) |
| Chronic lymphocytic leukaemia | - | - | - | - | - | 2 (0.0%) | 2 (0.0%) |
| Chronic obstructive pulmonary disease | - | - | - | - | - | 17 (0.1%) | 17 (0.0%) |
| Chronic respiratory disease | - | - | - | - | - | 1 (0.0%) | 1 (0.0%) |
| Chronic respiratory failure | - | - | - | - | - | 2 (0.0%) | 2 (0.0%) |
| Clavicle fracture | - | - | - | - | - | 1 (0.0%) | 1 (0.0%) |
| Clostridium difficile infection | - | - | - | - | - | 3 (0.0%) | 3 (0.0%) |
| Clostridium test positive | - | - | - | - | - | 1 (0.0%) | 1 (0.0%) |
| Clubbing | - | - | - | - | - | 1 (0.0%) | 1 (0.0%) |
| Clumsiness | - | - | - | - | - | 4 (0.0%) | 4 (0.0%) |
| Coagulation factor V level decreased | - | - | - | - | - | 1 (0.0%) | 1 (0.0%) |
| Coccydynia | - | - | - | - | - | 1 (0.0%) | 1 (0.0%) |
| Coeliac disease | - | - | - | - | - | 1 (0.0%) | 1 (0.0%) |
| Colostomy | - | - | - | - | - | 1 (0.0%) | 1 (0.0%) |
| Colostomy closure | - | - | - | - | - | 1 (0.0%) | 1 (0.0%) |
| Coma hepatic | - | - | - | - | - | 1 (0.0%) | 1 (0.0%) |
| Compartment syndrome | - | - | - | - | - | 1 (0.0%) | 1 (0.0%) |
| Completed suicide | - | - | - | - | - | 12 (0.0%) | 12 (0.0%) |
| Complex regional pain syndrome | - | - | - | - | - | 12 (0.0%) | 12 (0.0%) |
| Complication associated with device | - | - | - | - | - | 2 (0.0%) | 2 (0.0%) |
| Computerised tomogram abnormal | - | - | - | - | - | 1 (0.0%) | 1 (0.0%) |
| Concomitant disease aggravated | - | - | - | - | - | 5 (0.0%) | 5 (0.0%) |
| Concussion | - | - | - | - | - | 9 (0.0%) | 9 (0.0%) |
| Congenital musculoskeletal disorder of skull | - | - | - | - | - | 1 (0.0%) | 1 (0.0%) |
| Consciousness fluctuating | - | - | - | - | - | 7 (0.0%) | 7 (0.0%) |
| Contrast media reaction | - | - | - | - | - | 1 (0.0%) | 1 (0.0%) |
| Convulsions local | - | - | - | - | - | 2 (0.0%) | 2 (0.0%) |
| Cor pulmonale | - | - | - | - | - | 2 (0.0%) | 2 (0.0%) |
| Corneal abrasion | - | - | - | - | - | 1 (0.0%) | 1 (0.0%) |
| Corneal disorder | - | - | - | - | - | 2 (0.0%) | 2 (0.0%) |
| Coronary arterial stent insertion | - | - | - | - | - | 1 (0.0%) | 1 (0.0%) |
| Coronary artery disease | - | - | - | - | - | 5 (0.0%) | 5 (0.0%) |
| Coronary artery occlusion | - | - | - | - | - | 6 (0.0%) | 6 (0.0%) |
| Corrective lens user | - | - | - | - | - | 1 (0.0%) | 1 (0.0%) |
| Cortisol decreased | - | - | - | - | - | 1 (0.0%) | 1 (0.0%) |
| Craniocerebral injury | - | - | - | - | - | 2 (0.0%) | 2 (0.0%) |
| Craniofacial fracture | - | - | - | - | - | 7 (0.0%) | 7 (0.0%) |
| Crohn's disease | - | - | - | - | - | 7 (0.0%) | 7 (0.0%) |
| Crush injury | - | - | - | - | - | 1 (0.0%) | 1 (0.0%) |
| CSF test abnormal | - | - | - | - | - | 1 (0.0%) | 1 (0.0%) |
| Cyanosis central | - | - | - | - | - | 1 (0.0%) | 1 (0.0%) |
| Cyclothymic disorder | - | - | - | - | - | 1 (0.0%) | 1 (0.0%) |
| Cystitis interstitial | - | - | - | - | - | 2 (0.0%) | 2 (0.0%) |
| Deafness | - | - | - | - | - | 6 (0.0%) | 6 (0.0%) |
| Deafness transitory | - | - | - | - | - | 1 (0.0%) | 1 (0.0%) |
| Decreased immune responsiveness | - | - | - | - | - | 3 (0.0%) | 3 (0.0%) |
| Deformity | - | - | - | - | - | 1 (0.0%) | 1 (0.0%) |
| Dehydroepiandrosterone decreased | - | - | - | - | - | 1 (0.0%) | 1 (0.0%) |
| Delirium tremens | - | - | - | - | - | 2 (0.0%) | 2 (0.0%) |
| Delusion of grandeur | - | - | - | - | - | 1 (0.0%) | 1 (0.0%) |
| Delusional disorder, unspecified type | - | - | - | - | - | 1 (0.0%) | 1 (0.0%) |
| Dementia | - | - | - | - | - | 28 (0.1%) | 28 (0.1%) |
| Demyelinating polyneuropathy | - | - | - | - | - | 2 (0.0%) | 2 (0.0%) |
| Demyelination | - | - | - | - | - | 2 (0.0%) | 2 (0.0%) |
| Dental restoration failure | - | - | - | - | - | 1 (0.0%) | 1 (0.0%) |
| Dependence on respirator | - | - | - | - | - | 1 (0.0%) | 1 (0.0%) |
| Depersonalisation/derealisation disorder | - | - | - | - | - | 4 (0.0%) | 4 (0.0%) |
| Depression suicidal | - | - | - | - | - | 1 (0.0%) | 1 (0.0%) |
| Depressive symptom | - | - | - | - | - | 2 (0.0%) | 2 (0.0%) |
| Dermal absorption increased | - | - | - | - | - | 1 (0.0%) | 1 (0.0%) |
| Dermal cyst | - | - | - | - | - | 2 (0.0%) | 2 (0.0%) |
| Dermatitis | - | - | - | - | - | 2 (0.0%) | 2 (0.0%) |
| Dermatitis acneiform | - | - | - | - | - | 2 (0.0%) | 2 (0.0%) |
| Dermatitis contact | - | - | - | - | - | 29 (0.1%) | 29 (0.1%) |
| Dermatitis psoriasiform | - | - | - | - | - | 1 (0.0%) | 1 (0.0%) |
| Detoxification | - | - | - | - | - | 1 (0.0%) | 1 (0.0%) |
| Developmental delay | - | - | - | - | - | 8 (0.0%) | 8 (0.0%) |
| Device breakage | - | - | - | - | - | 1 (0.0%) | 1 (0.0%) |
| Device dislocation | - | - | - | - | - | 2 (0.0%) | 2 (0.0%) |
| Device failure | - | - | - | - | - | 10 (0.0%) | 10 (0.0%) |
| Device kink | - | - | - | - | - | 1 (0.0%) | 1 (0.0%) |
| Device leakage | - | - | - | - | - | 34 (0.1%) | 34 (0.1%) |
| Device malfunction | - | - | - | - | - | 4 (0.0%) | 4 (0.0%) |
| Device occlusion | - | - | - | - | - | 4 (0.0%) | 4 (0.0%) |
| Diabetes insipidus | - | - | - | - | - | 1 (0.0%) | 1 (0.0%) |
| Diabetes mellitus inadequate control | - | - | - | - | - | 5 (0.0%) | 5 (0.0%) |
| Diabetic coma | - | - | - | - | - | 1 (0.0%) | 1 (0.0%) |
| Diabetic eye disease | - | - | - | - | - | 1 (0.0%) | 1 (0.0%) |
| Diabetic foot | - | - | - | - | - | 6 (0.0%) | 6 (0.0%) |
| Diabetic neuropathy | - | - | - | - | - | 8 (0.0%) | 8 (0.0%) |
| Diaphragmatic paralysis | - | - | - | - | - | 1 (0.0%) | 1 (0.0%) |
| Diet refusal | - | - | - | - | - | 5 (0.0%) | 5 (0.0%) |
| Dilated cardiomyopathy | - | - | - | - | - | 1 (0.0%) | 1 (0.0%) |
| Diplopia | - | - | - | - | - | 19 (0.1%) | 19 (0.1%) |
| Discoloured vomit | - | - | - | - | - | 1 (0.0%) | 1 (0.0%) |
| Disinhibition | - | - | - | - | - | 1 (0.0%) | 1 (0.0%) |
| Dislocation of vertebra | - | - | - | - | - | 1 (0.0%) | 1 (0.0%) |
| Disorganised speech | - | - | - | - | - | 3 (0.0%) | 3 (0.0%) |
| Dissociative disorder | - | - | - | - | - | 1 (0.0%) | 1 (0.0%) |
| Distractibility | - | - | - | - | - | 1 (0.0%) | 1 (0.0%) |
| Disturbance in social behaviour | - | - | - | - | - | 2 (0.0%) | 2 (0.0%) |
| Diverticulum | - | - | - | - | - | 3 (0.0%) | 3 (0.0%) |
| Diverticulum intestinal | - | - | - | - | - | 1 (0.0%) | 1 (0.0%) |
| Dizziness postural | - | - | - | - | - | 4 (0.0%) | 4 (0.0%) |
| Drowning | - | - | - | - | - | 2 (0.0%) | 2 (0.0%) |
| Drug-induced liver injury | - | - | - | - | - | 1 (0.0%) | 1 (0.0%) |
| Drug delivery system issue | - | - | - | - | - | 2 (0.0%) | 2 (0.0%) |
| Drug delivery system malfunction | - | - | - | - | - | 1 (0.0%) | 1 (0.0%) |
| Drug effect faster than expected | - | - | - | - | - | 2 (0.0%) | 2 (0.0%) |
| Drug effect less than expected | - | - | - | - | - | 1 (0.0%) | 1 (0.0%) |
| Drug eruption | - | - | - | - | - | 1 (0.0%) | 1 (0.0%) |
| Drug metabolising enzyme increased | - | - | - | - | - | 1 (0.0%) | 1 (0.0%) |
| Drug monitoring procedure not performed | - | - | - | - | - | 1 (0.0%) | 1 (0.0%) |
| Drug screen negative | - | - | - | - | - | 7 (0.0%) | 7 (0.0%) |
| Drug tolerance increased | - | - | - | - | - | 3 (0.0%) | 3 (0.0%) |
| Drug withdrawal headache | - | - | - | - | - | 1 (0.0%) | 1 (0.0%) |
| Dry eye | - | - | - | - | - | 5 (0.0%) | 5 (0.0%) |
| Dry skin | - | - | - | - | - | 16 (0.1%) | 16 (0.0%) |
| Dry throat | - | - | - | - | - | 2 (0.0%) | 2 (0.0%) |
| Dumping syndrome | - | - | - | - | - | 1 (0.0%) | 1 (0.0%) |
| Duodenitis | - | - | - | - | - | 1 (0.0%) | 1 (0.0%) |
| Duplicate therapy error | - | - | - | - | - | 2 (0.0%) | 2 (0.0%) |
| Dyschezia | - | - | - | - | - | 2 (0.0%) | 2 (0.0%) |
| Dyschromatopsia | - | - | - | - | - | 1 (0.0%) | 1 (0.0%) |
| Dysgraphia | - | - | - | - | - | 5 (0.0%) | 5 (0.0%) |
| Dyslexia | - | - | - | - | - | 2 (0.0%) | 2 (0.0%) |
| Dyslipidaemia | - | - | - | - | - | 2 (0.0%) | 2 (0.0%) |
| Dysphemia | - | - | - | - | - | 5 (0.0%) | 5 (0.0%) |
| Dysphoria | - | - | - | - | - | 9 (0.0%) | 9 (0.0%) |
| Dyspnoea at rest | - | - | - | - | - | 1 (0.0%) | 1 (0.0%) |
| Dyspraxia | - | - | - | - | - | 1 (0.0%) | 1 (0.0%) |
| Dysstasia | - | - | - | - | - | 27 (0.1%) | 27 (0.1%) |
| Ear disorder | - | - | - | - | - | 1 (0.0%) | 1 (0.0%) |
| Ear infection | - | - | - | - | - | 3 (0.0%) | 3 (0.0%) |
| Ear injury | - | - | - | - | - | 1 (0.0%) | 1 (0.0%) |
| Ear pain | - | - | - | - | - | 2 (0.0%) | 2 (0.0%) |
| Eating disorder symptom | - | - | - | - | - | 1 (0.0%) | 1 (0.0%) |
| Ecchymosis | - | - | - | - | - | 2 (0.0%) | 2 (0.0%) |
| Echocardiogram abnormal | - | - | - | - | - | 1 (0.0%) | 1 (0.0%) |
| Economic problem | - | - | - | - | - | 1 (0.0%) | 1 (0.0%) |
| Eczema | - | - | - | - | - | 3 (0.0%) | 3 (0.0%) |
| Ejaculation delayed | - | - | - | - | - | 1 (0.0%) | 1 (0.0%) |
| Ejaculation disorder | - | - | - | - | - | 1 (0.0%) | 1 (0.0%) |
| Elderly | - | - | - | - | - | 1 (0.0%) | 1 (0.0%) |
| Electric shock sensation | - | - | - | - | - | 6 (0.0%) | 6 (0.0%) |
| Electrocardiogram change | - | - | - | - | - | 1 (0.0%) | 1 (0.0%) |
| Electroconvulsive therapy | - | - | - | - | - | 1 (0.0%) | 1 (0.0%) |
| Electrolyte depletion | - | - | - | - | - | 2 (0.0%) | 2 (0.0%) |
| Embolic cerebral infarction | - | - | - | - | - | 1 (0.0%) | 1 (0.0%) |
| Emergency care | - | - | - | - | - | 1 (0.0%) | 1 (0.0%) |
| Emphysema | - | - | - | - | - | 7 (0.0%) | 7 (0.0%) |
| Encephalitis | - | - | - | - | - | 1 (0.0%) | 1 (0.0%) |
| Endocrine disorder | - | - | - | - | - | 5 (0.0%) | 5 (0.0%) |
| Endometrial cancer | - | - | - | - | - | 1 (0.0%) | 1 (0.0%) |
| Energy increased | - | - | - | - | - | 1 (0.0%) | 1 (0.0%) |
| Enteritis infectious | - | - | - | - | - | 1 (0.0%) | 1 (0.0%) |
| Enuresis | - | - | - | - | - | 4 (0.0%) | 4 (0.0%) |
| Enzyme abnormality | - | - | - | - | - | 2 (0.0%) | 2 (0.0%) |
| Epicondylitis | - | - | - | - | - | 1 (0.0%) | 1 (0.0%) |
| Eructation | - | - | - | - | - | 2 (0.0%) | 2 (0.0%) |
| Escherichia infection | - | - | - | - | - | 2 (0.0%) | 2 (0.0%) |
| Essential hypertension | - | - | - | - | - | 1 (0.0%) | 1 (0.0%) |
| Executive dysfunction | - | - | - | - | - | 3 (0.0%) | 3 (0.0%) |
| Exophthalmos | - | - | - | - | - | 1 (0.0%) | 1 (0.0%) |
| Exostosis | - | - | - | - | - | 5 (0.0%) | 5 (0.0%) |
| Expired product administered | - | - | - | - | - | 16 (0.1%) | 16 (0.0%) |
| Exposure to extreme temperature | - | - | - | - | - | 10 (0.0%) | 10 (0.0%) |
| Exposure to toxic agent | - | - | - | - | - | 1 (0.0%) | 1 (0.0%) |
| Extradural abscess | - | - | - | - | - | 2 (0.0%) | 2 (0.0%) |
| Extrasystoles | - | - | - | - | - | 1 (0.0%) | 1 (0.0%) |
| Eye contusion | - | - | - | - | - | 1 (0.0%) | 1 (0.0%) |
| Eye disorder | - | - | - | - | - | 6 (0.0%) | 6 (0.0%) |
| Eye haemorrhage | - | - | - | - | - | 1 (0.0%) | 1 (0.0%) |
| Eye infection | - | - | - | - | - | 1 (0.0%) | 1 (0.0%) |
| Eye injury | - | - | - | - | - | 2 (0.0%) | 2 (0.0%) |
| Eye irritation | - | - | - | - | - | 5 (0.0%) | 5 (0.0%) |
| Eye oedema | - | - | - | - | - | 2 (0.0%) | 2 (0.0%) |
| Eye pain | - | - | - | - | - | 5 (0.0%) | 5 (0.0%) |
| Eyelid disorder | - | - | - | - | - | 1 (0.0%) | 1 (0.0%) |
| Facial nerve disorder | - | - | - | - | - | 1 (0.0%) | 1 (0.0%) |
| Facial operation | - | - | - | - | - | 1 (0.0%) | 1 (0.0%) |
| Facial pain | - | - | - | - | - | 1 (0.0%) | 1 (0.0%) |
| Facial wasting | - | - | - | - | - | 1 (0.0%) | 1 (0.0%) |
| Faeces hard | - | - | - | - | - | 2 (0.0%) | 2 (0.0%) |
| Failure to thrive | - | - | - | - | - | 5 (0.0%) | 5 (0.0%) |
| False negative investigation result | - | - | - | - | - | 1 (0.0%) | 1 (0.0%) |
| Fat embolism | - | - | - | - | - | 1 (0.0%) | 1 (0.0%) |
| Fear of death | - | - | - | - | - | 2 (0.0%) | 2 (0.0%) |
| Fear of injection | - | - | - | - | - | 1 (0.0%) | 1 (0.0%) |
| Febrile neutropenia | - | - | - | - | - | 4 (0.0%) | 4 (0.0%) |
| Feeling guilty | - | - | - | - | - | 5 (0.0%) | 5 (0.0%) |
| Feeling of body temperature change | - | - | - | - | - | 16 (0.1%) | 16 (0.0%) |
| Feeling of despair | - | - | - | - | - | 2 (0.0%) | 2 (0.0%) |
| Femoral hernia incarcerated | - | - | - | - | - | 1 (0.0%) | 1 (0.0%) |
| Femur fracture | - | - | - | - | - | 7 (0.0%) | 7 (0.0%) |
| Fibromyalgia | - | - | - | - | - | 11 (0.0%) | 11 (0.0%) |
| Fine motor skill dysfunction | - | - | - | - | - | 1 (0.0%) | 1 (0.0%) |
| Fistula | - | - | - | - | - | 2 (0.0%) | 2 (0.0%) |
| Flank pain | - | - | - | - | - | 2 (0.0%) | 2 (0.0%) |
| Flatulence | - | - | - | - | - | 10 (0.0%) | 10 (0.0%) |
| Fluid intake reduced | - | - | - | - | - | 3 (0.0%) | 3 (0.0%) |
| Fluid retention | - | - | - | - | - | 10 (0.0%) | 10 (0.0%) |
| Foaming at mouth | - | - | - | - | - | 4 (0.0%) | 4 (0.0%) |
| Focal dyscognitive seizures | - | - | - | - | - | 2 (0.0%) | 2 (0.0%) |
| Folliculitis | - | - | - | - | - | 1 (0.0%) | 1 (0.0%) |
| Foot deformity | - | - | - | - | - | 2 (0.0%) | 2 (0.0%) |
| Foreign body | - | - | - | - | - | 1 (0.0%) | 1 (0.0%) |
| Foreign body in eye | - | - | - | - | - | 1 (0.0%) | 1 (0.0%) |
| Foreign body in gastrointestinal tract | - | - | - | - | - | 1 (0.0%) | 1 (0.0%) |
| Foreign body in throat | - | - | - | - | - | 2 (0.0%) | 2 (0.0%) |
| Foreign body reaction | - | - | - | - | - | 1 (0.0%) | 1 (0.0%) |
| Fracture | - | - | - | - | - | 16 (0.1%) | 16 (0.0%) |
| Fractured coccyx | - | - | - | - | - | 1 (0.0%) | 1 (0.0%) |
| Fractured sacrum | - | - | - | - | - | 1 (0.0%) | 1 (0.0%) |
| Frontotemporal dementia | - | - | - | - | - | 1 (0.0%) | 1 (0.0%) |
| Full blood count abnormal | - | - | - | - | - | 2 (0.0%) | 2 (0.0%) |
| Full blood count decreased | - | - | - | - | - | 2 (0.0%) | 2 (0.0%) |
| Functional gastrointestinal disorder | - | - | - | - | - | 18 (0.1%) | 18 (0.1%) |
| Fungaemia | - | - | - | - | - | 1 (0.0%) | 1 (0.0%) |
| Fungal rhinitis | - | - | - | - | - | 1 (0.0%) | 1 (0.0%) |
| Gallbladder cancer stage IV | - | - | - | - | - | 1 (0.0%) | 1 (0.0%) |
| Gallbladder operation | - | - | - | - | - | 5 (0.0%) | 5 (0.0%) |
| Gallbladder rupture | - | - | - | - | - | 1 (0.0%) | 1 (0.0%) |
| Gamma-glutamyltransferase increased | - | - | - | - | - | 4 (0.0%) | 4 (0.0%) |
| Gangrene | - | - | - | - | - | 5 (0.0%) | 5 (0.0%) |
| Gastric bypass | - | - | - | - | - | 1 (0.0%) | 1 (0.0%) |
| Gastric dilatation | - | - | - | - | - | 12 (0.0%) | 12 (0.0%) |
| Gastric polyps | - | - | - | - | - | 1 (0.0%) | 1 (0.0%) |
| Gastric ulcer haemorrhage | - | - | - | - | - | 1 (0.0%) | 1 (0.0%) |
| Gastritis erosive | - | - | - | - | - | 1 (0.0%) | 1 (0.0%) |
| Gastroenteritis | - | - | - | - | - | 4 (0.0%) | 4 (0.0%) |
| Gastroenteritis salmonella | - | - | - | - | - | 1 (0.0%) | 1 (0.0%) |
| Gastroenteritis viral | - | - | - | - | - | 5 (0.0%) | 5 (0.0%) |
| Gastrointestinal erosion | - | - | - | - | - | 2 (0.0%) | 2 (0.0%) |
| Gastrointestinal infection | - | - | - | - | - | 3 (0.0%) | 3 (0.0%) |
| Gastrointestinal inflammation | - | - | - | - | - | 1 (0.0%) | 1 (0.0%) |
| Gastrointestinal motility disorder | - | - | - | - | - | 8 (0.0%) | 8 (0.0%) |
| Gastrointestinal pain | - | - | - | - | - | 2 (0.0%) | 2 (0.0%) |
| Gastrointestinal sounds abnormal | - | - | - | - | - | 1 (0.0%) | 1 (0.0%) |
| Gastrointestinal toxicity | - | - | - | - | - | 1 (0.0%) | 1 (0.0%) |
| General physical condition abnormal | - | - | - | - | - | 3 (0.0%) | 3 (0.0%) |
| Generalised anxiety disorder | - | - | - | - | - | 2 (0.0%) | 2 (0.0%) |
| Generalised oedema | - | - | - | - | - | 3 (0.0%) | 3 (0.0%) |
| Generalised onset non-motor seizure | - | - | - | - | - | 2 (0.0%) | 2 (0.0%) |
| Giant cell arteritis | - | - | - | - | - | 1 (0.0%) | 1 (0.0%) |
| Gitelman's syndrome | - | - | - | - | - | 1 (0.0%) | 1 (0.0%) |
| Glare | - | - | - | - | - | 1 (0.0%) | 1 (0.0%) |
| Gliosis | - | - | - | - | - | 1 (0.0%) | 1 (0.0%) |
| Globulins increased | - | - | - | - | - | 1 (0.0%) | 1 (0.0%) |
| Glomerulonephritis | - | - | - | - | - | 1 (0.0%) | 1 (0.0%) |
| Glucose tolerance impaired | - | - | - | - | - | 2 (0.0%) | 2 (0.0%) |
| Goitre | - | - | - | - | - | 1 (0.0%) | 1 (0.0%) |
| Gout | - | - | - | - | - | 3 (0.0%) | 3 (0.0%) |
| Gouty arthritis | - | - | - | - | - | 1 (0.0%) | 1 (0.0%) |
| Gravitational oedema | - | - | - | - | - | 1 (0.0%) | 1 (0.0%) |
| Greater trochanteric pain syndrome | - | - | - | - | - | 1 (0.0%) | 1 (0.0%) |
| Groin infection | - | - | - | - | - | 1 (0.0%) | 1 (0.0%) |
| Gross motor delay | - | - | - | - | - | 1 (0.0%) | 1 (0.0%) |
| Haemangioma | - | - | - | - | - | 1 (0.0%) | 1 (0.0%) |
| Haemarthrosis | - | - | - | - | - | 2 (0.0%) | 2 (0.0%) |
| Haematoma | - | - | - | - | - | 2 (0.0%) | 2 (0.0%) |
| Haemorrhage intracranial | - | - | - | - | - | 2 (0.0%) | 2 (0.0%) |
| Haemorrhagic pneumonia | - | - | - | - | - | 1 (0.0%) | 1 (0.0%) |
| Haemorrhagic stroke | - | - | - | - | - | 1 (0.0%) | 1 (0.0%) |
| Haemorrhoidal haemorrhage | - | - | - | - | - | 1 (0.0%) | 1 (0.0%) |
| Hair disorder | - | - | - | - | - | 1 (0.0%) | 1 (0.0%) |
| Hallucinations, mixed | - | - | - | - | - | 8 (0.0%) | 8 (0.0%) |
| Hand deformity | - | - | - | - | - | 1 (0.0%) | 1 (0.0%) |
| Hand fracture | - | - | - | - | - | 5 (0.0%) | 5 (0.0%) |
| Heart rate abnormal | - | - | - | - | - | 5 (0.0%) | 5 (0.0%) |
| Heart valve incompetence | - | - | - | - | - | 2 (0.0%) | 2 (0.0%) |
| Heat exhaustion | - | - | - | - | - | 1 (0.0%) | 1 (0.0%) |
| Heat stroke | - | - | - | - | - | 3 (0.0%) | 3 (0.0%) |
| Heavy menstrual bleeding | - | - | - | - | - | 2 (0.0%) | 2 (0.0%) |
| Hepatic cancer | - | - | - | - | - | 3 (0.0%) | 3 (0.0%) |
| Hepatic cirrhosis | - | - | - | - | - | 2 (0.0%) | 2 (0.0%) |
| Hepatic cyst | - | - | - | - | - | 1 (0.0%) | 1 (0.0%) |
| Hepatic infection | - | - | - | - | - | 1 (0.0%) | 1 (0.0%) |
| Hepatic lesion | - | - | - | - | - | 1 (0.0%) | 1 (0.0%) |
| Hepatic mass | - | - | - | - | - | 2 (0.0%) | 2 (0.0%) |
| Hepatic pain | - | - | - | - | - | 1 (0.0%) | 1 (0.0%) |
| Hepatitis acute | - | - | - | - | - | 1 (0.0%) | 1 (0.0%) |
| Hepatitis C antibody positive | - | - | - | - | - | 1 (0.0%) | 1 (0.0%) |
| Hepatobiliary disease | - | - | - | - | - | 1 (0.0%) | 1 (0.0%) |
| Hepatocellular injury | - | - | - | - | - | 1 (0.0%) | 1 (0.0%) |
| Hepatotoxicity | - | - | - | - | - | 1 (0.0%) | 1 (0.0%) |
| Hernia | - | - | - | - | - | 4 (0.0%) | 4 (0.0%) |
| Hernia hiatus repair | - | - | - | - | - | 1 (0.0%) | 1 (0.0%) |
| Hernia repair | - | - | - | - | - | 1 (0.0%) | 1 (0.0%) |
| Hiatus hernia | - | - | - | - | - | 9 (0.0%) | 9 (0.0%) |
| Hip fracture | - | - | - | - | - | 13 (0.0%) | 13 (0.0%) |
| Hip surgery | - | - | - | - | - | 4 (0.0%) | 4 (0.0%) |
| HIV infection | - | - | - | - | - | 1 (0.0%) | 1 (0.0%) |
| Homicidal ideation | - | - | - | - | - | 2 (0.0%) | 2 (0.0%) |
| Homicide | - | - | - | - | - | 1 (0.0%) | 1 (0.0%) |
| Hormone level abnormal | - | - | - | - | - | 4 (0.0%) | 4 (0.0%) |
| Hostility | - | - | - | - | - | 2 (0.0%) | 2 (0.0%) |
| Humerus fracture | - | - | - | - | - | 3 (0.0%) | 3 (0.0%) |
| Hyperacusis | - | - | - | - | - | 5 (0.0%) | 5 (0.0%) |
| Hypercapnic coma | - | - | - | - | - | 9 (0.0%) | 9 (0.0%) |
| Hypercoagulation | - | - | - | - | - | 1 (0.0%) | 1 (0.0%) |
| Hyperlipidaemia | - | - | - | - | - | 3 (0.0%) | 3 (0.0%) |
| Hypermagnesaemia | - | - | - | - | - | 1 (0.0%) | 1 (0.0%) |
| Hyperphagia | - | - | - | - | - | 1 (0.0%) | 1 (0.0%) |
| Hyperproteinaemia | - | - | - | - | - | 1 (0.0%) | 1 (0.0%) |
| Hyperthyroidism | - | - | - | - | - | 1 (0.0%) | 1 (0.0%) |
| Hypoacusis | - | - | - | - | - | 12 (0.0%) | 12 (0.0%) |
| Hypoalbuminaemia | - | - | - | - | - | 4 (0.0%) | 4 (0.0%) |
| Hypochloraemia | - | - | - | - | - | 1 (0.0%) | 1 (0.0%) |
| Hypoglycaemic coma | - | - | - | - | - | 1 (0.0%) | 1 (0.0%) |
| Hypoglycaemic seizure | - | - | - | - | - | 1 (0.0%) | 1 (0.0%) |
| Hypoglycaemic unconsciousness | - | - | - | - | - | 1 (0.0%) | 1 (0.0%) |
| Hypogonadism | - | - | - | - | - | 2 (0.0%) | 2 (0.0%) |
| Hypopharyngeal cancer | - | - | - | - | - | 1 (0.0%) | 1 (0.0%) |
| Hypothalamo-pituitary disorder | - | - | - | - | - | 4 (0.0%) | 4 (0.0%) |
| Hypovitaminosis | - | - | - | - | - | 3 (0.0%) | 3 (0.0%) |
| Hysterectomy | - | - | - | - | - | 4 (0.0%) | 4 (0.0%) |
| Idiopathic intracranial hypertension | - | - | - | - | - | 1 (0.0%) | 1 (0.0%) |
| Ill-defined disorder | - | - | - | - | - | 8 (0.0%) | 8 (0.0%) |
| Illusion | - | - | - | - | - | 1 (0.0%) | 1 (0.0%) |
| Immobile | - | - | - | - | - | 1 (0.0%) | 1 (0.0%) |
| Immune system disorder | - | - | - | - | - | 2 (0.0%) | 2 (0.0%) |
| Impaired driving ability | - | - | - | - | - | 14 (0.0%) | 14 (0.0%) |
| Impaired healing | - | - | - | - | - | 3 (0.0%) | 3 (0.0%) |
| Impaired quality of life | - | - | - | - | - | 2 (0.0%) | 2 (0.0%) |
| Impatience | - | - | - | - | - | 1 (0.0%) | 1 (0.0%) |
| Implant site erythema | - | - | - | - | - | 1 (0.0%) | 1 (0.0%) |
| Implant site extravasation | - | - | - | - | - | 1 (0.0%) | 1 (0.0%) |
| Implant site infection | - | - | - | - | - | 1 (0.0%) | 1 (0.0%) |
| Implant site mass | - | - | - | - | - | 1 (0.0%) | 1 (0.0%) |
| Implant site pruritus | - | - | - | - | - | 1 (0.0%) | 1 (0.0%) |
| Inadequate diet | - | - | - | - | - | 2 (0.0%) | 2 (0.0%) |
| Inappropriate antidiuretic hormone secretion | - | - | - | - | - | 2 (0.0%) | 2 (0.0%) |
| Inappropriate release of product for distribution | - | - | - | - | - | 1 (0.0%) | 1 (0.0%) |
| Incisional drainage | - | - | - | - | - | 1 (0.0%) | 1 (0.0%) |
| Incontinence | - | - | - | - | - | 8 (0.0%) | 8 (0.0%) |
| Incorrect disposal of product | - | - | - | - | - | 1 (0.0%) | 1 (0.0%) |
| Incorrect dose administered by device | - | - | - | - | - | 2 (0.0%) | 2 (0.0%) |
| Increased appetite | - | - | - | - | - | 1 (0.0%) | 1 (0.0%) |
| Increased tendency to bruise | - | - | - | - | - | 1 (0.0%) | 1 (0.0%) |
| Indifference | - | - | - | - | - | 1 (0.0%) | 1 (0.0%) |
| Infarction | - | - | - | - | - | 2 (0.0%) | 2 (0.0%) |
| Infected skin ulcer | - | - | - | - | - | 1 (0.0%) | 1 (0.0%) |
| Inflammation | - | - | - | - | - | 5 (0.0%) | 5 (0.0%) |
| Inflammatory marker increased | - | - | - | - | - | 1 (0.0%) | 1 (0.0%) |
| Infusion site mass | - | - | - | - | - | 3 (0.0%) | 3 (0.0%) |
| Infusion site reaction | - | - | - | - | - | 1 (0.0%) | 1 (0.0%) |
| Inhibitory drug interaction | - | - | - | - | - | 1 (0.0%) | 1 (0.0%) |
| Initial insomnia | - | - | - | - | - | 5 (0.0%) | 5 (0.0%) |
| Insulin-like growth factor decreased | - | - | - | - | - | 1 (0.0%) | 1 (0.0%) |
| Intentional self-injury | - | - | - | - | - | 3 (0.0%) | 3 (0.0%) |
| Intercepted medication error | - | - | - | - | - | 1 (0.0%) | 1 (0.0%) |
| Internal injury | - | - | - | - | - | 1 (0.0%) | 1 (0.0%) |
| International normalised ratio abnormal | - | - | - | - | - | 2 (0.0%) | 2 (0.0%) |
| International normalised ratio increased | - | - | - | - | - | 7 (0.0%) | 7 (0.0%) |
| Intervertebral disc compression | - | - | - | - | - | 2 (0.0%) | 2 (0.0%) |
| Intervertebral disc degeneration | - | - | - | - | - | 17 (0.1%) | 17 (0.0%) |
| Intervertebral disc disorder | - | - | - | - | - | 8 (0.0%) | 8 (0.0%) |
| Intervertebral disc displacement | - | - | - | - | - | 1 (0.0%) | 1 (0.0%) |
| Intervertebral disc injury | - | - | - | - | - | 1 (0.0%) | 1 (0.0%) |
| Intervertebral disc operation | - | - | - | - | - | 1 (0.0%) | 1 (0.0%) |
| Intervertebral discitis | - | - | - | - | - | 2 (0.0%) | 2 (0.0%) |
| Intestinal dilatation | - | - | - | - | - | 1 (0.0%) | 1 (0.0%) |
| Intestinal gangrene | - | - | - | - | - | 2 (0.0%) | 2 (0.0%) |
| Intestinal haemorrhage | - | - | - | - | - | 2 (0.0%) | 2 (0.0%) |
| Intestinal stenosis | - | - | - | - | - | 1 (0.0%) | 1 (0.0%) |
| Intracardiac thrombus | - | - | - | - | - | 1 (0.0%) | 1 (0.0%) |
| Intracranial haematoma | - | - | - | - | - | 1 (0.0%) | 1 (0.0%) |
| Intracranial hypotension | - | - | - | - | - | 1 (0.0%) | 1 (0.0%) |
| Iron deficiency anaemia | - | - | - | - | - | 5 (0.0%) | 5 (0.0%) |
| Irritable bowel syndrome | - | - | - | - | - | 7 (0.0%) | 7 (0.0%) |
| Ischaemia | - | - | - | - | - | 1 (0.0%) | 1 (0.0%) |
| Ischaemic hepatitis | - | - | - | - | - | 2 (0.0%) | 2 (0.0%) |
| Jaw fracture | - | - | - | - | - | 2 (0.0%) | 2 (0.0%) |
| Joint dislocation | - | - | - | - | - | 6 (0.0%) | 6 (0.0%) |
| Joint effusion | - | - | - | - | - | 1 (0.0%) | 1 (0.0%) |
| Joint injury | - | - | - | - | - | 9 (0.0%) | 9 (0.0%) |
| Joint noise | - | - | - | - | - | 1 (0.0%) | 1 (0.0%) |
| Joint range of motion decreased | - | - | - | - | - | 2 (0.0%) | 2 (0.0%) |
| Joint swelling | - | - | - | - | - | 10 (0.0%) | 10 (0.0%) |
| Kinesiophobia | - | - | - | - | - | 1 (0.0%) | 1 (0.0%) |
| Knee operation | - | - | - | - | - | 3 (0.0%) | 3 (0.0%) |
| Kyphosis | - | - | - | - | - | 2 (0.0%) | 2 (0.0%) |
| Labelled drug-disease interaction medication error | - | - | - | - | - | 1 (0.0%) | 1 (0.0%) |
| Labelled drug-drug interaction issue | - | - | - | - | - | 1 (0.0%) | 1 (0.0%) |
| Labelled drug-food interaction medication error | - | - | - | - | - | 1 (0.0%) | 1 (0.0%) |
| Labyrinthitis | - | - | - | - | - | 1 (0.0%) | 1 (0.0%) |
| Lactose intolerance | - | - | - | - | - | 1 (0.0%) | 1 (0.0%) |
| Lacunar infarction | - | - | - | - | - | 2 (0.0%) | 2 (0.0%) |
| Lagophthalmos | - | - | - | - | - | 1 (0.0%) | 1 (0.0%) |
| Laparotomy | - | - | - | - | - | 1 (0.0%) | 1 (0.0%) |
| Large intestinal obstruction | - | - | - | - | - | 1 (0.0%) | 1 (0.0%) |
| Large intestine perforation | - | - | - | - | - | 2 (0.0%) | 2 (0.0%) |
| Large intestine polyp | - | - | - | - | - | 4 (0.0%) | 4 (0.0%) |
| Laryngeal cancer | - | - | - | - | - | 1 (0.0%) | 1 (0.0%) |
| Laryngeal dyspnoea | - | - | - | - | - | 1 (0.0%) | 1 (0.0%) |
| Laryngeal inflammation | - | - | - | - | - | 1 (0.0%) | 1 (0.0%) |
| Laryngeal pain | - | - | - | - | - | 1 (0.0%) | 1 (0.0%) |
| Laryngitis | - | - | - | - | - | 1 (0.0%) | 1 (0.0%) |
| Learning disability | - | - | - | - | - | 9 (0.0%) | 9 (0.0%) |
| Left ventricular dysfunction | - | - | - | - | - | 1 (0.0%) | 1 (0.0%) |
| Leukaemia | - | - | - | - | - | 2 (0.0%) | 2 (0.0%) |
| Leukaemia recurrent | - | - | - | - | - | 1 (0.0%) | 1 (0.0%) |
| Leukoencephalopathy | - | - | - | - | - | 14 (0.0%) | 14 (0.0%) |
| Leukopenia | - | - | - | - | - | 2 (0.0%) | 2 (0.0%) |
| Lichenoid keratosis | - | - | - | - | - | 1 (0.0%) | 1 (0.0%) |
| Life expectancy shortened | - | - | - | - | - | 2 (0.0%) | 2 (0.0%) |
| Ligament operation | - | - | - | - | - | 1 (0.0%) | 1 (0.0%) |
| Ligament rupture | - | - | - | - | - | 1 (0.0%) | 1 (0.0%) |
| Limb crushing injury | - | - | - | - | - | 1 (0.0%) | 1 (0.0%) |
| Limb injury | - | - | - | - | - | 11 (0.0%) | 11 (0.0%) |
| Limb operation | - | - | - | - | - | 1 (0.0%) | 1 (0.0%) |
| Lip and/or oral cavity cancer | - | - | - | - | - | 1 (0.0%) | 1 (0.0%) |
| Lip disorder | - | - | - | - | - | 1 (0.0%) | 1 (0.0%) |
| Lip dry | - | - | - | - | - | 1 (0.0%) | 1 (0.0%) |
| Lip haemorrhage | - | - | - | - | - | 1 (0.0%) | 1 (0.0%) |
| Lip injury | - | - | - | - | - | 1 (0.0%) | 1 (0.0%) |
| Lip swelling | - | - | - | - | - | 1 (0.0%) | 1 (0.0%) |
| Lipase increased | - | - | - | - | - | 2 (0.0%) | 2 (0.0%) |
| Lipoma | - | - | - | - | - | 1 (0.0%) | 1 (0.0%) |
| Listless | - | - | - | - | - | 5 (0.0%) | 5 (0.0%) |
| Livedo reticularis | - | - | - | - | - | 1 (0.0%) | 1 (0.0%) |
| Liver function test increased | - | - | - | - | - | 4 (0.0%) | 4 (0.0%) |
| Liver injury | - | - | - | - | - | 1 (0.0%) | 1 (0.0%) |
| Living in residential institution | - | - | - | - | - | 1 (0.0%) | 1 (0.0%) |
| Localised infection | - | - | - | - | - | 2 (0.0%) | 2 (0.0%) |
| Logorrhoea | - | - | - | - | - | 4 (0.0%) | 4 (0.0%) |
| Loss of control of legs | - | - | - | - | - | 4 (0.0%) | 4 (0.0%) |
| Loss of libido | - | - | - | - | - | 3 (0.0%) | 3 (0.0%) |
| Lower limb fracture | - | - | - | - | - | 7 (0.0%) | 7 (0.0%) |
| Lower respiratory tract infection | - | - | - | - | - | 2 (0.0%) | 2 (0.0%) |
| Lumbar radiculopathy | - | - | - | - | - | 2 (0.0%) | 2 (0.0%) |
| Lumbar spinal stenosis | - | - | - | - | - | 4 (0.0%) | 4 (0.0%) |
| Lung abscess | - | - | - | - | - | 1 (0.0%) | 1 (0.0%) |
| Lung adenocarcinoma | - | - | - | - | - | 2 (0.0%) | 2 (0.0%) |
| Lung cancer metastatic | - | - | - | - | - | 4 (0.0%) | 4 (0.0%) |
| Lung carcinoma cell type unspecified stage IV | - | - | - | - | - | 1 (0.0%) | 1 (0.0%) |
| Lung lobectomy | - | - | - | - | - | 1 (0.0%) | 1 (0.0%) |
| Lung neoplasm | - | - | - | - | - | 2 (0.0%) | 2 (0.0%) |
| Lung squamous cell carcinoma recurrent | - | - | - | - | - | 1 (0.0%) | 1 (0.0%) |
| Lyme disease | - | - | - | - | - | 3 (0.0%) | 3 (0.0%) |
| Lymphangiosis carcinomatosa | - | - | - | - | - | 1 (0.0%) | 1 (0.0%) |
| Lymphoma | - | - | - | - | - | 2 (0.0%) | 2 (0.0%) |
| Magnetic resonance imaging head abnormal | - | - | - | - | - | 2 (0.0%) | 2 (0.0%) |
| Malignant ascites | - | - | - | - | - | 1 (0.0%) | 1 (0.0%) |
| Malignant peritoneal neoplasm | - | - | - | - | - | 1 (0.0%) | 1 (0.0%) |
| Malignant pleural effusion | - | - | - | - | - | 3 (0.0%) | 3 (0.0%) |
| Malignant polyp | - | - | - | - | - | 1 (0.0%) | 1 (0.0%) |
| Mammogram abnormal | - | - | - | - | - | 1 (0.0%) | 1 (0.0%) |
| Mania | - | - | - | - | - | 5 (0.0%) | 5 (0.0%) |
| Manufacturing production issue | - | - | - | - | - | 1 (0.0%) | 1 (0.0%) |
| Marital problem | - | - | - | - | - | 1 (0.0%) | 1 (0.0%) |
| Mass | - | - | - | - | - | 1 (0.0%) | 1 (0.0%) |
| Mastectomy | - | - | - | - | - | 1 (0.0%) | 1 (0.0%) |
| Medical device implantation | - | - | - | - | - | 1 (0.0%) | 1 (0.0%) |
| Medical device site abscess | - | - | - | - | - | 1 (0.0%) | 1 (0.0%) |
| Melaena | - | - | - | - | - | 7 (0.0%) | 7 (0.0%) |
| Melanoma recurrent | - | - | - | - | - | 1 (0.0%) | 1 (0.0%) |
| Meniere's disease | - | - | - | - | - | 2 (0.0%) | 2 (0.0%) |
| Meningioma | - | - | - | - | - | 2 (0.0%) | 2 (0.0%) |
| Meningitis | - | - | - | - | - | 7 (0.0%) | 7 (0.0%) |
| Meniscus injury | - | - | - | - | - | 2 (0.0%) | 2 (0.0%) |
| Menopause | - | - | - | - | - | 3 (0.0%) | 3 (0.0%) |
| Mental fatigue | - | - | - | - | - | 1 (0.0%) | 1 (0.0%) |
| Meralgia paraesthetica | - | - | - | - | - | 1 (0.0%) | 1 (0.0%) |
| Metabolic disorder | - | - | - | - | - | 4 (0.0%) | 4 (0.0%) |
| Metabolic surgery | - | - | - | - | - | 1 (0.0%) | 1 (0.0%) |
| Metal poisoning | - | - | - | - | - | 1 (0.0%) | 1 (0.0%) |
| Metastases to gastrointestinal tract | - | - | - | - | - | 1 (0.0%) | 1 (0.0%) |
| Metastases to meninges | - | - | - | - | - | 2 (0.0%) | 2 (0.0%) |
| Metastases to pleura | - | - | - | - | - | 1 (0.0%) | 1 (0.0%) |
| Metastatic gastric cancer | - | - | - | - | - | 1 (0.0%) | 1 (0.0%) |
| Metastatic malignant melanoma | - | - | - | - | - | 1 (0.0%) | 1 (0.0%) |
| Metastatic neoplasm | - | - | - | - | - | 4 (0.0%) | 4 (0.0%) |
| Metastatic squamous cell carcinoma | - | - | - | - | - | 1 (0.0%) | 1 (0.0%) |
| Microcytic anaemia | - | - | - | - | - | 1 (0.0%) | 1 (0.0%) |
| Micturition frequency decreased | - | - | - | - | - | 1 (0.0%) | 1 (0.0%) |
| Micturition urgency | - | - | - | - | - | 5 (0.0%) | 5 (0.0%) |
| Middle insomnia | - | - | - | - | - | 4 (0.0%) | 4 (0.0%) |
| Mitochondrial encephalomyopathy | - | - | - | - | - | 1 (0.0%) | 1 (0.0%) |
| Mitral valve repair | - | - | - | - | - | 1 (0.0%) | 1 (0.0%) |
| Mixed connective tissue disease | - | - | - | - | - | 1 (0.0%) | 1 (0.0%) |
| Moaning | - | - | - | - | - | 3 (0.0%) | 3 (0.0%) |
| Mobility decreased | - | - | - | - | - | 18 (0.1%) | 18 (0.1%) |
| Modified radical mastectomy | - | - | - | - | - | 1 (0.0%) | 1 (0.0%) |
| Morbid thoughts | - | - | - | - | - | 2 (0.0%) | 2 (0.0%) |
| Motion sickness | - | - | - | - | - | 1 (0.0%) | 1 (0.0%) |
| Mouth ulceration | - | - | - | - | - | 2 (0.0%) | 2 (0.0%) |
| Mucosal dryness | - | - | - | - | - | 1 (0.0%) | 1 (0.0%) |
| Mucosal infection | - | - | - | - | - | 1 (0.0%) | 1 (0.0%) |
| Mucosal inflammation | - | - | - | - | - | 1 (0.0%) | 1 (0.0%) |
| Multiple fractures | - | - | - | - | - | 4 (0.0%) | 4 (0.0%) |
| Multiple injuries | - | - | - | - | - | 1 (0.0%) | 1 (0.0%) |
| Multiple sclerosis relapse | - | - | - | - | - | 3 (0.0%) | 3 (0.0%) |
| Muscle atrophy | - | - | - | - | - | 6 (0.0%) | 6 (0.0%) |
| Muscle disorder | - | - | - | - | - | 1 (0.0%) | 1 (0.0%) |
| Muscle fatigue | - | - | - | - | - | 1 (0.0%) | 1 (0.0%) |
| Muscle injury | - | - | - | - | - | 1 (0.0%) | 1 (0.0%) |
| Muscle necrosis | - | - | - | - | - | 1 (0.0%) | 1 (0.0%) |
| Muscle rupture | - | - | - | - | - | 1 (0.0%) | 1 (0.0%) |
| Myasthenia gravis | - | - | - | - | - | 4 (0.0%) | 4 (0.0%) |
| Mycobacterium avium complex infection | - | - | - | - | - | 1 (0.0%) | 1 (0.0%) |
| Myelosuppression | - | - | - | - | - | 3 (0.0%) | 3 (0.0%) |
| Myocardial depression | - | - | - | - | - | 1 (0.0%) | 1 (0.0%) |
| Myocardial injury | - | - | - | - | - | 1 (0.0%) | 1 (0.0%) |
| Myocardial ischaemia | - | - | - | - | - | 2 (0.0%) | 2 (0.0%) |
| Myocardial necrosis | - | - | - | - | - | 1 (0.0%) | 1 (0.0%) |
| Myocarditis | - | - | - | - | - | 2 (0.0%) | 2 (0.0%) |
| Myofascial pain syndrome | - | - | - | - | - | 1 (0.0%) | 1 (0.0%) |
| Myopathy | - | - | - | - | - | 1 (0.0%) | 1 (0.0%) |
| Myositis | - | - | - | - | - | 1 (0.0%) | 1 (0.0%) |
| Nail growth abnormal | - | - | - | - | - | 2 (0.0%) | 2 (0.0%) |
| Narcolepsy | - | - | - | - | - | 9 (0.0%) | 9 (0.0%) |
| Narcotic bowel syndrome | - | - | - | - | - | 1 (0.0%) | 1 (0.0%) |
| Nasal congestion | - | - | - | - | - | 6 (0.0%) | 6 (0.0%) |
| Nasal cyst | - | - | - | - | - | 1 (0.0%) | 1 (0.0%) |
| Nasal dryness | - | - | - | - | - | 2 (0.0%) | 2 (0.0%) |
| Nasal sinus cancer | - | - | - | - | - | 1 (0.0%) | 1 (0.0%) |
| Near drowning | - | - | - | - | - | 1 (0.0%) | 1 (0.0%) |
| Neck injury | - | - | - | - | - | 2 (0.0%) | 2 (0.0%) |
| Negative thoughts | - | - | - | - | - | 2 (0.0%) | 2 (0.0%) |
| Neoplasm | - | - | - | - | - | 3 (0.0%) | 3 (0.0%) |
| Neoplasm progression | - | - | - | - | - | 3 (0.0%) | 3 (0.0%) |
| Neoplasm recurrence | - | - | - | - | - | 1 (0.0%) | 1 (0.0%) |
| Nephrectomy | - | - | - | - | - | 1 (0.0%) | 1 (0.0%) |
| Nephritis | - | - | - | - | - | 1 (0.0%) | 1 (0.0%) |
| Nephrosclerosis | - | - | - | - | - | 1 (0.0%) | 1 (0.0%) |
| Nerve compression | - | - | - | - | - | 10 (0.0%) | 10 (0.0%) |
| Nerve degeneration | - | - | - | - | - | 1 (0.0%) | 1 (0.0%) |
| Nerve injury | - | - | - | - | - | 15 (0.1%) | 15 (0.0%) |
| Nerve root injury cervical | - | - | - | - | - | 1 (0.0%) | 1 (0.0%) |
| Neuritis | - | - | - | - | - | 1 (0.0%) | 1 (0.0%) |
| Neurogenic bladder | - | - | - | - | - | 1 (0.0%) | 1 (0.0%) |
| Neuroma | - | - | - | - | - | 2 (0.0%) | 2 (0.0%) |
| Neuromyopathy | - | - | - | - | - | 2 (0.0%) | 2 (0.0%) |
| Neurosarcoidosis | - | - | - | - | - | 2 (0.0%) | 2 (0.0%) |
| Neurosis | - | - | - | - | - | 3 (0.0%) | 3 (0.0%) |
| Neutropenia | - | - | - | - | - | 2 (0.0%) | 2 (0.0%) |
| Neutropenic infection | - | - | - | - | - | 1 (0.0%) | 1 (0.0%) |
| Neutrophil count decreased | - | - | - | - | - | 2 (0.0%) | 2 (0.0%) |
| Neutrophilia | - | - | - | - | - | 1 (0.0%) | 1 (0.0%) |
| Nicotine dependence | - | - | - | - | - | 1 (0.0%) | 1 (0.0%) |
| Night sweats | - | - | - | - | - | 12 (0.0%) | 12 (0.0%) |
| Nocturia | - | - | - | - | - | 2 (0.0%) | 2 (0.0%) |
| Nodal arrhythmia | - | - | - | - | - | 1 (0.0%) | 1 (0.0%) |
| Nodule | - | - | - | - | - | 1 (0.0%) | 1 (0.0%) |
| Non-Hodgkin's lymphoma | - | - | - | - | - | 1 (0.0%) | 1 (0.0%) |
| Non-Hodgkin's lymphoma recurrent | - | - | - | - | - | 1 (0.0%) | 1 (0.0%) |
| Non-small cell lung cancer stage IV | - | - | - | - | - | 1 (0.0%) | 1 (0.0%) |
| Nonspecific reaction | - | - | - | - | - | 5 (0.0%) | 5 (0.0%) |
| Normocytic anaemia | - | - | - | - | - | 1 (0.0%) | 1 (0.0%) |
| Nuchal rigidity | - | - | - | - | - | 1 (0.0%) | 1 (0.0%) |
| Obsessive thoughts | - | - | - | - | - | 1 (0.0%) | 1 (0.0%) |
| Occult blood | - | - | - | - | - | 1 (0.0%) | 1 (0.0%) |
| Oedema mouth | - | - | - | - | - | 1 (0.0%) | 1 (0.0%) |
| Oesophageal cancer metastatic | - | - | - | - | - | 1 (0.0%) | 1 (0.0%) |
| Oesophageal candidiasis | - | - | - | - | - | 1 (0.0%) | 1 (0.0%) |
| Oesophageal disorder | - | - | - | - | - | 3 (0.0%) | 3 (0.0%) |
| Oesophageal haemorrhage | - | - | - | - | - | 2 (0.0%) | 2 (0.0%) |
| Oesophageal pain | - | - | - | - | - | 1 (0.0%) | 1 (0.0%) |
| Oesophageal rupture | - | - | - | - | - | 1 (0.0%) | 1 (0.0%) |
| Oesophageal stenosis | - | - | - | - | - | 1 (0.0%) | 1 (0.0%) |
| Oligomenorrhoea | - | - | - | - | - | 1 (0.0%) | 1 (0.0%) |
| Oliguria | - | - | - | - | - | 1 (0.0%) | 1 (0.0%) |
| Onychalgia | - | - | - | - | - | 1 (0.0%) | 1 (0.0%) |
| Onychoclasis | - | - | - | - | - | 5 (0.0%) | 5 (0.0%) |
| Onychomycosis | - | - | - | - | - | 1 (0.0%) | 1 (0.0%) |
| Oophorectomy | - | - | - | - | - | 1 (0.0%) | 1 (0.0%) |
| Ophthalmological examination abnormal | - | - | - | - | - | 1 (0.0%) | 1 (0.0%) |
| Opiates positive | - | - | - | - | - | 1 (0.0%) | 1 (0.0%) |
| Optic nerve injury | - | - | - | - | - | 1 (0.0%) | 1 (0.0%) |
| Oral administration complication | - | - | - | - | - | 1 (0.0%) | 1 (0.0%) |
| Oral discomfort | - | - | - | - | - | 4 (0.0%) | 4 (0.0%) |
| Oral fungal infection | - | - | - | - | - | 1 (0.0%) | 1 (0.0%) |
| Oral herpes | - | - | - | - | - | 1 (0.0%) | 1 (0.0%) |
| Organ failure | - | - | - | - | - | 3 (0.0%) | 3 (0.0%) |
| Oropharyngeal blistering | - | - | - | - | - | 1 (0.0%) | 1 (0.0%) |
| Oropharyngeal cancer | - | - | - | - | - | 1 (0.0%) | 1 (0.0%) |
| Orthopnoea | - | - | - | - | - | 3 (0.0%) | 3 (0.0%) |
| Orthostatic hypertension | - | - | - | - | - | 1 (0.0%) | 1 (0.0%) |
| Osteopenia | - | - | - | - | - | 1 (0.0%) | 1 (0.0%) |
| Osteoradionecrosis | - | - | - | - | - | 1 (0.0%) | 1 (0.0%) |
| Ovarian cancer | - | - | - | - | - | 3 (0.0%) | 3 (0.0%) |
| Ovarian cancer recurrent | - | - | - | - | - | 1 (0.0%) | 1 (0.0%) |
| Ovarian cyst | - | - | - | - | - | 1 (0.0%) | 1 (0.0%) |
| Ovarian cyst ruptured | - | - | - | - | - | 1 (0.0%) | 1 (0.0%) |
| Ovarian neoplasm | - | - | - | - | - | 1 (0.0%) | 1 (0.0%) |
| Oxygen consumption increased | - | - | - | - | - | 1 (0.0%) | 1 (0.0%) |
| Oxygen therapy | - | - | - | - | - | 1 (0.0%) | 1 (0.0%) |
| Pain in jaw | - | - | - | - | - | 2 (0.0%) | 2 (0.0%) |
| Pain management | - | - | - | - | - | 1 (0.0%) | 1 (0.0%) |
| Painful respiration | - | - | - | - | - | 1 (0.0%) | 1 (0.0%) |
| Palmar-plantar erythrodysaesthesia syndrome | - | - | - | - | - | 1 (0.0%) | 1 (0.0%) |
| Pancreas divisum | - | - | - | - | - | 1 (0.0%) | 1 (0.0%) |
| Pancreatic carcinoma metastatic | - | - | - | - | - | 2 (0.0%) | 2 (0.0%) |
| Pancreatic disorder | - | - | - | - | - | 2 (0.0%) | 2 (0.0%) |
| Pancreaticoduodenectomy | - | - | - | - | - | 1 (0.0%) | 1 (0.0%) |
| Pancreatitis acute | - | - | - | - | - | 1 (0.0%) | 1 (0.0%) |
| Pancreatitis chronic | - | - | - | - | - | 1 (0.0%) | 1 (0.0%) |
| Pancytopenia | - | - | - | - | - | 6 (0.0%) | 6 (0.0%) |
| Panic attack | - | - | - | - | - | 29 (0.1%) | 29 (0.1%) |
| Panic disorder | - | - | - | - | - | 2 (0.0%) | 2 (0.0%) |
| Paralysis recurrent laryngeal nerve | - | - | - | - | - | 1 (0.0%) | 1 (0.0%) |
| Paramnesia | - | - | - | - | - | 1 (0.0%) | 1 (0.0%) |
| Parapharyngeal space infection | - | - | - | - | - | 1 (0.0%) | 1 (0.0%) |
| Paratracheal lymphadenopathy | - | - | - | - | - | 1 (0.0%) | 1 (0.0%) |
| Parinaud syndrome | - | - | - | - | - | 1 (0.0%) | 1 (0.0%) |
| Parkinson's disease | - | - | - | - | - | 13 (0.0%) | 13 (0.0%) |
| Parkinsonian crisis | - | - | - | - | - | 1 (0.0%) | 1 (0.0%) |
| Paronychia | - | - | - | - | - | 2 (0.0%) | 2 (0.0%) |
| Patellofemoral pain syndrome | - | - | - | - | - | 1 (0.0%) | 1 (0.0%) |
| Pathological fracture | - | - | - | - | - | 3 (0.0%) | 3 (0.0%) |
| Patient elopement | - | - | - | - | - | 4 (0.0%) | 4 (0.0%) |
| PCO2 decreased | - | - | - | - | - | 1 (0.0%) | 1 (0.0%) |
| Pelvic floor muscle weakness | - | - | - | - | - | 1 (0.0%) | 1 (0.0%) |
| Pelvic fracture | - | - | - | - | - | 5 (0.0%) | 5 (0.0%) |
| Penile ulceration | - | - | - | - | - | 1 (0.0%) | 1 (0.0%) |
| Perforated ulcer | - | - | - | - | - | 1 (0.0%) | 1 (0.0%) |
| Performance status decreased | - | - | - | - | - | 1 (0.0%) | 1 (0.0%) |
| Periarthritis | - | - | - | - | - | 2 (0.0%) | 2 (0.0%) |
| Pericardial effusion malignant | - | - | - | - | - | 1 (0.0%) | 1 (0.0%) |
| Pericarditis | - | - | - | - | - | 5 (0.0%) | 5 (0.0%) |
| Pericarditis adhesive | - | - | - | - | - | 2 (0.0%) | 2 (0.0%) |
| Pericarditis malignant | - | - | - | - | - | 1 (0.0%) | 1 (0.0%) |
| Perineal pain | - | - | - | - | - | 1 (0.0%) | 1 (0.0%) |
| Peripheral artery bypass | - | - | - | - | - | 1 (0.0%) | 1 (0.0%) |
| Peripheral paralysis | - | - | - | - | - | 1 (0.0%) | 1 (0.0%) |
| Peripheral sensorimotor neuropathy | - | - | - | - | - | 1 (0.0%) | 1 (0.0%) |
| Peripheral vascular disorder | - | - | - | - | - | 2 (0.0%) | 2 (0.0%) |
| Perirectal abscess | - | - | - | - | - | 1 (0.0%) | 1 (0.0%) |
| Peritonitis | - | - | - | - | - | 2 (0.0%) | 2 (0.0%) |
| Peroneal nerve palsy | - | - | - | - | - | 1 (0.0%) | 1 (0.0%) |
| Persecutory delusion | - | - | - | - | - | 1 (0.0%) | 1 (0.0%) |
| Persistent depressive disorder | - | - | - | - | - | 2 (0.0%) | 2 (0.0%) |
| Personality change | - | - | - | - | - | 8 (0.0%) | 8 (0.0%) |
| Personality disorder | - | - | - | - | - | 2 (0.0%) | 2 (0.0%) |
| Petechiae | - | - | - | - | - | 2 (0.0%) | 2 (0.0%) |
| Peyronie's disease | - | - | - | - | - | 1 (0.0%) | 1 (0.0%) |
| Phaeochromocytoma | - | - | - | - | - | 1 (0.0%) | 1 (0.0%) |
| Pharyngeal disorder | - | - | - | - | - | 2 (0.0%) | 2 (0.0%) |
| Pharyngeal inflammation | - | - | - | - | - | 1 (0.0%) | 1 (0.0%) |
| Pharyngeal swelling | - | - | - | - | - | 3 (0.0%) | 3 (0.0%) |
| Pharyngitis streptococcal | - | - | - | - | - | 1 (0.0%) | 1 (0.0%) |
| Photopsia | - | - | - | - | - | 2 (0.0%) | 2 (0.0%) |
| Photosensitivity reaction | - | - | - | - | - | 4 (0.0%) | 4 (0.0%) |
| Physical abuse | - | - | - | - | - | 1 (0.0%) | 1 (0.0%) |
| Physical assault | - | - | - | - | - | 2 (0.0%) | 2 (0.0%) |
| Physical deconditioning | - | - | - | - | - | 3 (0.0%) | 3 (0.0%) |
| Physical disability | - | - | - | - | - | 2 (0.0%) | 2 (0.0%) |
| Physical product label issue | - | - | - | - | - | 4 (0.0%) | 4 (0.0%) |
| Pica | - | - | - | - | - | 1 (0.0%) | 1 (0.0%) |
| Pickwickian syndrome | - | - | - | - | - | 2 (0.0%) | 2 (0.0%) |
| Pigmentation disorder | - | - | - | - | - | 1 (0.0%) | 1 (0.0%) |
| Pituitary-dependent Cushing's syndrome | - | - | - | - | - | 1 (0.0%) | 1 (0.0%) |
| Pituitary tumour | - | - | - | - | - | 1 (0.0%) | 1 (0.0%) |
| Pituitary tumour removal | - | - | - | - | - | 1 (0.0%) | 1 (0.0%) |
| Plasma cell myeloma | - | - | - | - | - | 2 (0.0%) | 2 (0.0%) |
| Pneumaturia | - | - | - | - | - | 1 (0.0%) | 1 (0.0%) |
| Pneumococcal sepsis | - | - | - | - | - | 1 (0.0%) | 1 (0.0%) |
| Pneumonia fungal | - | - | - | - | - | 1 (0.0%) | 1 (0.0%) |
| Pneumonia klebsiella | - | - | - | - | - | 1 (0.0%) | 1 (0.0%) |
| Pneumonia pneumococcal | - | - | - | - | - | 1 (0.0%) | 1 (0.0%) |
| Pneumonia viral | - | - | - | - | - | 3 (0.0%) | 3 (0.0%) |
| Pneumothorax spontaneous | - | - | - | - | - | 1 (0.0%) | 1 (0.0%) |
| Pneumothorax traumatic | - | - | - | - | - | 1 (0.0%) | 1 (0.0%) |
| Poisoning deliberate | - | - | - | - | - | 3 (0.0%) | 3 (0.0%) |
| Polydipsia | - | - | - | - | - | 1 (0.0%) | 1 (0.0%) |
| Polymyalgia rheumatica | - | - | - | - | - | 1 (0.0%) | 1 (0.0%) |
| Polyp | - | - | - | - | - | 1 (0.0%) | 1 (0.0%) |
| Polyuria | - | - | - | - | - | 4 (0.0%) | 4 (0.0%) |
| Poor peripheral circulation | - | - | - | - | - | 1 (0.0%) | 1 (0.0%) |
| Poor quality sleep | - | - | - | - | - | 6 (0.0%) | 6 (0.0%) |
| Poriomania | - | - | - | - | - | 1 (0.0%) | 1 (0.0%) |
| Portal vein thrombosis | - | - | - | - | - | 1 (0.0%) | 1 (0.0%) |
| Post herpetic neuralgia | - | - | - | - | - | 1 (0.0%) | 1 (0.0%) |
| Post procedural haemorrhage | - | - | - | - | - | 1 (0.0%) | 1 (0.0%) |
| Post procedural infection | - | - | - | - | - | 2 (0.0%) | 2 (0.0%) |
| Post thrombotic syndrome | - | - | - | - | - | 1 (0.0%) | 1 (0.0%) |
| Postoperative analgesia | - | - | - | - | - | 1 (0.0%) | 1 (0.0%) |
| Postoperative wound infection | - | - | - | - | - | 2 (0.0%) | 2 (0.0%) |
| Posture abnormal | - | - | - | - | - | 4 (0.0%) | 4 (0.0%) |
| Posturing | - | - | - | - | - | 2 (0.0%) | 2 (0.0%) |
| Poverty of speech | - | - | - | - | - | 1 (0.0%) | 1 (0.0%) |
| Precancerous cells present | - | - | - | - | - | 2 (0.0%) | 2 (0.0%) |
| Premature menopause | - | - | - | - | - | 1 (0.0%) | 1 (0.0%) |
| Prescribed underdose | - | - | - | - | - | 1 (0.0%) | 1 (0.0%) |
| Prescription drug used without a prescription | - | - | - | - | - | 5 (0.0%) | 5 (0.0%) |
| Procalcitonin increased | - | - | - | - | - | 1 (0.0%) | 1 (0.0%) |
| Procedural complication | - | - | - | - | - | 1 (0.0%) | 1 (0.0%) |
| Procedural haemorrhage | - | - | - | - | - | 1 (0.0%) | 1 (0.0%) |
| Product administered at inappropriate site | - | - | - | - | - | 37 (0.1%) | 37 (0.1%) |
| Product colour issue | - | - | - | - | - | 2 (0.0%) | 2 (0.0%) |
| Product communication issue | - | - | - | - | - | 1 (0.0%) | 1 (0.0%) |
| Product complaint | - | - | - | - | - | 276 (1.0%) | 276 (0.8%) |
| Product counterfeit | - | - | - | - | - | 1 (0.0%) | 1 (0.0%) |
| Product deposit | - | - | - | - | - | 1 (0.0%) | 1 (0.0%) |
| Product formulation issue | - | - | - | - | - | 1 (0.0%) | 1 (0.0%) |
| Product label issue | - | - | - | - | - | 5 (0.0%) | 5 (0.0%) |
| Product leakage | - | - | - | - | - | 2 (0.0%) | 2 (0.0%) |
| Product odour abnormal | - | - | - | - | - | 2 (0.0%) | 2 (0.0%) |
| Product outer packaging issue | - | - | - | - | - | 4 (0.0%) | 4 (0.0%) |
| Product packaging difficult to open | - | - | - | - | - | 2 (0.0%) | 2 (0.0%) |
| Product packaging issue | - | - | - | - | - | 16 (0.1%) | 16 (0.0%) |
| Product packaging quantity issue | - | - | - | - | - | 19 (0.1%) | 19 (0.1%) |
| Product preparation issue | - | - | - | - | - | 1 (0.0%) | 1 (0.0%) |
| Product prescribing issue | - | - | - | - | - | 2 (0.0%) | 2 (0.0%) |
| Product residue present | - | - | - | - | - | 2 (0.0%) | 2 (0.0%) |
| Product selection error | - | - | - | - | - | 2 (0.0%) | 2 (0.0%) |
| Product size issue | - | - | - | - | - | 2 (0.0%) | 2 (0.0%) |
| Product storage error | - | - | - | - | - | 5 (0.0%) | 5 (0.0%) |
| Product tampering | - | - | - | - | - | 3 (0.0%) | 3 (0.0%) |
| Prostatic specific antigen increased | - | - | - | - | - | 2 (0.0%) | 2 (0.0%) |
| Prostatism | - | - | - | - | - | 1 (0.0%) | 1 (0.0%) |
| Prostatitis | - | - | - | - | - | 1 (0.0%) | 1 (0.0%) |
| Protein total decreased | - | - | - | - | - | 2 (0.0%) | 2 (0.0%) |
| Proteinuria | - | - | - | - | - | 1 (0.0%) | 1 (0.0%) |
| Prurigo | - | - | - | - | - | 1 (0.0%) | 1 (0.0%) |
| Pseudologia | - | - | - | - | - | 1 (0.0%) | 1 (0.0%) |
| Pseudomonas test positive | - | - | - | - | - | 1 (0.0%) | 1 (0.0%) |
| Psoriasis | - | - | - | - | - | 2 (0.0%) | 2 (0.0%) |
| Psychiatric symptom | - | - | - | - | - | 3 (0.0%) | 3 (0.0%) |
| Psychomotor retardation | - | - | - | - | - | 4 (0.0%) | 4 (0.0%) |
| Psychotic behaviour | - | - | - | - | - | 4 (0.0%) | 4 (0.0%) |
| Pulmonary fibrosis | - | - | - | - | - | 1 (0.0%) | 1 (0.0%) |
| Pulmonary mass | - | - | - | - | - | 4 (0.0%) | 4 (0.0%) |
| Pulmonary sepsis | - | - | - | - | - | 1 (0.0%) | 1 (0.0%) |
| Pulmonary thrombosis | - | - | - | - | - | 3 (0.0%) | 3 (0.0%) |
| Pulmonary valve stenosis | - | - | - | - | - | 1 (0.0%) | 1 (0.0%) |
| Pupillary disorder | - | - | - | - | - | 1 (0.0%) | 1 (0.0%) |
| Pupillary light reflex tests abnormal | - | - | - | - | - | 2 (0.0%) | 2 (0.0%) |
| Purulence | - | - | - | - | - | 1 (0.0%) | 1 (0.0%) |
| Pyelonephritis | - | - | - | - | - | 1 (0.0%) | 1 (0.0%) |
| Pyelonephritis acute | - | - | - | - | - | 1 (0.0%) | 1 (0.0%) |
| Pyloric stenosis | - | - | - | - | - | 1 (0.0%) | 1 (0.0%) |
| Radial nerve palsy | - | - | - | - | - | 5 (0.0%) | 5 (0.0%) |
| Radiation skin injury | - | - | - | - | - | 1 (0.0%) | 1 (0.0%) |
| Radius fracture | - | - | - | - | - | 2 (0.0%) | 2 (0.0%) |
| Rapidly progressive osteoarthritis | - | - | - | - | - | 1 (0.0%) | 1 (0.0%) |
| Rash macular | - | - | - | - | - | 3 (0.0%) | 3 (0.0%) |
| Rash pruritic | - | - | - | - | - | 4 (0.0%) | 4 (0.0%) |
| Rash vesicular | - | - | - | - | - | 1 (0.0%) | 1 (0.0%) |
| Raynaud's phenomenon | - | - | - | - | - | 1 (0.0%) | 1 (0.0%) |
| Reaction to excipient | - | - | - | - | - | 1 (0.0%) | 1 (0.0%) |
| Rectal polyp | - | - | - | - | - | 1 (0.0%) | 1 (0.0%) |
| Rectal prolapse | - | - | - | - | - | 1 (0.0%) | 1 (0.0%) |
| Rectourethral fistula | - | - | - | - | - | 1 (0.0%) | 1 (0.0%) |
| Red blood cell count decreased | - | - | - | - | - | 4 (0.0%) | 4 (0.0%) |
| Red blood cell count increased | - | - | - | - | - | 1 (0.0%) | 1 (0.0%) |
| Reduced facial expression | - | - | - | - | - | 1 (0.0%) | 1 (0.0%) |
| Reflexes abnormal | - | - | - | - | - | 4 (0.0%) | 4 (0.0%) |
| Reflux laryngitis | - | - | - | - | - | 1 (0.0%) | 1 (0.0%) |
| Regressive behaviour | - | - | - | - | - | 1 (0.0%) | 1 (0.0%) |
| Regurgitation | - | - | - | - | - | 1 (0.0%) | 1 (0.0%) |
| Rehabilitation therapy | - | - | - | - | - | 1 (0.0%) | 1 (0.0%) |
| Renal cyst | - | - | - | - | - | 3 (0.0%) | 3 (0.0%) |
| Renal mass | - | - | - | - | - | 2 (0.0%) | 2 (0.0%) |
| Renal pain | - | - | - | - | - | 1 (0.0%) | 1 (0.0%) |
| Repetitive speech | - | - | - | - | - | 2 (0.0%) | 2 (0.0%) |
| Respiratory fume inhalation disorder | - | - | - | - | - | 1 (0.0%) | 1 (0.0%) |
| Resuscitation | - | - | - | - | - | 2 (0.0%) | 2 (0.0%) |
| Retinal disorder | - | - | - | - | - | 1 (0.0%) | 1 (0.0%) |
| Retinal tear | - | - | - | - | - | 1 (0.0%) | 1 (0.0%) |
| Reversal of opiate activity | - | - | - | - | - | 1 (0.0%) | 1 (0.0%) |
| Rheumatoid arthritis | - | - | - | - | - | 6 (0.0%) | 6 (0.0%) |
| Rheumatoid nodule | - | - | - | - | - | 1 (0.0%) | 1 (0.0%) |
| Rhinitis | - | - | - | - | - | 2 (0.0%) | 2 (0.0%) |
| Rhinitis allergic | - | - | - | - | - | 1 (0.0%) | 1 (0.0%) |
| Rhonchi | - | - | - | - | - | 2 (0.0%) | 2 (0.0%) |
| Rocky mountain spotted fever | - | - | - | - | - | 1 (0.0%) | 1 (0.0%) |
| Rotator cuff repair | - | - | - | - | - | 1 (0.0%) | 1 (0.0%) |
| Rotator cuff syndrome | - | - | - | - | - | 4 (0.0%) | 4 (0.0%) |
| Ruptured cerebral aneurysm | - | - | - | - | - | 1 (0.0%) | 1 (0.0%) |
| Sacral pain | - | - | - | - | - | 1 (0.0%) | 1 (0.0%) |
| Sacroiliitis | - | - | - | - | - | 1 (0.0%) | 1 (0.0%) |
| Saliva altered | - | - | - | - | - | 1 (0.0%) | 1 (0.0%) |
| Sarcoma | - | - | - | - | - | 1 (0.0%) | 1 (0.0%) |
| Scab | - | - | - | - | - | 2 (0.0%) | 2 (0.0%) |
| Scapula fracture | - | - | - | - | - | 1 (0.0%) | 1 (0.0%) |
| Scar | - | - | - | - | - | 4 (0.0%) | 4 (0.0%) |
| Schizophrenia | - | - | - | - | - | 4 (0.0%) | 4 (0.0%) |
| Sciatic nerve injury | - | - | - | - | - | 1 (0.0%) | 1 (0.0%) |
| Scintillating scotoma | - | - | - | - | - | 1 (0.0%) | 1 (0.0%) |
| Scoliosis | - | - | - | - | - | 3 (0.0%) | 3 (0.0%) |
| Scratch | - | - | - | - | - | 1 (0.0%) | 1 (0.0%) |
| Seborrhoea | - | - | - | - | - | 1 (0.0%) | 1 (0.0%) |
| Seborrhoeic dermatitis | - | - | - | - | - | 1 (0.0%) | 1 (0.0%) |
| Secondary hypogonadism | - | - | - | - | - | 1 (0.0%) | 1 (0.0%) |
| Secretion discharge | - | - | - | - | - | 2 (0.0%) | 2 (0.0%) |
| Self-injurious ideation | - | - | - | - | - | 2 (0.0%) | 2 (0.0%) |
| Self-medication | - | - | - | - | - | 3 (0.0%) | 3 (0.0%) |
| Septic shock | - | - | - | - | - | 12 (0.0%) | 12 (0.0%) |
| Serum ferritin decreased | - | - | - | - | - | 1 (0.0%) | 1 (0.0%) |
| Sexual dysfunction | - | - | - | - | - | 3 (0.0%) | 3 (0.0%) |
| Shoulder arthroplasty | - | - | - | - | - | 1 (0.0%) | 1 (0.0%) |
| Shoulder fracture | - | - | - | - | - | 8 (0.0%) | 8 (0.0%) |
| Shoulder operation | - | - | - | - | - | 2 (0.0%) | 2 (0.0%) |
| Shunt infection | - | - | - | - | - | 1 (0.0%) | 1 (0.0%) |
| Shunt stenosis | - | - | - | - | - | 1 (0.0%) | 1 (0.0%) |
| Sinus disorder | - | - | - | - | - | 3 (0.0%) | 3 (0.0%) |
| Sinus node dysfunction | - | - | - | - | - | 1 (0.0%) | 1 (0.0%) |
| Sinus pain | - | - | - | - | - | 1 (0.0%) | 1 (0.0%) |
| Sjogren's syndrome | - | - | - | - | - | 2 (0.0%) | 2 (0.0%) |
| Skeletal injury | - | - | - | - | - | 2 (0.0%) | 2 (0.0%) |
| Skin abrasion | - | - | - | - | - | 3 (0.0%) | 3 (0.0%) |
| Skin atrophy | - | - | - | - | - | 1 (0.0%) | 1 (0.0%) |
| Skin burning sensation | - | - | - | - | - | 10 (0.0%) | 10 (0.0%) |
| Skin cancer | - | - | - | - | - | 1 (0.0%) | 1 (0.0%) |
| Skin candida | - | - | - | - | - | 1 (0.0%) | 1 (0.0%) |
| Skin disorder | - | - | - | - | - | 4 (0.0%) | 4 (0.0%) |
| Skin erosion | - | - | - | - | - | 1 (0.0%) | 1 (0.0%) |
| Skin fissures | - | - | - | - | - | 1 (0.0%) | 1 (0.0%) |
| Skin infection | - | - | - | - | - | 1 (0.0%) | 1 (0.0%) |
| Skin injury | - | - | - | - | - | 1 (0.0%) | 1 (0.0%) |
| Skin irritation | - | - | - | - | - | 10 (0.0%) | 10 (0.0%) |
| Skin laceration | - | - | - | - | - | 8 (0.0%) | 8 (0.0%) |
| Skin necrosis | - | - | - | - | - | 1 (0.0%) | 1 (0.0%) |
| Skin odour abnormal | - | - | - | - | - | 4 (0.0%) | 4 (0.0%) |
| Skin reaction | - | - | - | - | - | 3 (0.0%) | 3 (0.0%) |
| Skin test negative | - | - | - | - | - | 1 (0.0%) | 1 (0.0%) |
| Skin tightness | - | - | - | - | - | 1 (0.0%) | 1 (0.0%) |
| Skin ulcer | - | - | - | - | - | 4 (0.0%) | 4 (0.0%) |
| Skin ulcer haemorrhage | - | - | - | - | - | 1 (0.0%) | 1 (0.0%) |
| Skin warm | - | - | - | - | - | 5 (0.0%) | 5 (0.0%) |
| Skull fracture | - | - | - | - | - | 1 (0.0%) | 1 (0.0%) |
| Sleep deficit | - | - | - | - | - | 3 (0.0%) | 3 (0.0%) |
| Sleep talking | - | - | - | - | - | 1 (0.0%) | 1 (0.0%) |
| Sleep terror | - | - | - | - | - | 3 (0.0%) | 3 (0.0%) |
| Slow speech | - | - | - | - | - | 3 (0.0%) | 3 (0.0%) |
| Sluggishness | - | - | - | - | - | 7 (0.0%) | 7 (0.0%) |
| Small cell lung cancer extensive stage | - | - | - | - | - | 1 (0.0%) | 1 (0.0%) |
| Small intestinal obstruction | - | - | - | - | - | 2 (0.0%) | 2 (0.0%) |
| Small intestinal resection | - | - | - | - | - | 1 (0.0%) | 1 (0.0%) |
| Small intestine carcinoma | - | - | - | - | - | 1 (0.0%) | 1 (0.0%) |
| Sneezing | - | - | - | - | - | 11 (0.0%) | 11 (0.0%) |
| Social problem | - | - | - | - | - | 1 (0.0%) | 1 (0.0%) |
| Soft tissue injury | - | - | - | - | - | 1 (0.0%) | 1 (0.0%) |
| Somatic symptom disorder | - | - | - | - | - | 1 (0.0%) | 1 (0.0%) |
| Spinal compression fracture | - | - | - | - | - | 6 (0.0%) | 6 (0.0%) |
| Spinal cord abscess | - | - | - | - | - | 1 (0.0%) | 1 (0.0%) |
| Spinal cord oedema | - | - | - | - | - | 1 (0.0%) | 1 (0.0%) |
| Spinal cord operation | - | - | - | - | - | 2 (0.0%) | 2 (0.0%) |
| Spinal cord paralysis | - | - | - | - | - | 1 (0.0%) | 1 (0.0%) |
| Spinal disorder | - | - | - | - | - | 11 (0.0%) | 11 (0.0%) |
| Spinal fusion surgery | - | - | - | - | - | 6 (0.0%) | 6 (0.0%) |
| Spinal laminectomy | - | - | - | - | - | 1 (0.0%) | 1 (0.0%) |
| Spinal operation | - | - | - | - | - | 10 (0.0%) | 10 (0.0%) |
| Spinal osteoarthritis | - | - | - | - | - | 5 (0.0%) | 5 (0.0%) |
| Spinal stenosis | - | - | - | - | - | 6 (0.0%) | 6 (0.0%) |
| Splenic lesion | - | - | - | - | - | 1 (0.0%) | 1 (0.0%) |
| Splenomegaly | - | - | - | - | - | 1 (0.0%) | 1 (0.0%) |
| Spondylitis | - | - | - | - | - | 3 (0.0%) | 3 (0.0%) |
| Squamous cell carcinoma | - | - | - | - | - | 1 (0.0%) | 1 (0.0%) |
| Staphylococcal sepsis | - | - | - | - | - | 5 (0.0%) | 5 (0.0%) |
| Staphylococcus test | - | - | - | - | - | 1 (0.0%) | 1 (0.0%) |
| Staring | - | - | - | - | - | 2 (0.0%) | 2 (0.0%) |
| Stenosis | - | - | - | - | - | 1 (0.0%) | 1 (0.0%) |
| Sternal fracture | - | - | - | - | - | 1 (0.0%) | 1 (0.0%) |
| Steroid diabetes | - | - | - | - | - | 2 (0.0%) | 2 (0.0%) |
| Stevens-Johnson syndrome | - | - | - | - | - | 1 (0.0%) | 1 (0.0%) |
| Streptococcal infection | - | - | - | - | - | 1 (0.0%) | 1 (0.0%) |
| Stress fracture | - | - | - | - | - | 1 (0.0%) | 1 (0.0%) |
| Stress urinary incontinence | - | - | - | - | - | 1 (0.0%) | 1 (0.0%) |
| Stridor | - | - | - | - | - | 2 (0.0%) | 2 (0.0%) |
| Subdural haematoma | - | - | - | - | - | 5 (0.0%) | 5 (0.0%) |
| Subileus | - | - | - | - | - | 3 (0.0%) | 3 (0.0%) |
| Substance-induced psychotic disorder | - | - | - | - | - | 1 (0.0%) | 1 (0.0%) |
| Substance use | - | - | - | - | - | 2 (0.0%) | 2 (0.0%) |
| Sudden death | - | - | - | - | - | 2 (0.0%) | 2 (0.0%) |
| Suicidal behaviour | - | - | - | - | - | 1 (0.0%) | 1 (0.0%) |
| Sunburn | - | - | - | - | - | 4 (0.0%) | 4 (0.0%) |
| Superficial vein thrombosis | - | - | - | - | - | 1 (0.0%) | 1 (0.0%) |
| Supranuclear palsy | - | - | - | - | - | 1 (0.0%) | 1 (0.0%) |
| Suspected product quality issue | - | - | - | - | - | 1 (0.0%) | 1 (0.0%) |
| Suture rupture | - | - | - | - | - | 1 (0.0%) | 1 (0.0%) |
| Swollen tongue | - | - | - | - | - | 6 (0.0%) | 6 (0.0%) |
| Synovitis | - | - | - | - | - | 1 (0.0%) | 1 (0.0%) |
| Systemic infection | - | - | - | - | - | 1 (0.0%) | 1 (0.0%) |
| Systemic lupus erythematosus | - | - | - | - | - | 4 (0.0%) | 4 (0.0%) |
| Tachyphrenia | - | - | - | - | - | 2 (0.0%) | 2 (0.0%) |
| Tachyphylaxis | - | - | - | - | - | 1 (0.0%) | 1 (0.0%) |
| Tanning | - | - | - | - | - | 1 (0.0%) | 1 (0.0%) |
| Temperature regulation disorder | - | - | - | - | - | 3 (0.0%) | 3 (0.0%) |
| Tendon rupture | - | - | - | - | - | 2 (0.0%) | 2 (0.0%) |
| Tension | - | - | - | - | - | 3 (0.0%) | 3 (0.0%) |
| Tension headache | - | - | - | - | - | 1 (0.0%) | 1 (0.0%) |
| Terminal state | - | - | - | - | - | 2 (0.0%) | 2 (0.0%) |
| Testicular pain | - | - | - | - | - | 1 (0.0%) | 1 (0.0%) |
| Tetany | - | - | - | - | - | 1 (0.0%) | 1 (0.0%) |
| Therapeutic agent urine positive | - | - | - | - | - | 1 (0.0%) | 1 (0.0%) |
| Therapeutic hypothermia | - | - | - | - | - | 1 (0.0%) | 1 (0.0%) |
| Therapeutic product effect delayed | - | - | - | - | - | 1 (0.0%) | 1 (0.0%) |
| Therapeutic product ineffective | - | - | - | - | - | 1 (0.0%) | 1 (0.0%) |
| Therapeutic reaction time decreased | - | - | - | - | - | 1 (0.0%) | 1 (0.0%) |
| Therapeutic response changed | - | - | - | - | - | 3 (0.0%) | 3 (0.0%) |
| Therapeutic response delayed | - | - | - | - | - | 3 (0.0%) | 3 (0.0%) |
| Therapeutic response increased | - | - | - | - | - | 6 (0.0%) | 6 (0.0%) |
| Therapeutic response shortened | - | - | - | - | - | 3 (0.0%) | 3 (0.0%) |
| Therapy cessation | - | - | - | - | - | 4 (0.0%) | 4 (0.0%) |
| Therapy change | - | - | - | - | - | 2 (0.0%) | 2 (0.0%) |
| Therapy naive | - | - | - | - | - | 5 (0.0%) | 5 (0.0%) |
| Thermohypoaesthesia | - | - | - | - | - | 1 (0.0%) | 1 (0.0%) |
| Thirst decreased | - | - | - | - | - | 4 (0.0%) | 4 (0.0%) |
| Thoracic outlet syndrome | - | - | - | - | - | 1 (0.0%) | 1 (0.0%) |
| Thoracic spinal cord paralysis | - | - | - | - | - | 1 (0.0%) | 1 (0.0%) |
| Thoracic vertebral fracture | - | - | - | - | - | 1 (0.0%) | 1 (0.0%) |
| Thought blocking | - | - | - | - | - | 1 (0.0%) | 1 (0.0%) |
| Throat cancer | - | - | - | - | - | 1 (0.0%) | 1 (0.0%) |
| Throat irritation | - | - | - | - | - | 4 (0.0%) | 4 (0.0%) |
| Throat tightness | - | - | - | - | - | 10 (0.0%) | 10 (0.0%) |
| Thrombocytopenia | - | - | - | - | - | 6 (0.0%) | 6 (0.0%) |
| Thrombosis in device | - | - | - | - | - | 1 (0.0%) | 1 (0.0%) |
| Thyroid cancer | - | - | - | - | - | 1 (0.0%) | 1 (0.0%) |
| Thyroid disorder | - | - | - | - | - | 9 (0.0%) | 9 (0.0%) |
| Thyroid mass | - | - | - | - | - | 2 (0.0%) | 2 (0.0%) |
| Thyroid neoplasm | - | - | - | - | - | 1 (0.0%) | 1 (0.0%) |
| Thyrotoxic crisis | - | - | - | - | - | 1 (0.0%) | 1 (0.0%) |
| Tibia fracture | - | - | - | - | - | 1 (0.0%) | 1 (0.0%) |
| Tobacco abuse | - | - | - | - | - | 1 (0.0%) | 1 (0.0%) |
| Tobacco user | - | - | - | - | - | 3 (0.0%) | 3 (0.0%) |
| Tongue biting | - | - | - | - | - | 1 (0.0%) | 1 (0.0%) |
| Tongue blistering | - | - | - | - | - | 1 (0.0%) | 1 (0.0%) |
| Tongue discolouration | - | - | - | - | - | 3 (0.0%) | 3 (0.0%) |
| Tongue discomfort | - | - | - | - | - | 2 (0.0%) | 2 (0.0%) |
| Tongue dry | - | - | - | - | - | 1 (0.0%) | 1 (0.0%) |
| Tongue exfoliation | - | - | - | - | - | 1 (0.0%) | 1 (0.0%) |
| Tongue oedema | - | - | - | - | - | 1 (0.0%) | 1 (0.0%) |
| Tongue operation | - | - | - | - | - | 1 (0.0%) | 1 (0.0%) |
| Tongue paralysis | - | - | - | - | - | 1 (0.0%) | 1 (0.0%) |
| Tooth discolouration | - | - | - | - | - | 1 (0.0%) | 1 (0.0%) |
| Torticollis | - | - | - | - | - | 1 (0.0%) | 1 (0.0%) |
| Tracheal stenosis | - | - | - | - | - | 1 (0.0%) | 1 (0.0%) |
| Transfusion reaction | - | - | - | - | - | 1 (0.0%) | 1 (0.0%) |
| Trichotillomania | - | - | - | - | - | 1 (0.0%) | 1 (0.0%) |
| Trigeminal neuralgia | - | - | - | - | - | 2 (0.0%) | 2 (0.0%) |
| Trigger finger | - | - | - | - | - | 3 (0.0%) | 3 (0.0%) |
| Tumour associated fever | - | - | - | - | - | 1 (0.0%) | 1 (0.0%) |
| Tumour haemorrhage | - | - | - | - | - | 1 (0.0%) | 1 (0.0%) |
| Tunnel vision | - | - | - | - | - | 1 (0.0%) | 1 (0.0%) |
| Type 1 diabetes mellitus | - | - | - | - | - | 1 (0.0%) | 1 (0.0%) |
| Type 2 diabetes mellitus | - | - | - | - | - | 3 (0.0%) | 3 (0.0%) |
| Ulcer haemorrhage | - | - | - | - | - | 2 (0.0%) | 2 (0.0%) |
| Ulnar nerve palsy | - | - | - | - | - | 1 (0.0%) | 1 (0.0%) |
| Underdose | - | - | - | - | - | 21 (0.1%) | 21 (0.1%) |
| Upper gastrointestinal haemorrhage | - | - | - | - | - | 2 (0.0%) | 2 (0.0%) |
| Upper limb fracture | - | - | - | - | - | 3 (0.0%) | 3 (0.0%) |
| Upper respiratory tract infection | - | - | - | - | - | 9 (0.0%) | 9 (0.0%) |
| Upper respiratory tract inflammation | - | - | - | - | - | 1 (0.0%) | 1 (0.0%) |
| Ureteric cancer | - | - | - | - | - | 1 (0.0%) | 1 (0.0%) |
| Urinary bladder polyp | - | - | - | - | - | 1 (0.0%) | 1 (0.0%) |
| Urinary hesitation | - | - | - | - | - | 3 (0.0%) | 3 (0.0%) |
| Urinary tract candidiasis | - | - | - | - | - | 1 (0.0%) | 1 (0.0%) |
| Urine abnormality | - | - | - | - | - | 1 (0.0%) | 1 (0.0%) |
| Urine odour abnormal | - | - | - | - | - | 2 (0.0%) | 2 (0.0%) |
| Urogenital infection bacterial | - | - | - | - | - | 1 (0.0%) | 1 (0.0%) |
| Urosepsis | - | - | - | - | - | 3 (0.0%) | 3 (0.0%) |
| Uterine cancer | - | - | - | - | - | 3 (0.0%) | 3 (0.0%) |
| Uveitis | - | - | - | - | - | 1 (0.0%) | 1 (0.0%) |
| Vaginoplasty | - | - | - | - | - | 1 (0.0%) | 1 (0.0%) |
| Varicella | - | - | - | - | - | 1 (0.0%) | 1 (0.0%) |
| Varicose vein | - | - | - | - | - | 2 (0.0%) | 2 (0.0%) |
| Vascular dementia | - | - | - | - | - | 2 (0.0%) | 2 (0.0%) |
| Vascular occlusion | - | - | - | - | - | 1 (0.0%) | 1 (0.0%) |
| Vasculitis | - | - | - | - | - | 3 (0.0%) | 3 (0.0%) |
| Vasoconstriction | - | - | - | - | - | 1 (0.0%) | 1 (0.0%) |
| Vein disorder | - | - | - | - | - | 2 (0.0%) | 2 (0.0%) |
| Ventricular hypokinesia | - | - | - | - | - | 1 (0.0%) | 1 (0.0%) |
| Verbal abuse | - | - | - | - | - | 1 (0.0%) | 1 (0.0%) |
| Victim of sexual abuse | - | - | - | - | - | 1 (0.0%) | 1 (0.0%) |
| Victim of spousal abuse | - | - | - | - | - | 1 (0.0%) | 1 (0.0%) |
| Viral pericarditis | - | - | - | - | - | 1 (0.0%) | 1 (0.0%) |
| Visceral congestion | - | - | - | - | - | 1 (0.0%) | 1 (0.0%) |
| Vitamin D decreased | - | - | - | - | - | 2 (0.0%) | 2 (0.0%) |
| Vitamin D deficiency | - | - | - | - | - | 3 (0.0%) | 3 (0.0%) |
| Vocal cord disorder | - | - | - | - | - | 1 (0.0%) | 1 (0.0%) |
| Volvulus | - | - | - | - | - | 1 (0.0%) | 1 (0.0%) |
| Vomiting projectile | - | - | - | - | - | 4 (0.0%) | 4 (0.0%) |
| Walking aid user | - | - | - | - | - | 2 (0.0%) | 2 (0.0%) |
| Walking disability | - | - | - | - | - | 1 (0.0%) | 1 (0.0%) |
| Weight fluctuation | - | - | - | - | - | 4 (0.0%) | 4 (0.0%) |
| Wheelchair user | - | - | - | - | - | 1 (0.0%) | 1 (0.0%) |
| Wheezing | - | - | - | - | - | 10 (0.0%) | 10 (0.0%) |
| White blood cell disorder | - | - | - | - | - | 1 (0.0%) | 1 (0.0%) |
| Wolff-Parkinson-White syndrome | - | - | - | - | - | 1 (0.0%) | 1 (0.0%) |
| Wound infection | - | - | - | - | - | 1 (0.0%) | 1 (0.0%) |
| Wrist fracture | - | - | - | - | - | 10 (0.0%) | 10 (0.0%) |
| Wrong schedule | - | - | - | - | - | 2 (0.0%) | 2 (0.0%) |
| Wrong strength | - | - | - | - | - | 1 (0.0%) | 1 (0.0%) |
| Yellow skin | - | - | - | - | - | 1 (0.0%) | 1 (0.0%) |

**Supplementary Table S2** Distribution of preferred terms (PT) categorized under the System Organ Class (SOC) 'Nervous system disorders' retrivered from Individual Case Safety Reports (ICSRs) where Fentanyl is identified as the suspected drug, in the EudraVigilance database, up to March 7, 2024.

|  | **Oral transmucosal (N=357)** | **Intramuscular (N=8)** | **Intravenous (N=1209)** | **Nasal (N=107)** | **Other (N=140)** | **Transdermal (N=7618)** | **Overall (N=9439)** |
| --- | --- | --- | --- | --- | --- | --- | --- |
| **Preferred terms** |  |  |  |  |  |  |  |
| Ageusia | 4 (1.1%) | - | 2 (0.2%) | - | - | 4 (0.1%) | 10 (0.1%) |
| Akathisia | 1 (0.3%) | - | 5 (0.4%) | - | - | 23 (0.3%) | 29 (0.3%) |
| Allodynia | 3 (0.8%) | - | 2 (0.2%) | - | 1 (0.7%) | 9 (0.1%) | 15 (0.2%) |
| Altered state of consciousness | 15 (4.2%) | - | 22 (1.8%) | 1 (0.9%) | 3 (2.1%) | 120 (1.6%) | 161 (1.7%) |
| Amnesia | 7 (2.0%) | - | 5 (0.4%) | 1 (0.9%) | 1 (0.7%) | 140 (1.8%) | 154 (1.6%) |
| Ataxia | 1 (0.3%) | - | 7 (0.6%) | - | - | 20 (0.3%) | 28 (0.3%) |
| Balance disorder | 2 (0.6%) | - | - | 1 (0.9%) | - | 69 (0.9%) | 72 (0.8%) |
| Bradykinesia | 1 (0.3%) | - | 2 (0.2%) | - | - | 8 (0.1%) | 11 (0.1%) |
| Brain injury | 1 (0.3%) | - | 8 (0.7%) | 1 (0.9%) | 1 (0.7%) | 36 (0.5%) | 47 (0.5%) |
| Brain oedema | 1 (0.3%) | - | 6 (0.5%) | 1 (0.9%) | - | 30 (0.4%) | 38 (0.4%) |
| Burning sensation | 1 (0.3%) | - | 3 (0.2%) | 1 (0.9%) | - | 43 (0.6%) | 48 (0.5%) |
| Burning sensation mucosal | 1 (0.3%) | - | - | - | - | - | 1 (0.0%) |
| Central nervous system lesion | 1 (0.3%) | - | - | - | - | 2 (0.0%) | 3 (0.0%) |
| Cerebrovascular accident | 3 (0.8%) | - | 2 (0.2%) | 3 (2.8%) | 2 (1.4%) | 120 (1.6%) | 130 (1.4%) |
| Coma | 8 (2.2%) | 2 (25.0%) | 39 (3.2%) | 13 (12.1%) | 9 (6.4%) | 298 (3.9%) | 369 (3.9%) |
| Depressed level of consciousness | 17 (4.8%) | 1 (12.5%) | 63 (5.2%) | 3 (2.8%) | 9 (6.4%) | 308 (4.0%) | 401 (4.2%) |
| Disturbance in attention | 4 (1.1%) | - | 7 (0.6%) | 2 (1.9%) | 3 (2.1%) | 99 (1.3%) | 115 (1.2%) |
| Dizziness | 20 (5.6%) | - | 25 (2.1%) | 4 (3.7%) | 4 (2.9%) | 660 (8.7%) | 713 (7.6%) |
| Drooling | 1 (0.3%) | - | - | - | 1 (0.7%) | 5 (0.1%) | 7 (0.1%) |
| Drug withdrawal convulsions | 1 (0.3%) | - | 1 (0.1%) | - | - | 13 (0.2%) | 15 (0.2%) |
| Dysaesthesia | 1 (0.3%) | - | - | - | - | 5 (0.1%) | 6 (0.1%) |
| Dysarthria | 3 (0.8%) | - | 4 (0.3%) | 2 (1.9%) | 1 (0.7%) | 94 (1.2%) | 104 (1.1%) |
| Dysgeusia | 2 (0.6%) | - | 1 (0.1%) | - | - | 17 (0.2%) | 20 (0.2%) |
| Dystonia | 1 (0.3%) | 1 (12.5%) | 16 (1.3%) | 1 (0.9%) | 1 (0.7%) | 3 (0.0%) | 23 (0.2%) |
| Encephalopathy | 2 (0.6%) | - | 9 (0.7%) | - | 2 (1.4%) | 32 (0.4%) | 45 (0.5%) |
| Facial paralysis | 1 (0.3%) | - | 2 (0.2%) | 1 (0.9%) | - | 6 (0.1%) | 10 (0.1%) |
| Formication | 2 (0.6%) | - | - | - | - | 40 (0.5%) | 42 (0.4%) |
| Generalised tonic-clonic seizure | 2 (0.6%) | - | 25 (2.1%) | - | - | 28 (0.4%) | 55 (0.6%) |
| Headache | 9 (2.5%) | - | 38 (3.1%) | 5 (4.7%) | 1 (0.7%) | 341 (4.5%) | 394 (4.2%) |
| Hepatic encephalopathy | 2 (0.6%) | - | 2 (0.2%) | - | - | 11 (0.1%) | 15 (0.2%) |
| Hyperaesthesia | 7 (2.0%) | - | 21 (1.7%) | 2 (1.9%) | 5 (3.6%) | 63 (0.8%) | 98 (1.0%) |
| Hyperresponsive to stimuli | 1 (0.3%) | - | - | - | - | - | 1 (0.0%) |
| Hypertonia | 1 (0.3%) | - | 9 (0.7%) | - | - | 2 (0.0%) | 12 (0.1%) |
| Hypoaesthesia | 3 (0.8%) | - | 9 (0.7%) | 2 (1.9%) | 1 (0.7%) | 96 (1.3%) | 111 (1.2%) |
| Hypokinesia | 1 (0.3%) | - | 2 (0.2%) | - | - | 31 (0.4%) | 34 (0.4%) |
| Hyporeflexia | 1 (0.3%) | - | - | - | - | 3 (0.0%) | 4 (0.0%) |
| Hypotonia | 1 (0.3%) | - | 6 (0.5%) | - | - | 12 (0.2%) | 19 (0.2%) |
| Incoherent | 4 (1.1%) | - | - | - | - | 50 (0.7%) | 54 (0.6%) |
| Intracranial aneurysm | 1 (0.3%) | - | - | - | - | 9 (0.1%) | 10 (0.1%) |
| Lethargy | 4 (1.1%) | - | 4 (0.3%) | 2 (1.9%) | 2 (1.4%) | 141 (1.9%) | 153 (1.6%) |
| Loss of consciousness | 20 (5.6%) | 1 (12.5%) | 65 (5.4%) | 8 (7.5%) | 3 (2.1%) | 415 (5.4%) | 512 (5.4%) |
| Memory impairment | 5 (1.4%) | - | 6 (0.5%) | 1 (0.9%) | 2 (1.4%) | 132 (1.7%) | 146 (1.5%) |
| Mental impairment | 4 (1.1%) | - | 2 (0.2%) | - | 1 (0.7%) | 65 (0.9%) | 72 (0.8%) |
| Metabolic encephalopathy | 1 (0.3%) | - | - | - | - | 6 (0.1%) | 7 (0.1%) |
| Migraine | 8 (2.2%) | - | - | 1 (0.9%) | - | 84 (1.1%) | 93 (1.0%) |
| Monoplegia | 2 (0.6%) | - | 2 (0.2%) | - | 1 (0.7%) | 8 (0.1%) | 13 (0.1%) |
| Motor dysfunction | 3 (0.8%) | - | 2 (0.2%) | - | - | 5 (0.1%) | 10 (0.1%) |
| Multiple sclerosis | 2 (0.6%) | - | 1 (0.1%) | - | - | 19 (0.2%) | 22 (0.2%) |
| Myelopathy | 1 (0.3%) | - | - | - | - | 3 (0.0%) | 4 (0.0%) |
| Myoclonus | 2 (0.6%) | - | 36 (3.0%) | 1 (0.9%) | 2 (1.4%) | 67 (0.9%) | 108 (1.1%) |
| Nervous system disorder | 1 (0.3%) | - | 6 (0.5%) | - | 1 (0.7%) | 14 (0.2%) | 22 (0.2%) |
| Neurotoxicity | 2 (0.6%) | - | 7 (0.6%) | - | 2 (1.4%) | 16 (0.2%) | 27 (0.3%) |
| Pachymeningitis | 1 (0.3%) | - | - | - | - | - | 1 (0.0%) |
| Paraesthesia | 6 (1.7%) | - | 7 (0.6%) | 1 (0.9%) | - | 102 (1.3%) | 116 (1.2%) |
| Paralysis | 3 (0.8%) | - | 5 (0.4%) | 1 (0.9%) | - | 17 (0.2%) | 26 (0.3%) |
| Paraplegia | 1 (0.3%) | - | 4 (0.3%) | - | - | 3 (0.0%) | 8 (0.1%) |
| Peripheral sensory neuropathy | 1 (0.3%) | - | - | - | - | 2 (0.0%) | 3 (0.0%) |
| Phantom limb syndrome | 1 (0.3%) | - | 1 (0.1%) | - | - | 4 (0.1%) | 6 (0.1%) |
| Polyneuropathy | 1 (0.3%) | - | - | - | - | - | 1 (0.0%) |
| Polyneuropathy in malignant disease | 1 (0.3%) | - | - | - | - | - | 1 (0.0%) |
| Presyncope | 3 (0.8%) | - | 9 (0.7%) | - | - | 22 (0.3%) | 34 (0.4%) |
| Psychomotor hyperactivity | 1 (0.3%) | - | 6 (0.5%) | - | - | 42 (0.6%) | 49 (0.5%) |
| Psychomotor skills impaired | 2 (0.6%) | - | 1 (0.1%) | - | - | 10 (0.1%) | 13 (0.1%) |
| Quadriparesis | 1 (0.3%) | - | 1 (0.1%) | - | - | 2 (0.0%) | 4 (0.0%) |
| Restless legs syndrome | 1 (0.3%) | - | - | - | 1 (0.7%) | 38 (0.5%) | 40 (0.4%) |
| Sciatica | 1 (0.3%) | - | - | - | - | 17 (0.2%) | 18 (0.2%) |
| Sedation | 2 (0.6%) | - | 19 (1.6%) | 2 (1.9%) | 6 (4.3%) | 142 (1.9%) | 171 (1.8%) |
| Seizure | 18 (5.0%) | 1 (12.5%) | 64 (5.3%) | 3 (2.8%) | 8 (5.7%) | 304 (4.0%) | 398 (4.2%) |
| Serotonin syndrome | 4 (1.1%) | - | 89 (7.4%) | 1 (0.9%) | 1 (0.7%) | 66 (0.9%) | 161 (1.7%) |
| Somnolence | 86 (24.1%) | 1 (12.5%) | 67 (5.5%) | 14 (13.1%) | 40 (28.6%) | 1163 (15.3%) | 1371 (14.5%) |
| Speech disorder | 2 (0.6%) | - | 2 (0.2%) | - | 2 (1.4%) | 76 (1.0%) | 82 (0.9%) |
| Stupor | 1 (0.3%) | - | 7 (0.6%) | - | - | 42 (0.6%) | 50 (0.5%) |
| Syncope | 2 (0.6%) | - | 6 (0.5%) | 1 (0.9%) | 1 (0.7%) | 117 (1.5%) | 127 (1.3%) |
| Taste disorder | 3 (0.8%) | - | - | 1 (0.9%) | 1 (0.7%) | 23 (0.3%) | 28 (0.3%) |
| Transient ischaemic attack | 1 (0.3%) | - | 1 (0.1%) | 1 (0.9%) | - | 24 (0.3%) | 27 (0.3%) |
| Tremor | 15 (4.2%) | 1 (12.5%) | 40 (3.3%) | 2 (1.9%) | 3 (2.1%) | 363 (4.8%) | 424 (4.5%) |
| Unresponsive to stimuli | 6 (1.7%) | - | 59 (4.9%) | 10 (9.3%) | 3 (2.1%) | 146 (1.9%) | 224 (2.4%) |
| Agitation neonatal | - | - | 1 (0.1%) | - | - | - | 1 (0.0%) |
| Akinesia | - | - | 3 (0.2%) | - | - | - | 3 (0.0%) |
| Amimia | - | - | 1 (0.1%) | - | - | 1 (0.0%) | 2 (0.0%) |
| Amnestic disorder | - | - | 1 (0.1%) | 1 (0.9%) | - | 3 (0.0%) | 5 (0.1%) |
| Anosmia | - | - | 4 (0.3%) | 1 (0.9%) | - | 1 (0.0%) | 6 (0.1%) |
| Anterograde amnesia | - | - | 4 (0.3%) | - | - | - | 4 (0.0%) |
| Anticholinergic syndrome | - | - | 5 (0.4%) | - | - | - | 5 (0.1%) |
| Apallic syndrome | - | - | 2 (0.2%) | - | - | 1 (0.0%) | 3 (0.0%) |
| Aphasia | - | - | 6 (0.5%) | - | - | 43 (0.6%) | 49 (0.5%) |
| Arachnoiditis | - | - | 1 (0.1%) | - | - | 6 (0.1%) | 7 (0.1%) |
| Areflexia | - | - | 8 (0.7%) | - | - | 3 (0.0%) | 11 (0.1%) |
| Asterixis | - | - | 1 (0.1%) | - | - | 3 (0.0%) | 4 (0.0%) |
| Autonomic nervous system imbalance | - | - | 4 (0.3%) | - | - | 3 (0.0%) | 7 (0.1%) |
| Basal ganglion degeneration | - | - | 1 (0.1%) | - | - | - | 1 (0.0%) |
| Brain hypoxia | - | - | 2 (0.2%) | - | - | 4 (0.1%) | 6 (0.1%) |
| Cerebellar haemorrhage | - | - | 2 (0.2%) | - | - | - | 2 (0.0%) |
| Cerebellar infarction | - | - | 1 (0.1%) | - | - | 1 (0.0%) | 2 (0.0%) |
| Cerebral haematoma | - | - | 1 (0.1%) | - | - | - | 1 (0.0%) |
| Cerebral haemorrhage | - | - | 3 (0.2%) | 1 (0.9%) | - | 18 (0.2%) | 22 (0.2%) |
| Cerebral infarction | - | - | 7 (0.6%) | - | - | 8 (0.1%) | 15 (0.2%) |
| Cerebrospinal fluid leakage | - | - | 2 (0.2%) | - | - | 4 (0.1%) | 6 (0.1%) |
| Cerebrovascular disorder | - | - | 1 (0.1%) | - | - | 3 (0.0%) | 4 (0.0%) |
| Chorea | - | - | 1 (0.1%) | - | - | 4 (0.1%) | 5 (0.1%) |
| Choreoathetosis | - | - | 4 (0.3%) | - | 1 (0.7%) | 4 (0.1%) | 9 (0.1%) |
| Circadian rhythm sleep disorder | - | - | 1 (0.1%) | - | - | 2 (0.0%) | 3 (0.0%) |
| Clonic convulsion | - | - | 3 (0.2%) | - | - | 3 (0.0%) | 6 (0.1%) |
| Clonus | - | - | 15 (1.2%) | - | - | 7 (0.1%) | 22 (0.2%) |
| Cognitive disorder | - | - | 6 (0.5%) | - | 3 (2.1%) | 63 (0.8%) | 72 (0.8%) |
| Cogwheel rigidity | - | - | 1 (0.1%) | - | - | 1 (0.0%) | 2 (0.0%) |
| Coordination abnormal | - | - | 1 (0.1%) | - | - | 22 (0.3%) | 23 (0.2%) |
| Cytotoxic oedema | - | - | 1 (0.1%) | - | - | - | 1 (0.0%) |
| Decerebrate posture | - | - | 1 (0.1%) | - | - | 1 (0.0%) | 2 (0.0%) |
| Decorticate posture | - | - | 1 (0.1%) | - | - | - | 1 (0.0%) |
| Diplegia | - | - | 2 (0.2%) | - | - | 3 (0.0%) | 5 (0.1%) |
| Dreamy state | - | - | 1 (0.1%) | - | - | 3 (0.0%) | 4 (0.0%) |
| Dyskinesia | - | - | 29 (2.4%) | - | 1 (0.7%) | 67 (0.9%) | 97 (1.0%) |
| Embolic stroke | - | - | 1 (0.1%) | - | - | - | 1 (0.0%) |
| Epidural lipomatosis | - | - | 1 (0.1%) | - | - | - | 1 (0.0%) |
| Epilepsy | - | - | 6 (0.5%) | 1 (0.9%) | - | 11 (0.1%) | 18 (0.2%) |
| Extensor plantar response | - | - | 2 (0.2%) | - | - | 1 (0.0%) | 3 (0.0%) |
| Extrapyramidal disorder | - | - | 8 (0.7%) | - | - | 7 (0.1%) | 15 (0.2%) |
| Facial paresis | - | - | 1 (0.1%) | - | - | - | 1 (0.0%) |
| Facial spasm | - | - | 1 (0.1%) | - | - | - | 1 (0.0%) |
| Febrile convulsion | - | - | 2 (0.2%) | - | - | - | 2 (0.0%) |
| Guillain-Barre syndrome | - | - | 1 (0.1%) | - | - | 1 (0.0%) | 2 (0.0%) |
| Head discomfort | - | - | 2 (0.2%) | 1 (0.9%) | - | 15 (0.2%) | 18 (0.2%) |
| Head titubation | - | - | 1 (0.1%) | - | - | 1 (0.0%) | 2 (0.0%) |
| Hemianopia | - | - | 1 (0.1%) | - | - | - | 1 (0.0%) |
| Hemiparesis | - | - | 4 (0.3%) | - | - | 12 (0.2%) | 16 (0.2%) |
| Hemiplegia | - | - | 4 (0.3%) | - | - | 8 (0.1%) | 12 (0.1%) |
| Hemiplegic migraine | - | - | 2 (0.2%) | - | - | - | 2 (0.0%) |
| Horner's syndrome | - | - | 7 (0.6%) | - | - | - | 7 (0.1%) |
| Hydrocephalus | - | - | 3 (0.2%) | 1 (0.9%) | - | 2 (0.0%) | 6 (0.1%) |
| Hyperkinesia | - | - | 2 (0.2%) | - | - | 2 (0.0%) | 4 (0.0%) |
| Hyperreflexia | - | - | 11 (0.9%) | - | - | 7 (0.1%) | 18 (0.2%) |
| Hypersomnia | - | - | 1 (0.1%) | - | 1 (0.7%) | 108 (1.4%) | 110 (1.2%) |
| Hypoglossal nerve disorder | - | - | 1 (0.1%) | - | - | - | 1 (0.0%) |
| Hypoglossal nerve paralysis | - | - | 1 (0.1%) | - | - | - | 1 (0.0%) |
| Hyporesponsive to stimuli | - | - | 2 (0.2%) | - | - | 1 (0.0%) | 3 (0.0%) |
| Hypoxic-ischaemic encephalopathy | - | - | 10 (0.8%) | - | - | 14 (0.2%) | 24 (0.3%) |
| IIIrd nerve paralysis | - | - | 1 (0.1%) | - | - | - | 1 (0.0%) |
| Intensive care unit acquired weakness | - | - | 2 (0.2%) | - | - | - | 2 (0.0%) |
| Intracranial pressure increased | - | - | 3 (0.2%) | - | - | 3 (0.0%) | 6 (0.1%) |
| Intraventricular haemorrhage | - | - | 2 (0.2%) | - | - | - | 2 (0.0%) |
| Judgement impaired | - | - | 3 (0.2%) | - | - | 2 (0.0%) | 5 (0.1%) |
| Language disorder | - | - | 1 (0.1%) | - | - | 11 (0.1%) | 12 (0.1%) |
| Locked-in syndrome | - | - | 1 (0.1%) | - | - | 1 (0.0%) | 2 (0.0%) |
| Monoparesis | - | - | 3 (0.2%) | - | - | 1 (0.0%) | 4 (0.0%) |
| Movement disorder | - | - | 9 (0.7%) | - | 1 (0.7%) | 19 (0.2%) | 29 (0.3%) |
| Multiple system atrophy | - | - | 1 (0.1%) | - | - | - | 1 (0.0%) |
| Muscle contractions involuntary | - | - | 8 (0.7%) | - | - | 6 (0.1%) | 14 (0.1%) |
| Muscle spasticity | - | - | 4 (0.3%) | - | - | 8 (0.1%) | 12 (0.1%) |
| Myasthenia gravis crisis | - | - | 2 (0.2%) | - | - | - | 2 (0.0%) |
| Myoclonic epilepsy | - | - | 2 (0.2%) | - | - | - | 2 (0.0%) |
| Myotonia | - | - | 1 (0.1%) | - | - | - | 1 (0.0%) |
| Neuralgia | - | - | 1 (0.1%) | 2 (1.9%) | - | 26 (0.3%) | 29 (0.3%) |
| Neuroleptic malignant syndrome | - | - | 16 (1.3%) | - | - | 6 (0.1%) | 22 (0.2%) |
| Neurological decompensation | - | - | 3 (0.2%) | - | - | - | 3 (0.0%) |
| Neurological symptom | - | - | 2 (0.2%) | - | 1 (0.7%) | 7 (0.1%) | 10 (0.1%) |
| Neuropathy peripheral | - | - | 1 (0.1%) | - | - | 54 (0.7%) | 55 (0.6%) |
| Nystagmus | - | - | 5 (0.4%) | - | - | 2 (0.0%) | 7 (0.1%) |
| Occipital neuralgia | - | - | 1 (0.1%) | - | - | - | 1 (0.0%) |
| Ophthalmic migraine | - | - | 1 (0.1%) | - | - | - | 1 (0.0%) |
| Opisthotonus | - | - | 1 (0.1%) | - | - | - | 1 (0.0%) |
| Optic neuritis | - | - | 1 (0.1%) | - | - | 1 (0.0%) | 2 (0.0%) |
| Orthostatic intolerance | - | - | 1 (0.1%) | - | - | - | 1 (0.0%) |
| Paresis | - | - | 1 (0.1%) | - | - | 4 (0.1%) | 5 (0.1%) |
| Parkinsonism | - | - | 1 (0.1%) | - | - | 6 (0.1%) | 7 (0.1%) |
| Parosmia | - | - | 2 (0.2%) | - | - | 10 (0.1%) | 12 (0.1%) |
| Partial seizures | - | - | 2 (0.2%) | - | - | 2 (0.0%) | 4 (0.0%) |
| Peripheral nerve palsy | - | - | 1 (0.1%) | - | - | - | 1 (0.0%) |
| Petit mal epilepsy | - | - | 1 (0.1%) | - | 1 (0.7%) | 5 (0.1%) | 7 (0.1%) |
| Phrenic nerve paralysis | - | - | 1 (0.1%) | - | - | - | 1 (0.0%) |
| Posterior reversible encephalopathy syndrome | - | - | 3 (0.2%) | - | - | 3 (0.0%) | 6 (0.1%) |
| Postresuscitation encephalopathy | - | - | 1 (0.1%) | - | - | - | 1 (0.0%) |
| Psychogenic seizure | - | - | 1 (0.1%) | - | - | 1 (0.0%) | 2 (0.0%) |
| Radiculopathy | - | - | 7 (0.6%) | - | 1 (0.7%) | 3 (0.0%) | 11 (0.1%) |
| Retrograde amnesia | - | - | 4 (0.3%) | - | - | - | 4 (0.0%) |
| Seizure cluster | - | - | 1 (0.1%) | - | - | - | 1 (0.0%) |
| Seizure like phenomena | - | - | 1 (0.1%) | - | - | - | 1 (0.0%) |
| Sensorimotor disorder | - | - | 1 (0.1%) | - | - | - | 1 (0.0%) |
| Sensory disturbance | - | - | 4 (0.3%) | - | - | 19 (0.2%) | 23 (0.2%) |
| Sensory loss | - | - | 2 (0.2%) | - | - | 14 (0.2%) | 16 (0.2%) |
| Slow response to stimuli | - | - | 1 (0.1%) | - | 2 (1.4%) | 7 (0.1%) | 10 (0.1%) |
| Small fibre neuropathy | - | - | 1 (0.1%) | - | - | - | 1 (0.0%) |
| Spinal cord compression | - | - | 1 (0.1%) | - | - | 9 (0.1%) | 10 (0.1%) |
| Spinal cord disorder | - | - | 1 (0.1%) | - | - | 1 (0.0%) | 2 (0.0%) |
| Spinal cord infarction | - | - | 1 (0.1%) | - | - | - | 1 (0.0%) |
| Spinal epidural haematoma | - | - | 1 (0.1%) | - | - | - | 1 (0.0%) |
| Status epilepticus | - | - | 5 (0.4%) | - | - | 7 (0.1%) | 12 (0.1%) |
| Subacute combined cord degeneration | - | - | 1 (0.1%) | - | - | - | 1 (0.0%) |
| Subarachnoid haemorrhage | - | - | 2 (0.2%) | - | - | 2 (0.0%) | 4 (0.0%) |
| Sudden onset of sleep | - | - | 2 (0.2%) | - | - | 2 (0.0%) | 4 (0.0%) |
| Tardive dyskinesia | - | - | 2 (0.2%) | - | - | 3 (0.0%) | 5 (0.1%) |
| Tonic clonic movements | - | - | 4 (0.3%) | - | - | 2 (0.0%) | 6 (0.1%) |
| Tonic convulsion | - | - | 2 (0.2%) | - | - | 5 (0.1%) | 7 (0.1%) |
| Toxic encephalopathy | - | - | 2 (0.2%) | - | 1 (0.7%) | 11 (0.1%) | 14 (0.1%) |
| Toxic leukoencephalopathy | - | - | 2 (0.2%) | - | - | 1 (0.0%) | 3 (0.0%) |
| Trigeminal nerve disorder | - | - | 1 (0.1%) | - | - | - | 1 (0.0%) |
| Trigemino-cardiac reflex | - | - | 1 (0.1%) | - | - | - | 1 (0.0%) |
| Uraemic encephalopathy | - | - | 1 (0.1%) | - | - | - | 1 (0.0%) |
| Vocal cord paralysis | - | - | 2 (0.2%) | - | - | - | 2 (0.0%) |
| Vocal cord paresis | - | - | 1 (0.1%) | - | - | - | 1 (0.0%) |
| Carotid artery stenosis | - | - | - | 1 (0.9%) | - | 1 (0.0%) | 2 (0.0%) |
| Cervical radiculopathy | - | - | - | 1 (0.9%) | - | 1 (0.0%) | 2 (0.0%) |
| Cluster headache | - | - | - | 1 (0.9%) | - | 3 (0.0%) | 4 (0.0%) |
| Drop attacks | - | - | - | 1 (0.9%) | - | 4 (0.1%) | 5 (0.1%) |
| Ischaemic stroke | - | - | - | 1 (0.9%) | - | 2 (0.0%) | 3 (0.0%) |
| Dementia Alzheimer's type | - | - | - | - | 1 (0.7%) | 6 (0.1%) | 7 (0.1%) |
| Encephalomalacia | - | - | - | - | 1 (0.7%) | 1 (0.0%) | 2 (0.0%) |
| Amyotrophic lateral sclerosis | - | - | - | - | - | 4 (0.1%) | 4 (0.0%) |
| Apraxia | - | - | - | - | - | 4 (0.1%) | 4 (0.0%) |
| Atonic seizures | - | - | - | - | - | 1 (0.0%) | 1 (0.0%) |
| Autonomic neuropathy | - | - | - | - | - | 2 (0.0%) | 2 (0.0%) |
| Basal ganglia infarction | - | - | - | - | - | 1 (0.0%) | 1 (0.0%) |
| Bell's palsy | - | - | - | - | - | 1 (0.0%) | 1 (0.0%) |
| Brain stem infarction | - | - | - | - | - | 1 (0.0%) | 1 (0.0%) |
| Brain stem ischaemia | - | - | - | - | - | 1 (0.0%) | 1 (0.0%) |
| Brain stem syndrome | - | - | - | - | - | 1 (0.0%) | 1 (0.0%) |
| Carotid artery dissection | - | - | - | - | - | 1 (0.0%) | 1 (0.0%) |
| Carotid artery occlusion | - | - | - | - | - | 2 (0.0%) | 2 (0.0%) |
| Carpal tunnel syndrome | - | - | - | - | - | 16 (0.2%) | 16 (0.2%) |
| Cataplexy | - | - | - | - | - | 1 (0.0%) | 1 (0.0%) |
| Cauda equina syndrome | - | - | - | - | - | 1 (0.0%) | 1 (0.0%) |
| Central-alveolar hypoventilation | - | - | - | - | - | 1 (0.0%) | 1 (0.0%) |
| Cerebellar syndrome | - | - | - | - | - | 1 (0.0%) | 1 (0.0%) |
| Cerebral atrophy | - | - | - | - | - | 7 (0.1%) | 7 (0.1%) |
| Cerebral cyst | - | - | - | - | - | 1 (0.0%) | 1 (0.0%) |
| Cerebral disorder | - | - | - | - | - | 8 (0.1%) | 8 (0.1%) |
| Cerebral hypoperfusion | - | - | - | - | - | 1 (0.0%) | 1 (0.0%) |
| Cerebral ischaemia | - | - | - | - | - | 5 (0.1%) | 5 (0.1%) |
| Cerebral small vessel ischaemic disease | - | - | - | - | - | 1 (0.0%) | 1 (0.0%) |
| Cerebral thrombosis | - | - | - | - | - | 1 (0.0%) | 1 (0.0%) |
| Cholinergic syndrome | - | - | - | - | - | 2 (0.0%) | 2 (0.0%) |
| Clumsiness | - | - | - | - | - | 4 (0.1%) | 4 (0.0%) |
| Coma hepatic | - | - | - | - | - | 1 (0.0%) | 1 (0.0%) |
| Complex regional pain syndrome | - | - | - | - | - | 12 (0.2%) | 12 (0.1%) |
| Consciousness fluctuating | - | - | - | - | - | 7 (0.1%) | 7 (0.1%) |
| Convulsions local | - | - | - | - | - | 2 (0.0%) | 2 (0.0%) |
| Dementia | - | - | - | - | - | 28 (0.4%) | 28 (0.3%) |
| Demyelinating polyneuropathy | - | - | - | - | - | 2 (0.0%) | 2 (0.0%) |
| Demyelination | - | - | - | - | - | 2 (0.0%) | 2 (0.0%) |
| Diabetic coma | - | - | - | - | - | 1 (0.0%) | 1 (0.0%) |
| Diabetic neuropathy | - | - | - | - | - | 8 (0.1%) | 8 (0.1%) |
| Dizziness postural | - | - | - | - | - | 4 (0.1%) | 4 (0.0%) |
| Drug withdrawal headache | - | - | - | - | - | 1 (0.0%) | 1 (0.0%) |
| Dysgraphia | - | - | - | - | - | 5 (0.1%) | 5 (0.1%) |
| Dyslexia | - | - | - | - | - | 2 (0.0%) | 2 (0.0%) |
| Dyspraxia | - | - | - | - | - | 1 (0.0%) | 1 (0.0%) |
| Dysstasia | - | - | - | - | - | 27 (0.4%) | 27 (0.3%) |
| Electric shock sensation | - | - | - | - | - | 6 (0.1%) | 6 (0.1%) |
| Embolic cerebral infarction | - | - | - | - | - | 1 (0.0%) | 1 (0.0%) |
| Facial nerve disorder | - | - | - | - | - | 1 (0.0%) | 1 (0.0%) |
| Fine motor skill dysfunction | - | - | - | - | - | 1 (0.0%) | 1 (0.0%) |
| Focal dyscognitive seizures | - | - | - | - | - | 2 (0.0%) | 2 (0.0%) |
| Frontotemporal dementia | - | - | - | - | - | 1 (0.0%) | 1 (0.0%) |
| Generalised onset non-motor seizure | - | - | - | - | - | 2 (0.0%) | 2 (0.0%) |
| Gliosis | - | - | - | - | - | 1 (0.0%) | 1 (0.0%) |
| Gross motor delay | - | - | - | - | - | 1 (0.0%) | 1 (0.0%) |
| Haemorrhage intracranial | - | - | - | - | - | 2 (0.0%) | 2 (0.0%) |
| Haemorrhagic stroke | - | - | - | - | - | 1 (0.0%) | 1 (0.0%) |
| Hypercapnic coma | - | - | - | - | - | 9 (0.1%) | 9 (0.1%) |
| Hypoglycaemic coma | - | - | - | - | - | 1 (0.0%) | 1 (0.0%) |
| Hypoglycaemic seizure | - | - | - | - | - | 1 (0.0%) | 1 (0.0%) |
| Hypoglycaemic unconsciousness | - | - | - | - | - | 1 (0.0%) | 1 (0.0%) |
| Idiopathic intracranial hypertension | - | - | - | - | - | 1 (0.0%) | 1 (0.0%) |
| Intracranial haematoma | - | - | - | - | - | 1 (0.0%) | 1 (0.0%) |
| Intracranial hypotension | - | - | - | - | - | 1 (0.0%) | 1 (0.0%) |
| Lacunar infarction | - | - | - | - | - | 2 (0.0%) | 2 (0.0%) |
| Leukoencephalopathy | - | - | - | - | - | 14 (0.2%) | 14 (0.1%) |
| Lumbar radiculopathy | - | - | - | - | - | 2 (0.0%) | 2 (0.0%) |
| Meralgia paraesthetica | - | - | - | - | - | 1 (0.0%) | 1 (0.0%) |
| Multiple sclerosis relapse | - | - | - | - | - | 3 (0.0%) | 3 (0.0%) |
| Myasthenia gravis | - | - | - | - | - | 4 (0.1%) | 4 (0.0%) |
| Narcolepsy | - | - | - | - | - | 9 (0.1%) | 9 (0.1%) |
| Nerve compression | - | - | - | - | - | 10 (0.1%) | 10 (0.1%) |
| Nerve degeneration | - | - | - | - | - | 1 (0.0%) | 1 (0.0%) |
| Neuritis | - | - | - | - | - | 1 (0.0%) | 1 (0.0%) |
| Neuromyopathy | - | - | - | - | - | 2 (0.0%) | 2 (0.0%) |
| Neurosarcoidosis | - | - | - | - | - | 2 (0.0%) | 2 (0.0%) |
| Paralysis recurrent laryngeal nerve | - | - | - | - | - | 1 (0.0%) | 1 (0.0%) |
| Parkinson's disease | - | - | - | - | - | 13 (0.2%) | 13 (0.1%) |
| Parkinsonian crisis | - | - | - | - | - | 1 (0.0%) | 1 (0.0%) |
| Patient elopement | - | - | - | - | - | 4 (0.1%) | 4 (0.0%) |
| Peripheral paralysis | - | - | - | - | - | 1 (0.0%) | 1 (0.0%) |
| Peripheral sensorimotor neuropathy | - | - | - | - | - | 1 (0.0%) | 1 (0.0%) |
| Peroneal nerve palsy | - | - | - | - | - | 1 (0.0%) | 1 (0.0%) |
| Post herpetic neuralgia | - | - | - | - | - | 1 (0.0%) | 1 (0.0%) |
| Radial nerve palsy | - | - | - | - | - | 5 (0.1%) | 5 (0.1%) |
| Reduced facial expression | - | - | - | - | - | 1 (0.0%) | 1 (0.0%) |
| Reflexes abnormal | - | - | - | - | - | 4 (0.1%) | 4 (0.0%) |
| Repetitive speech | - | - | - | - | - | 2 (0.0%) | 2 (0.0%) |
| Ruptured cerebral aneurysm | - | - | - | - | - | 1 (0.0%) | 1 (0.0%) |
| Sleep deficit | - | - | - | - | - | 3 (0.0%) | 3 (0.0%) |
| Slow speech | - | - | - | - | - | 3 (0.0%) | 3 (0.0%) |
| Spinal cord oedema | - | - | - | - | - | 1 (0.0%) | 1 (0.0%) |
| Spinal cord paralysis | - | - | - | - | - | 1 (0.0%) | 1 (0.0%) |
| Supranuclear palsy | - | - | - | - | - | 1 (0.0%) | 1 (0.0%) |
| Tension headache | - | - | - | - | - | 1 (0.0%) | 1 (0.0%) |
| Thermohypoaesthesia | - | - | - | - | - | 1 (0.0%) | 1 (0.0%) |
| Thoracic outlet syndrome | - | - | - | - | - | 1 (0.0%) | 1 (0.0%) |
| Thoracic spinal cord paralysis | - | - | - | - | - | 1 (0.0%) | 1 (0.0%) |
| Tongue biting | - | - | - | - | - | 1 (0.0%) | 1 (0.0%) |
| Tongue paralysis | - | - | - | - | - | 1 (0.0%) | 1 (0.0%) |
| Trigeminal neuralgia | - | - | - | - | - | 2 (0.0%) | 2 (0.0%) |
| Tunnel vision | - | - | - | - | - | 1 (0.0%) | 1 (0.0%) |
| Ulnar nerve palsy | - | - | - | - | - | 1 (0.0%) | 1 (0.0%) |
| Vascular dementia | - | - | - | - | - | 2 (0.0%) | 2 (0.0%) |

**
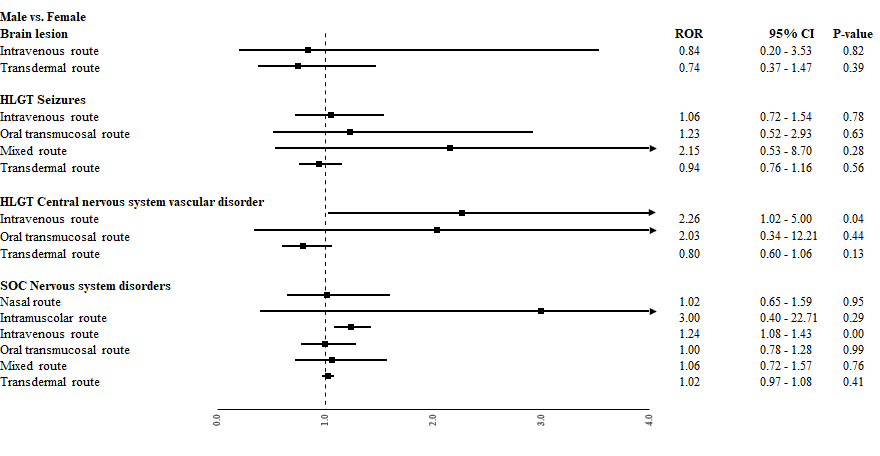
**

**Supplementary Figure 2.** Reporting odds ratio (ROR) for brain lesions, High-Level Group Term (HLGT) 'central nervous system vascular disorders', HLGT 'seizures', and system organ class (SOC) level 'nervous system disorders', comparing males and females across different routes of fentanyl administration. RORs and their 95% confidence intervals (CI) were calculated for individual events and presented both graphically as Forrest plot (black square with line) and numerically.
